# Full-spectrum cannabis extracts for women with chronic pain syndromes: a real-life retrospective report of multi-symptomatic benefits after treatment with individually tailored dosage schemes

**DOI:** 10.3389/fphar.2025.1538518

**Published:** 2025-11-20

**Authors:** Patrícia Montagner Soares Silva, Wesley Medeiros, Clarissa Nogueira Borges, Joaquim P. Brasil-Neto, Wilson Lessa Jr., Ricardo Ferreira de Oliveira e Silva, Fabio V. Caixeta, Renato Malcher-Lopes

**Affiliations:** 1 NeuroVinci, São José, Brazil; 2 Department of Physiological Sciences, University of Brasilia, Brasília, Brazil; 3 State Secretariat of Education of the Federal District, Brasília, Brazil; 4 Medical School, Centro Universitário Unieuro, Brasília, Brazil; 5 Health Sciences Center, Federal University of Paraíba, Paraíba, Brazil; 6 Vertebralis Spine Center, Rio de Janeiro, Brazil

**Keywords:** cannabis, cannabinoids, chronic pain, migraine, fibromyalgia, clinical outcome, women

## Abstract

Chronic pain syndromes (CPS) are debilitating conditions for which cannabis extracts and cannabinoids have shown promise as effective treatments. However, accessibility to these treatments is limited due to the absence of suitable formulations and standardized dosage guidelines. This is particularly critical for women, who present sex-specific differences in pain burden, pain perception, and pain-related cannabinoid pharmacology. We conducted a retrospective open-label cross-sectional study on 29 female CPS patients who received full-spectrum cannabis extracts (FCEs) with standardized compositions produced by two patient-led civil societies. An individually tailored dosage protocol was used, with dosage schemes adjusted based on individualized clinical assessments of initial conditions and treatment responses. Patients received either CBD-dominant extracts, THC-dominant extracts, or a combination of both. To evaluate the results, we conducted a comprehensive online patient-reported outcome survey covering core CPS symptoms, comorbidities, personal burden, and quality of life—including open-ended questions to capture the practical and subjective impacts of CPS and FCEs treatment on patients’ lives. Despite most patients already using medications for pain and mood disorders, all reported some level of pain relief, and most reported improvements in cognitive function, motor abilities, professional activities, irritability, anxiety, melancholy, fatigue, and sleep quality. Qualitative content analysis of open-ended responses revealed that FCEs had relevant positive effects on practical and subjective domains, as well as personal relationships. No patients had to discontinue extract use due to adverse effects, and most reduced or ceased their use of analgesic and psychiatric medications. The optimal dosage regime, including CBD-to-THC proportions, was established through a response-based protocol, varied considerably, and showed no clear link to specific pain types. These real-life results strongly suggest that a broad scope of benefits can be achieved by using flexible dosing schemes of cannabis extracts in managing diverse CPS conditions in female patients. Therefore, this study highlights the significance of tailoring treatment plans to individual CPS cases. Moreover, it demonstrates the feasibility of utilizing quality-controlled cannabis extracts produced by civil societies as either adjuncts or primary pharmacotherapeutic options in CPS management.

## 1 Introduction

Any pain that lasts or recurs for longer than 3 months can be classified as Chronic Pain (Treede et al., 2015; Treede et al., 2019), which is considered a multifactorial, biopsychosocial syndrome (WHO, 2004; Leo, 2005). The International Classification of Diseases 11 (ICD-11) has classified this disorder into seven main subgroups, that have also been called Chronic Pain Syndromes (CPS): 1) chronic primary pain (i.e., fibromyalgia or non-specific back pain), in which pain has no discernible origin, 2) neuropathic pain, 3) secondary musculoskeletal pain, 4) cancer pain, 5) postsurgical pain, 6) headache and orofacial pain) and 7) secondary visceral pain (Sirianni et al., 2015; Kashikar-Zuck et al., 2016; Biz et al., 2021; Midenfjord et al., 2021; Seidel et al., 2022; Yasaei et al., 2022).

Global prevalence of chronic pain is around 20% (Goldberg and McGee, 2011), 27% in Europe (Leadley et al., 2012), 20% in the United States (Yong et al., 2022) and 33% in Africa, Asia and Latin America (Jackson et al., 2015). In Brazil, the prevalence of chronic pain among adults is estimated to range from 23.0% to 41.4%, with higher rates observed in women (Santiago et al., 2023). Other studies also indicate higher prevalence in women (Reitsma et al., 2012). CPS are the lead cause of years lived with disability globally (Rice et al., 2016). Its overall burden includes important secondary impacts, such as depression and anxiety (Castro et al., 2009; Chen et al., 2012; Gonzalez-Sepulveda et al., 2016; Kawai et al., 2017), impaired enjoyment of life (Cohen et al., 2010; Kawai et al., 2017), loss of professional productivity (Kawai et al., 2017) and higher rate of absence from work (Leadley et al., 2012; Duenas et al., 2016), leading to personal, finantial and social impacts as well. Further, social benefits and health services for people removed from work due to CPS represent billions of dollars/euros from the community’s budget (Phillips, 2006; 2009; Groenewald et al., 2014). All those aspects should be considered for better diagnosis and globally effective treatments (Goldberg and McGee, 2011; Treede et al., 2015; Treede et al., 2019).

Current treatments involve oral analgesics, weak-to-strong opioids, antidepressants and mood stabilizers (Sirianni et al., 2015). The “three-step analgesic ladder” is a commonly used guideline for pain treatment issued by the World Health Organization (Reid and Davies, 2004; Fallon et al., 2022); however, it is often not enough to provide long-lasting relieve (Hylands-White et al., 2017). Conventional treatments may cause side effects such as dizziness, nausea, renal problems, and even overdose (Thomas et al., 2015; Delle and Gazley, 2021). Degenerative CPS, for instance, have long-term pharmacological treatment limited mainly to non-steroidal anti-inflammatory drugs or opioids (Seidel et al., 2022). However, long-term opioid therapy is not always effective, and its discontinuation may lead to severe pain worsening (Manhapra, 2022). Furthermore, problematic use of opioids is a particular concern in CPS treatment (Vowles et al., 2015).

Studies with isolated cannabinoids revealed relief of chronic pain, inflammation, depression, and other CPS-associated comorbidities in animal models (Rodriguez-Munoz et al., 2018; Berger et al., 2019; Campos et al., 2021; Razavi et al., 2021; Vigli et al., 2021; Zhang L. et al., 2021; Zieglgansberger et al., 2022). Isolated cannabidiol (CBD) has shown analgesic and anti-inflammatory effects in humans, while tetrahydrocannabinol (THC) seems to produce pain relief by modulating neuronal activity in pain-associated areas of the central nervous system, such as the periaqueductal area (Lee G. et al., 2018), and the descending supraspinal inhibitory pathways, often involved in cases of CPS (Baron, 2018; Vazquez-Leon et al., 2021). Accordingly, THC isolated oil promoted significant relief of chronic neuropathic pain in comparison to placebo (Weizman et al., 2018).

In 2017, a panel convened by the U.S. National Academies of Sciences, Engineering, and Medicine concluded that cannabis-based medicines are effective for the treatment of chronic pain (National Academies of Sciences and Medicine, 2017). This conclusion was based on a range of studies, including a systematic review of randomized controlled trials on cannabinoid treatments for rheumatic diseases (Fitzcharles et al., 2016) and a meta-analysis on the effectiveness of inhaled cannabis for chronic neuropathic pain (Andreae et al., 2015). Additionally, a randomized, double-blind, placebo-controlled clinical trial using a THC-rich, full-spectrum cannabis extract (FCE) demonstrated significant pain relief in patients with fibromyalgia compared to placebo (Chaves et al., 2020). FCE has also shown effectiveness in alleviating cancer-related pain (Bar-Lev Schleider et al., 2018).

Epidemiological and clinical studies on the medical use of cannabis have also shown significant improvement in anxiety, social relations, movement, sleep quality and other CPS-associated comorbidities (Jones et al., 1981; Pertwee, 1999; Russo et al., 2007; Aggarwal et al., 2009; Avello et al., 2017; Kosiba et al., 2019; Lake et al., 2020; Li et al., 2020; Stith et al., 2020; Feinstein et al., 2021; Khurshid et al., 2021; Aebischer et al., 2022; Berger et al., 2022; Kuhathasan et al., 2022; Leung et al., 2022; Sachedina et al., 2022)

A recent systematic review on long-term side effects of cannabis and cannabinoid treatment for chronic pain indicated that serious adverse events, adverse events leading to discontinuation, cognitive adverse events, accidents and injuries, and dependence and withdrawn syndrome occur in fewer than 1 in 20 patients (Zeraatkar et al., 2022). Caution is required, though, as the outcome of cannabis-based medicines for the treatment of mood disorders and anxiety seems to be related to the proportional content of CBD to THC and varies among individuals (Berger et al., 2022).

While robust scientific evidence supports the efficacy of cannabis extracts and cannabinoids in treating Chronic Pain Syndrome (CPS) (Jugl et al., 2021), widespread access to this approach is hindered due to a lack of standardized dosage guidelines and limited availability of suitable formulations. This is particularly pronounced when treating different CPS conditions in female patients. Both pre-clinical and human studies have revealed sex-specific, pain-related cannabinoid pharmacology (Fattore and Fratta, 2010; Blanton et al., 2021; Santoro et al., 2021). Notably, pain perception, coping strategies, and pharmacological sensitivity to medications in general differ between male and female patients (Fillingim, 2002; Frot et al., 2004; Garofalo et al., 2006; Gallagher, 2010; Gazerani et al., 2021; Failla et al., 2023). Risk factors for several CPS conditions include pregnancy, motherhood, and female hormonal changes throughout life (Wijnhoven et al., 2006; Fattore and Fratta, 2010). Furthermore, in various parts of the world, from Brazil to Sweden, women managing CPS often contend with an additional burden associated with caregiving and household activities, even when unwell (Brazilian-Institute-of-Geography-and-Statistics, 2021; Melander, 2023).

**FIGURE 1:**
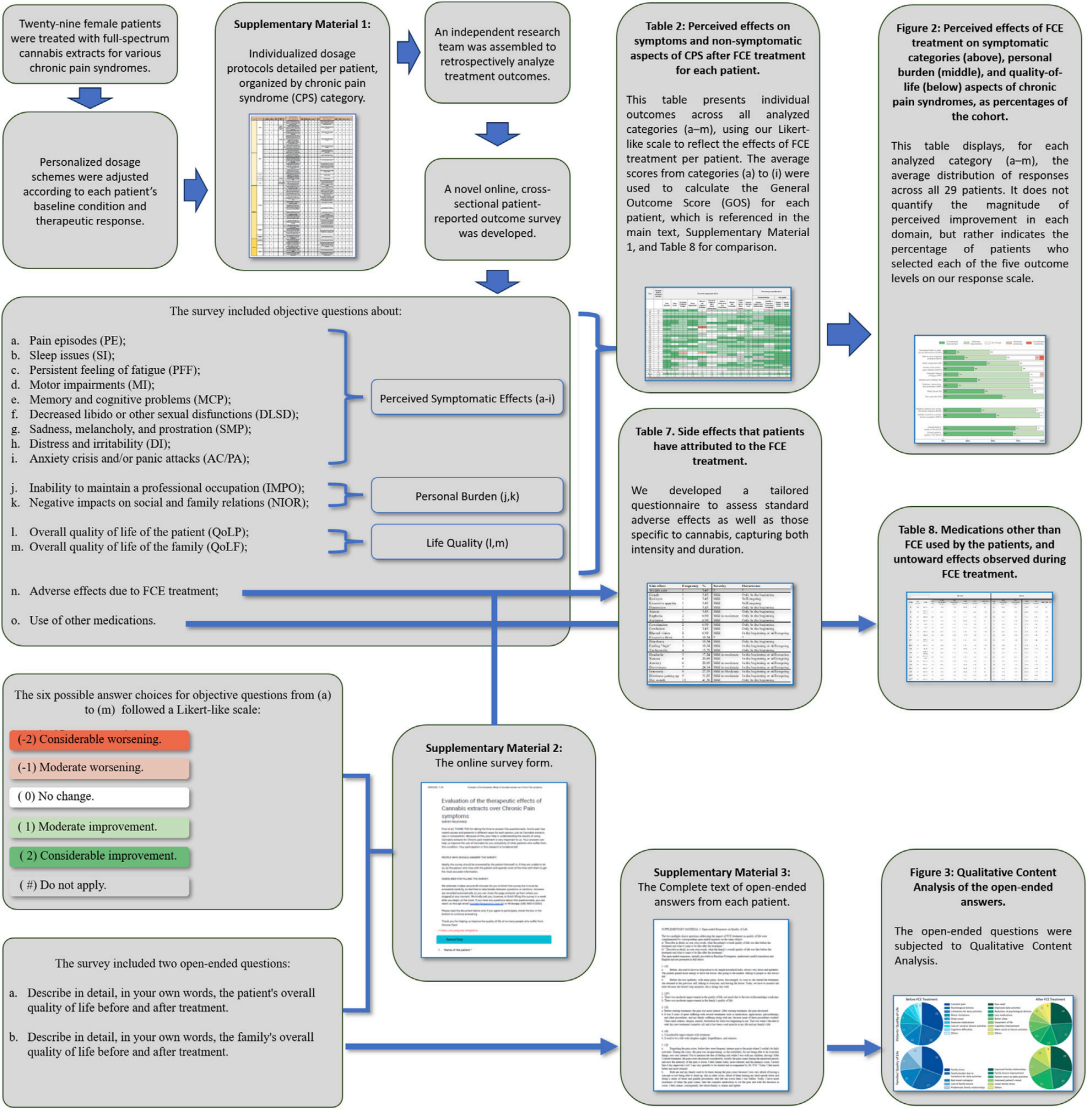
Schematic summary of this report.

Most industrial cannabis extracts are primarily designed for epilepsy and are not suitable for treating chronic pain syndromes (CPS). In Brazil, THC-rich formulations are scarce, imported, and unaffordable for most patients. In response, Brazilian patients have formed civil societies to produce and distribute standardized, quality-controlled full-spectrum cannabis extracts (FCEs). Here, we report an open-label retrospective study evaluating the outcomes of 29 Brazilian women with CPS who were treated with FCEs from two such civil societies. In most cases, FCEs were initially introduced as adjuvants, as patients were not fully satisfied with the outcomes achieved using standard pharmacological protocols. Treatment followed a flexible, individualized titration approach, allowing adjustments in CBD and THC proportions according to each patient’s condition and response. Depending on clinical assessments, patients received CBD-dominant, THC-dominant, or mixed formulations. This protocol originated from real-life clinical practice (by one of the authors) and was later independently analysed by our research team, following the steps summarized in Figure 1.

We employed a comprehensive online cross-sectional survey to assess patient-reported outcomes, covering both core CPS symptoms and comorbidities. All participants reported reductions in pain and psychological distress, as well as improvements in functionality and quality of life—both personally and within their families. Most also reduced or ceased their use of other medications for pain and mood disorders. Open-ended responses further highlighted the broad, practical impact of CPS and FCEs treatment on daily life and wellbeing.

## 2 Methods

### 2.1 Participants

Treatment with FCE was conducted by one of the authors (PMSS) in the clinical setting. Either during or after the end of each patient’s treatment, patients (and, when needed patient’s parents) were invited to take part in this study by answering an online cross-sectional, patient-reported outcome survey.

Invitations were sent to 178 patients (53 males and 125 females), yielding responses from 48 individuals. To minimize formulation variability, we focused on patients using formulations from the same two Patient Societies, resulting in a sample of 30 patients, 29 females and one male. Initially, we analysed data for all 30 patients; however, after further consideration, we decided to focus solely on female participants. To maintain consistency with our original ID system, we retained the numerical identifiers followed by the letter “f” to indicate female patients, ensuring accuracy when correlating data with our raw dataset. Specifically, the male participant was identified as “24 m,” while the female participants included in this report were labelled sequentially as ”1f,” “2f,” and so on, up to “23f,” then continuing from “25f” to “30f”. No action was taken to influence the sample composition in terms of CPS etiology. The 29 remaining participants, diagnosed with various ICD-11 designations for CPS, willingly enrolled in this report. Participation involved granting access to their clinical records (Supplementary Material S1) and responding to the patient-reported outcome survey (Supplementary Material S2).

### 2.2 Treatment

Our cohort encompassed patients with diverse chronic pain etiologies and varied access to FCE formulations (whole *Cannabis sativa L.* inflorescence extract), leading to the individualized tailoring of the treatment protocol concerning FCE formulations and dosages. Initial formulation selection was guided by the patient’s main symptoms, comorbidities, and prior treatment history: CBD-dominant extracts were generally chosen for inflammatory or nociplastic pain, anxiety, seizure history, or metabolic concerns, whereas THC-dominant extracts were indicated when insomnia, nausea, muscle spasms, or insufficient analgesia under CBD-dominant regimens were prominent.

Treatment began with minimal dosages—typically 1 to 3 drops/day (corresponding to approximately 2–7 mg/day CBD or 0.5–2 mg/day THC, depending on extract concentration)—and was titrated upward every few days according to patient-reported symptom relief and side-effect profile. Dosages were fine-tuned over two to 4 weeks, with adjustments aiming to maximize benefit while avoiding undesirable side effects. Stabilization was achieved through consensus between physician and patient. In most cases, this resulted in maintenance doses of 15–30 drops/day (roughly 22–37 mg/day CBD and 8–11 mg/day THC), administered orally in three daily doses.

Dosage increments typically continued until either: 1) target symptom control was achieved, or 2) side effects emerged, prompting dose stabilization or reduction. When a single extract did not provide adequate improvement based on these criteria, a second extract with complementary cannabinoid dominance was introduced at a low dose, and the CBD: THC ratio was progressively adjusted until an optimal balance between efficacy and tolerability was reached. The decision to combine extracts was based on persistent symptoms (e.g., poor sleep, anxiety, residual pain) known, from pharmacological rationale and prior clinical experience, to respond to the other cannabinoid profile (e.g., adding THC-dominant extract for refractory insomnia or adding CBD-dominant extract to mitigate THC-related anxiety).

Monthly evaluations were conducted for a trimester, followed by six-monthly assessments once symptoms were stabilized; in exceptional cases, weekly reassessments and adjustments were necessary. The average CBD concentration at the treatment’s outset was 22.52 mg/day, increasing to 47.14 mg/day at the end, while the average THC concentration started at 8.23 mg/day and reached 11.32 mg/day at the conclusion. The average CBD: THC proportion for all patients was roughly 3.3:1. Ultimately, each patient arrived at an individualized dosage regimen (Table 1), as comprehensively described in Supplementary Material S1. In cases where treatment involved more than one oil type at the same time, final dosage of each cannabinoid was calculated adding their respective content per ml of each extract. All adjustments, including reasons for switching or combining extracts, are detailed in Supplementary Material S1, which shows for each patient the initial choice, criteria for change, and final stabilized regimen.

**TABLE 1:** Cohort description, weight, and FCE dosage for each patient at the beginning and at the end of treatment. ICD-11 codes and descriptions are Coxarthrosis (M16), Pain in joint (M25.5), Sacrococcygeal disorders (M53.3), Dorsalgia (M54), Cervicalgia (M54.2), Lumbago with sciatica (M54.4), Low back pain (M54.5), Myalgia (M79.1), Pain in limb (M79.6), Fibromyalgia (M79.7), Soft tissue disorder (M79.9), Other disorders of bone (M89), Migraine without aura (G43) and Nerve root and plexus disorders (G54).

			Initial						Final					
Case	Age (years)	ICD	Wgt (kg)	CBD (mg/kg/day) (x10^−1^)	THC (mg/kg/day) (x10^−1^)	CBD (mg/day)	THC (mg/day)	CBD:THC (ratio)	Wgt (kg)	CBD (mg/kg/day) (x10^−1^)	THC (mg/kg/day) (x10^−1^)	CBD (mg/day)	THC (mg/day)	CBD:THC ratio
1f	84	M.79.7	85	7.06	1.76	60.00	15.00	4:1	87	8.05	2.01	70.00	17.50	4:1
2f^#^	56	M25.5, M79.7	75	0.03	1.20	0.23	9.00	1:38	69	13.04	3.26	90.00	22.50	4:1
3f	43	M89.0	88	0.01	0.57	0.13	5.00	1:38	88	0.03	1.02	0.23	9.00	1:38
4f	70	M25.5	62	7.26	1.81	45.00	11.25	4:1	59	12.71	3.18	75.00	18.75	4:1
5f	40	G43	55	6.82	0.33	37.50	1.80	21:1	57	6.58	0.32	37.50	1.80	21:1
6f	45	G43	60	0.02	0.75	0.12	4.50	1:38	63	0.02	0.71	0.12	4.50	1:38
7f	38	M79.7	104	0.02	0.72	0.20	7.50	1:38	107	0.02	0.70	0.20	7.50	1:38
8f	72	M89.0	64	5.86	0.28	37.50	1.80	21:1	64	5.86	0.28	37.50	1.80	21:1
9f*^&#^	47	M54.2, M79.1	66	0.02	0.91	0.16	6.00	1:38	60	5.05	1.24	30.27	7.44	4:1
10f*^&#^	56	M.79.7	50	0.04	1.50	0.20	7.50	1:38	45	0.08	1.67	0.34	7.50	1:22
11f	61	M.54	70	6.43	1.61	45.00	11.25	4:1	70	6.43	1.61	45.00	11.25	4:1
12f	61	M54.5, M54.4	80	4.69	0.23	37.50	1.80	21:1	80	6.56	0.32	52.50	2.52	21:1
13f	49	M.25.5, C50	82	0.02	0.91	0.20	7.50	1:38	82	0.02	0.91	0.20	7.50	1:38
14f	60	M79.7	76	4.93	0.24	37.50	1.80	21:1	69	5.43	0.26	37.50	1.80	21:1
15f	43	M79.7	103	4.85	1.21	50.00	12.50	4:1	98	7.14	1.79	70.00	17.50	4:1
16f*^#^	27	M79.7	65	6.92	1.73	45.00	11.25	4:1	65	9.25	3.23	60.16	21.00	3:1
17f	62	M53.3	83	5.42	1.36	45.00	11.25	4:1	81	5.56	1.39	45.00	11.25	4:1
18f*^&#^	65	M79.7	70	0.04	1.50	0.27	10.50	1:38	67	4.54	1.52	37.91	10.80	3:1
19f*^&^	42	M79.7	42	0.08	1.67	0.32	7.00	1:22	45	10.14	3.57	45.63	16.07	3:1
20f*	69	M16, M25.5	73	6.18	1.95	45.14	14.25	3:1	73	6.18	1.95	45.14	14.25	3:1
21f*^#^	65	G43	49	0.09	3.37	0.43	16.50	1:38	56	0.27	0.11	15.16	6.31	2:1
22f^&#^	35	M79.7	62	2.42	0.60	15.00	0.72	4:1	62	5.43	0.26	37.50	1.8	21:1
23f	72	M79.7, M.25.5	70	0.02	0.86	0.16	6.00	1:38	65	0.05	1.85	0.31	12.00	1:38
25f	54	M54.4	90	6.67	1.67	60.00	15.00	4:1	90	7.78	1.99	60.00	15.00	4:1
26f	42	G43	65	6.92	1.73	45.00	11.25	4:1	65	9.23	2.31	60.00	15.00	4:1
27f^#^	44	M79.1	92	0.03	0.65	0.16	6.00	4:1	80	0.02	0.75	0.16	6.00	1:38
28f	51	G54	53	0.04	1.70	0.23	9.00	1:38	53	0.09	3.40	0.47	18.00	1:38
29f	31	G43	50	9.00	2.25	45.00	11.25	4:1	50	9.00	2.25	45.00	11.25	4:1
30f*^#^	66	G43	66	0.02	0.68	0.12	4.50	1:38	67	5.99	2.39	40.16	16.00	3:1
AVG	53.45		70.69	3.17	1.23	22.52	8.23	2.74:1	69.55	5.19	1.19	35.83	10.81	3.31:1
SD	13.74		15.57	3.21	0.70	22.77	4.37	—	14.86	3,95	1,04	26,19	6,08	—

Cases that used both THC-dominant and CBD-dominant FCEs at any point during treatment were marked with an asterisk (*). The remaining cases used either CBD-rich or THC-rich FCE. ^&^ Cases in that patients switched associations that provided FCE for treatment. # Cases where the CBD:THC ratio was modified during treatment. Notice that values in the columns expressing dosage in mg/kg/day must be multiplied by 10^−1^. Cases were identified by a number followed by the letter “f” for female. The International Classification of Diseases (ICD-11) provides standardized diagnostic codes used here to describe the specific chronic pain syndromes (CPS) diagnosed in our patient cohort.

### 2.3 Full spectrum cannabinoid extracts

Most patients reported choosing to acquire their FCEs from non-industrialized civil patients’ societies for economic reasons. To minimize sample heterogeneity, we included only patients who used FCEs from two specific patients’ societies. Thus, the cannabis extracts used by patients in this study were either produced and sold by the Brazilian Society of Medicinal Cannabis Patients (AMAME) or by the Cannabic Society in Defense of Life (Maria Flor/MALELI). Depending on the society to which the patient was affiliated, the CBD-dominant FCEs had a CBD ratio of either 4:1 or 21:1, while THC-dominant FCEs had a ratio of either 1:38 or 1:22. Information on particular oils used and their cannabinoid proportions per patient can be found on Supplementary Material S1. The quality and cannabinoid concentrations of the FCEs were verified by independent laboratory analysis using high-performance liquid chromatography (HPLC). Thus, composition information was provided by the associations, along with documentation from these certified independent laboratories containing quality and composition analyses of samples from the different formulations used (Supplementary Materials S4, S5). Full CBD and THC concentrations from those certificates were used as values for calculation of each patient’s extract regimen.

### 2.4 Patients’ Self-Reported Outcome Survey

We employed a structured, patient-reported, cross-sectional retrospective outcome survey sent by e-mail to the patients or their families (Supplementary Material S2). The survey structure was adapted from the epidemiological methodology previously used by Fleury and colleagues (Fleury-Teixeira et al., 2019). The survey consisted of multiple-choice questions about the perceived effects of FCE treatment in several symptoms and burden-related categories, in which we have employed a Likert-like scale (Joshi et al., 2015). Each question presented six possible answers: “Does Not Apply”, “Considerable worsening”, “Moderate worsening”, “No change”, “Moderate improvement”, and “Considerable improvement”. Apart from the “Do not apply” answer, numeric labels (-2, −1, 0, 1 and 2) were assigned to each answer.

It has been shown, by qualitative content analysis, that not all aspects considered important by chronic pain patients to their function and wellbeing are properly evaluated by standard questionnaires commonly used to assess the impact of chronic pain in their lives (Calmon Almeida et al., 2020). Calmon Almeida et al. (2020) results indicated that the patient’s perception of their physical capacity and ability to perform daily activities, as well as emotional and contextual factors, should be included in clinical assessments to fully understand chronic pain patient´s needs. Therefore, we have included in our Patients’ Self-Reported Outcome Survey questions concerning a wide range of core and comorbidities symptoms of CPS, as well as practical aspects to evaluate the effects of FCE treatment on both clinical and personal life burdens. We also included two multiple choice and two open-ended questions concerning the overall quality of life of the patients and their families, respectively. These later questions were analysed by Qualitative Content Analysis (see in the next section).

The survey inquired about the perceived FCE treatment outcome on 9 symptom categories and 6 additional aspects, as follows:

1. Pain episodes (PE);
2. Sleep issues (SI);
3. Persistent feeling of fatigue (PFF);
4. Motor impairments (MI);
5. Memory and cognitive problems (MCP);
6. Decreased libido or other sexual disfunctions (DLSD);
7. Sadness, melancholy, and prostration (SMP);
8. Distress and irritability (DI);
9. Anxiety crisis and/or panic attacks (AC/PA);
10. Inability to maintain a professional occupation (IMPO);
11. Negative impacts on social and family relations (NIOR);
12. Overall quality of life of the patient (QoLP);
13. Overall quality of life of the family (QoLF);
14. Side effects due to FCE treatment;
15. Use of other medications.

These questions refer specifically to the effects observed after FCE inclusion in the patient’s treatment scheme. Most, but not all, patients were already using other medications to treat chronic pain before the inclusion of FCE. Data concerning side effects and use of other medications (categories 14 and 15) were obtained by a different type of multiple-choice questionnaire (see Supplementary Material S2) and complemented by information from the patients’ clinical files.

#### 2.4.1 Qualitative content analysis

In addition to the multiple-choice questions described above, responders were asked in two separated open-ended queries to freely describe in their own words how their quality of life, and the quality of life of their family, was before and after FCE treatment. The answers for these queries were submitted to qualitative content analysis, as previously described (Hsieh and Shannon, 2005; Fontanella et al., 2006; Campos and Turato, 2009; Assarroudi et al., 2018; Faria-Schutzer et al., 2021; Grove et al., 2023). In line with the multiple-choice questions, these open-ended questions refer specifically to the effects observed after FCE inclusion in their treatment scheme. These open-ended questions were included to better assess practical and subjective aspects that might be overlooked by the other structured, multiple-choice questions, so that we can better understand the patient’s perception of wellbeing and functioning before and after FCE inclusion in their treatment.

### 2.5 Data analysis

#### 2.5.1 Data analysis of the multiple-choice questions

**FIGURE 2:**
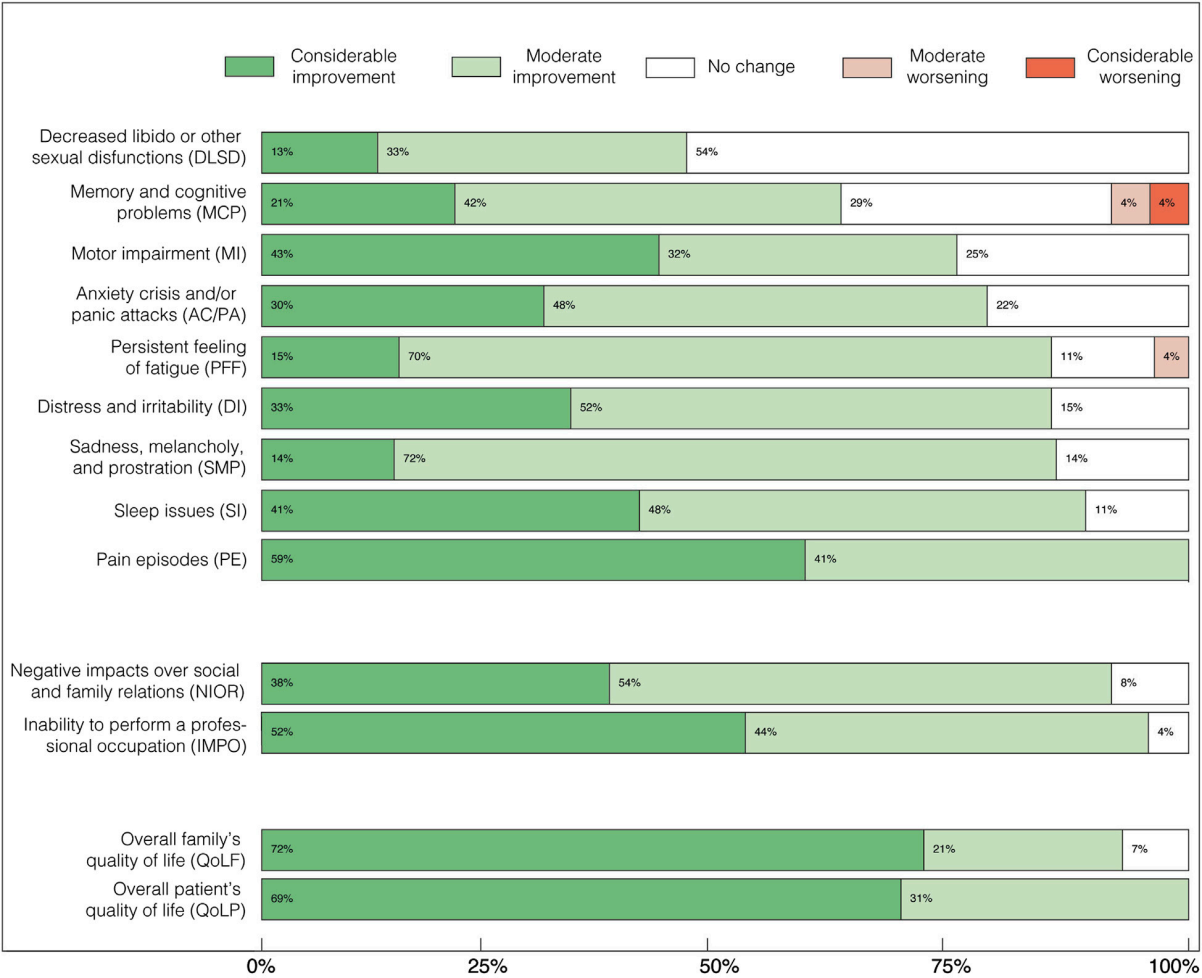
Perceived effects of FCE treatment on symptomatic categories (above), personal burden (middle), and quality-of-life (below) aspects of chronic pain syndromes, as percentages of the cohort. Above: “Decreased libido or other sexual disfunctions” (DLSD); “Memory and cognitive problems” (MCP); “Motor impairment” (MI); “Distress and irritability” (DI); “Anxiety crisis and/or panic attacks” (AC/PA); “Sadness, melancholy, and prostration” (SMP); “Persistent feeling of fatigue” (PFF); “Sleep issues” (SI); and “Pain episodes” (PE). Middle: “Negative impacts over social and family relations” (NIOR), and “Inability to perform a professional occupation” (IMPO). Below: Overall quality of life of the patient (QoLP); Overall quality of life of the family (QoLF). By the time the survey was answered, all patients were either using only conventional drugs for pain, or both conventional drugs and FCE, or only FCE. This indicates that while symptom control had improved, patients still required some form of pharmacological treatment.

Numeric labels were assigned to each of the six possible answers seen in Patient’s Survey to allow interpatient comparisons and descriptive statistics. Namely, −2; −1; 0; 1; 2 and # corresponded respectively to each of the five answers cited before, and “do not apply” (#), respectivelly (see Patients’ Self-Reported Outcome Survey). Total amount of patients for each symptom or aspect is equal to our cohort number minus the number of people who answered “Do not apply” (29 – #). Numbers in Figure 2 are shown as percentages of the whole cohort that presented each symptom or non-symptomatic aspect. A General Outcome Score (GOS) was obtained by averaging the results for the symptomatic categories from 1 to 9, so that the score includes only the symptoms presented by each patient, and each symptom category has the same weight.

#### 2.5.2 Data analysis of the open-ended questions

We used conventional Qualitative Content Analysis (QCA) (Bardin, 2004; Hsieh and Shannon, 2005; Faria-Schutzer et al., 2021) to analyse the two open-ended questions present in our Patients’ Self-Reported Outcome Survey. The QCA process involves identifying and categorizing patterns, themes, and meanings within the data and making inferences based on these patterns. Conventional QCA for open-ended questions involves a systematic approach to analysing responses, following steps that include data preparation, reduction, categorization, and interpretation. In our study, we focused on two questions concerning quality of life, primarily analysing respondents’ answers to these open-ended questions while consulting other survey data as needed to clarify ambiguities.

Our Patients’ Self-Reported Outcome Survey required respondents to complete all multiple-choice questions related to the effects of FCE treatment across symptomatic and personal burden-related categories before answering multiple-choice and open-ended questions about quality of life. The two multiple-choice questions on quality of life were: (a) “What is your general perception of the treatment’s effect on the patient’s overall quality of life?” and (b) “What is your general perception of the treatment’s effect on the family’s overall quality of life?” Each was accompanied by an open-ended question inviting detailed descriptions: (a) “Describe in detail, in your own words, the patient’s overall quality of life before and after treatment,” and (b) “Describe in detail, in your own words, the family’s overall quality of life before and after treatment.” This structure encouraged respondents to reflect thoroughly on symptoms, comorbidities, and personal burdens related to CPS before providing open-ended answers on quality of life pre- and post-FCE treatment. This design intended to ensure that responses were well-informed, thoughtful, and normalized, on these aspects, across the sample.

In our study, the conventional QCA approach was adapted and conducted by CNB and RM-L, following previously established methods (Bardin, 2004; Hsieh and Shannon, 2005; Graneheim et al., 2017; Lim et al., 2017; Urech et al., 2019; Greenfield et al., 2020; Faria-Schutzer et al., 2021). The process involved data preparation and reduction (summarization) and independent compilation of emergent categories based on researchers’ reading of the responses. Words and expressions with closely related meanings were grouped into categories, which were defined solely by participant language. A consensus between researchers was then reached to create a general categorization, organized by meaning units as described by Bardin (2004). The analysis was divided into two parts, examining the situation before and after FCE treatment for each question (one regarding the patient’s quality of life, the other regarding the family’s quality of life). In some cases, the categories found in the “before” responses had no counterpart in the “after” responses, as both sets of categories were derived directly from responders’ freely expressed answers. The frequency of each category was expressed as a percentage.

All data collected from multiple-choice questions and qualitative content analysis were plotted using MATLAB^®^ R2022a.

## 3 Results

### 3.1 General results

Invitations were sent to 178 (53 males and 125 females) potential participants and 48 of them agreed to take part in the research by answering our survey and giving their written consent for their chart’s information. Following the exclusion criteria described in Methods, the final cohort included a total of 29 CPS patients aged 27 to 84 (only females).

The participants were randomly assigned numeric labels followed by a letter, “f” for female (see methods for further explanation regarding the ID system). Details on all participants can be found in Table 1. ICD descriptors included under “Chronic Pain Syndromes” were M16, M25.5, M53.3, M54, M54.2, M54.4, M54.5, M79.1, M79.6, M79.7, M79.9, M89, G43 and G54, being defined as such by the physician according to clinical criteria. Six people interrupted treatment with FCE of their own accord: three of them had financial restrictions (6f, 15f and 28f), two of them felt pain had decreased enough to suspend FCE use (26f and 29f), and one stopped after medical recommendation due to concomitant treatment for bipolar disorder (20f). Thus, treatment duration ranged from 1 to 44 months (13 months, on average). The average weight of the cohort slightly decreased as cannabinoid concentrations used for treatment increased in comparison to initial proportions (Table 1). In only one case, 1f, the survey was answered by a third person. In Table 2 we provide the raw data from the survey answers of all participants about their perceived effects of FCE treatment. Responses were marked as a hashtag in cases when a given symptom/aspect did not apply to that patient. We have separated the 13 main categories addressed by multiple-choice questions in two subgroups: “Perceived symptomatic effects” and “Perceived personal life effects” (Table 2). Data about side effects and use of other medication are shown in Tables 7, 8.

**TABLE 2:** Perceived effects on symptoms and non-symptomatic aspects of CPS after FCE treatment for each patient. Pain episodes (PE, n = 29); Sleep issues (SI, n = 28); Persistent feeling of fatigue (PFF, n = 28); Motor impairment (MI, n = 29); Memory and cognitive problems (MCP, n = 25); Decreased libido or other sexual disfunctions (DLSD, n = 26); Sadness, melancholy and prostration (SMP, n = 29); Distress and irritability (DI, n = 28); Anxiety crisis and/or panic attacks (AC/PA, n = 24); General Outcome Score (GOS); Negative impacts over social and family relations (NIOR, n = 24); Inability to maintain a professional occupation (IMPO, n = 22) Overall quality of life of patient (QoLP, n = 29); Overall quality of life of family (QoLF, n = 29).

Case	Duration of FCE treatment (months)	Perceived symptomatic effects										Perceived personal life effects			
												Personal burden		Life quality	
		Pain Episodes	Sleep Issues	Persistent Feeling of Fatigue	Motor Impairment	Memory and Cognitive Problems	Decreased Libido or other Sexual Disfunctions	Sadness Melancholy and Prostration	Distress and Irritability	Anxiety Crisis and/or Panic Attacks	General Outcome Score	Negative Impact Over Relationships	Inability to Maintain a Professional Occupation	Patient's Quality of Life	Family's Quality of Life
1f	10	2	2	1	2	0	#	1	#	#	1.3	0	#	2	2
2f*	23	1	1	1	1	1	0	0	0	1	0.7	1	1	1	1
3f	7	1	1	1	1	#	1	1	1	1	1	1	#	1	1
4f	1	2	2	2	2	2	1	1	1	2	1.7	#	2	2	2
5f	7	1	1	1	1	1	0	1	1	1	0.9	1	2	2	2
6f	26	2	2	1	2	1	0	1	2	2	1.4	2	2	2	2
7f	20	2	2	1	2	1	2	2	2	2	1.8	1	2	2	2
8f	6	2	1	1	2	1	0	1	0	0	0.9	1	1	2	2
9f*	19	1	1	1	0	-2	1	1	1	0	0.4	1	#	1	1
10f	25	2	1	1	2	0	0	1	2	0	1	2	1	2	2
11f	11	1	1	1	1	0	1	1	1	#	0.9	0	1	1	0
12f	4	1	1	1	1	#	0	0	0	0	0.5	0	1	1	1
13f	2	2	2	2	2	1	0	1	0	1	1.2	#	#	2	2
14f	4	1	0	1	1	0	0	2	2	2	1	1	1	1	1
15f	3	2	1	1	2	2	2	1	1	2	1.6	2	2	2	2
16f*	9	2	2	2	2	0	1	2	2	1	1.6	2	2	2	2
17f	6	1	0	1	0	1	#	1	1	1	0.8	#	0	1	1
18f*	26	2	2	2	2	1	0	1	2	2	1.6	2	#	2	2
19f*	4	1	1	0	1	1	1	1	1	1	0.9	1	1	2	2
20f*	7	2	1	1	1	1	1	1	1	1	1.1	1	1	1	0
21f*	44	2	2	-1	0	-1	2	1	1	0	0.7	1	2	2	2
22f	14	1	2	1	0	0	0	1	2	#	0.9	2	1	2	2
23f	13	2	2	2	2	2	#	#	#	#	2	#	2	2	2
25f	13	1	#	#	2	#	0	0	0	#	0.6	#	#	2	2
26f	3	2	0	0	0	#	0	0	0	1	0.4	2	2	2	2
27f	36	2	2	1	1	2	1	1	2	1	1.4	1	2	2	2
28f	4	2	2	1	0	2	0	2	2	2	1.4	2	1	1	2
29f	5	2	#	#	#	#	#	1	1	1	1.3	#	2	2	#
30f*	13	1	1	0	0	0	0	0	1	#	0.4	1	#	1	1
**N**	29	29	27	27	28	24	25	28	27	23	29	23	22	29	28
Mean	12.59	1.59	1.33	1.00	1.18	0.71	0.56	0.96	1.11	1.09	1.08	1.22	1.45	1.66	1.61
SD	10.57	0.49	0.67	0.67	0.80	0.98	0.70	0.57	0.74	0.72	0.43	0.66	0.58	0.48	0.62

Outcome scale used: # “Does Not Apply”, −2 “Considerable Worsening”, −1 “Moderate Worsening”, 0 “No Change”, 1 “Moderate Improvement” and 2 “Considerable Improvement”. Cases that used both CBD-rich and THC-rich FCEs at some time during treatment were marked with an asterisk (*). Cases were identified by a number followed by the letter “f” for female. By the time the survey was answered, all patients were either using only conventional drugs for pain, or both conventional drugs and FCE, or only FCE. This indicates that while symptom control had improved, patients still required some form of pharmacological treatment.

### 3.2 Perceived symptomatic effects

Patients reported expressive improvement across all evaluated categories after FCE was included in their treatment scheme as an adjuvant or the only pharmaceutical treatment. We highlight that all patients reported alleviation on the intensity of “Pain episodes” (Figure 2), and most of them also reported subsequent/concomitant improvements on comorbid symptoms, as well as on personal life-related aspects (Table 2; Figure 2). Some level of improvement was reputed present whenever “Moderate improvement” (Mi) and/or “Considerable improvement” (Ci) was different from zero (Figure 2). “Moderate improvement” was the most frequent perceived outcome for most symptomatic categories: “Decreased libido or other sexual disfunctions” (Mi: 33%, Ci: 13%); “Memory and cognitive problems” (Mi: 42%, Ci: 21%); “Motor impairment” (Mi: 32%, Ci: 43%); “Distress and irritability” (Mi: 52%, Ci: 33%); “Anxiety crisis and/or panic attacks” (Mi: 48%, Ci: 30%); “Sadness, melancholy, and prostration” (Mi: 72%, Ci: 14%); “Persistent feeling of fatigue” (Mi: 70%, Ci: 15%); “Sleep issues” (Mi: 48%, Ci: 41%); and “Pain episodes” (Mi: 41%, Ci: 59%). Thus, in 8 out of 10 symptomatic categories 70%–90% of the patients perceived some level of improvement after FCE treatment. Only two categories, “Reduction of libido” and “Memory and cognitive deficits”, showed less frequent improvements, so that some improvement was perceived by 46% and 63% of the patients, respectively. The category “Memory and cognitive deficits” corresponded to most cases of worsening symptoms (8%). In two categories, “Pain episodes” and “Motor impairments”, the most frequent outcome was “Considerable improvement”. In only one category, “Decreased libido”, “No change” was the most frequent perceived outcome.

### 3.3 Perceived personal life effects

The subgroup of personal life-related categories presented a large percentage of some improvement and no cases of worsening (Figure 2): “Negative impacts over social and family relations” (Mi: 54%, Ci: 38%), “Inability to perform a professional occupation” (Mi: 44%, Ci: 52%); Overall quality of life of the patient (Mi: 21%, Ci: 72%); Overall quality of life of the family (Mi: 31%, Ci: 69%).

### 3.4 Qualitative content analysis of life quality open-ended questions

The responses to the two open-ended questions exploring the impact of FCE treatment on quality of life, initially provided in Brazilian Portuguese, underwent meticulous translation into English. The comprehensive translations are presented in their entirety in Supplementary Material S3.

We employed qualitative content analysis to delve into these open-ended responses, aiming to uncover the subjective and practical implications of CPS and assess the impact of cannabis-based treatment on both patients and their families’ quality of life. While the Patient-reported Outcome Survey comprises structured, multiple-choice inquiries covering predetermined clinical, functional, and personal dimensions, our focus here is on the unscripted insights spontaneously shared by patients. The qualitative content analysis allows us to gain a comprehensive understanding, directly from the patients’ perspectives, regarding the effects of both CPS and cannabis-based treatment on their own quality of life and that of their families. The data collected is devoid of predefined parameters, offering a holistic view that encapsulates the nuanced experiences and challenges faced by patients undergoing CPS and FCE treatment.

#### 3.4.1 Patients’ quality of life

The open-ended query, “Describe in detail, in your own words, what the patient’s overall quality of life was like before the treatment and what it came to be like after the treatment,” garnered responses from all 29 patients (see Supplementary Material S3 for complete textual answers). Each respondent, in varying degrees, conveyed an improvement in their quality of life following the treatment. While two patients reported positive changes but had to discontinue the treatment, three others affirmed an enhancement without specifying the nature of the improvement. Two patients did not explicitly address improvement in quality of life in the open question; however, in the corresponding multiple-choice question, they selected “there was moderate improvement” and “there was considerable improvement,” respectively.

A meticulous analysis of each response was undertaken to interpret its semantic content. Individual words or expressions were treated as distinct meaning units, which were then categorized based on related themes (Tables 3, 4). Distinct categories emerged as responses of all patients were scrutinized, forming a comprehensive picture of the patients’ experiences before and after FCE treatment.

**TABLE 3:** Patient’s perceived quality of life before FCE Treatment.

Category (representative examples of the respective meaning unities)	Frequency^a^
Constant pain	20
Psychological distress subcategories [frequency] Anxiety [5] (fear; panic; worry) Melancholy [3] (sadness; depression; absence of pleasure) Lack of motivation [3] (lack of energy; lethargy; apathy) Irritability [2] Lack of libido [1]	14
Limitations for daily activities (difficulty performing household tasks, self-care activities, physical exercise, work, and study)	8
Motor limitations (loss of strength; joint stiffness; feeling “locked up”; loss of certain movements; difficulty standing to take a shower)	7
Sleep issues (insomnia; unsatisfactory sleep; insufficient deep sleep; awakenings during the night)	6
Excessive medications (use of many medications for pain and comorbidities)	3
Lack of social or leisure activities (limitations for playing with children, going out with family, traveling, and participating in events or social activities)	3
Cognitive difficulties (difficulties with memory, attention, concentration, organization of ideas; mental confusion)	2
Others (fatigue; cramps; swelling in the face; blurred vision; sensation of shock in the neck; numbness; adverse effect of the drug on intestinal function)	2^b^
Total	45

^a^ Number of patients that mentioned the meaning unites grouped into a category.

^b^ Some patients mentioned more than one meaning unit included into the “Others” category.

**TABLE 4:** Patient’s perceived quality of life after FCE treatment.

Category (representative examples of the respective meaning unities)	Frequency^a^
Pain relief (pain reduction or elimination)	14
Improvement of daily activities (more energy for daily activities, going out more; performing household tasks; exercising more; return to studying)	12
Reduction of psychological distress (greater tranquillity; calmness; more tolerance; reduction of anxiety; reduction of depression, sadness and irritability; mental relaxation)	8
Less medications (reduction or elimination of other medications)	6
Better Sleep (improvement on the quality of sleep)	5
Enjoyment of life (joy of living; pleasure; happiness)	4
Cognitive improvement (improved memory and concentration; better studying and remembering things)	3
More social or leisure activities (talking more to people; interacting more; more involved in social and leisure activities)	3
Others (more autonomy; muscle relaxation; reduction of hospitalization; return of motor skills, elimination of the adverse effect of the drug on intestinal function)	3^b^
Total	55

^a^ Number of patients that mentioned the meaning unites grouped into a category.

^b^ Some patients mentioned more than one meaning unit included into the “Others” category.

Consider the following actual individual response as an illustration:

“*Before: a lot of pain, motor limitation, discouragement, anguish. After: wellbeing, return of motor skills”.*

In this instance, four meaning units were identified for the period before treatment (“a lot of pain”, “motor limitation”, “discouragement” and “anguish”), while two meaning units pertained to the post-treatment phase (“wellbeing” and “return of motor skills”). For consistency, synonyms, or related terms, such as “motor impairment,” used by other patients, were grouped under the same meaning category, “Motor limitations” in the Table 3. To ensure representativeness, any meaning unit with a frequency of only one—mentioned by a sole patient with no similar mentions by others—was categorized as “Others.” Consequently, the “return of motor skills” was grouped under the “Others” category in the post-treatment phase (see Table 4), since nobody else explicitly mentioned anything correlated to this in the “after” part of their answers. This kind of situation may dilute information that is important to a particular case, but less significant for the whole sample, nevertheless, a more focused individual appreciation of the responses can be found in the complete textual answers present in the Supplementary Material S3.

The frequency of each meaning category was tallied for both the periods before and after FCE treatment, as responses from all patients were collected. A notable aspect of this analytical approach is its reliance on the patients’ responses, independent of any influence from the researchers. Consequently, distinct sets of categories emerged for the “Before” and “After” periods, shaped exclusively by the patients’ subjective experiences. Notably, most categories have counterparts in both groups, while some may be unique to either the “Before” or “After” set. In the aforementioned example, only the meaning unit ‘motor limitation’ from the ‘Before’ set of this patient’s responses had a corresponding counterpart in the ‘After’ set, namely, ‘improvement of motor limitations.’ However, the meaning unit ‘return of motor skills’ was included in the grouped ‘Others’ category because only this patient mentioned it in the after-treatment phase (Table 4).

The analysis of open answers offers valuable insights into patients’ subjective perceptions, elucidating the nuanced significance they attribute to different aspects of their lives before and after treatment. It underscores the idea that certain aspects may gain prominence in retrospective reflections on the pre-treatment period, while post-treatment considerations may highlight consequences that were not previously given the same attention. Thus, this analytical approach enabled an exploration of the most personally impactful aspects related to CPS, when most patients were using other medications for pain and comorbidities, but no patient was using FCE. And, provided a nuanced understanding of the consequential effects of incorporating FCE into their treatment regimen.

**FIGURE 3:**
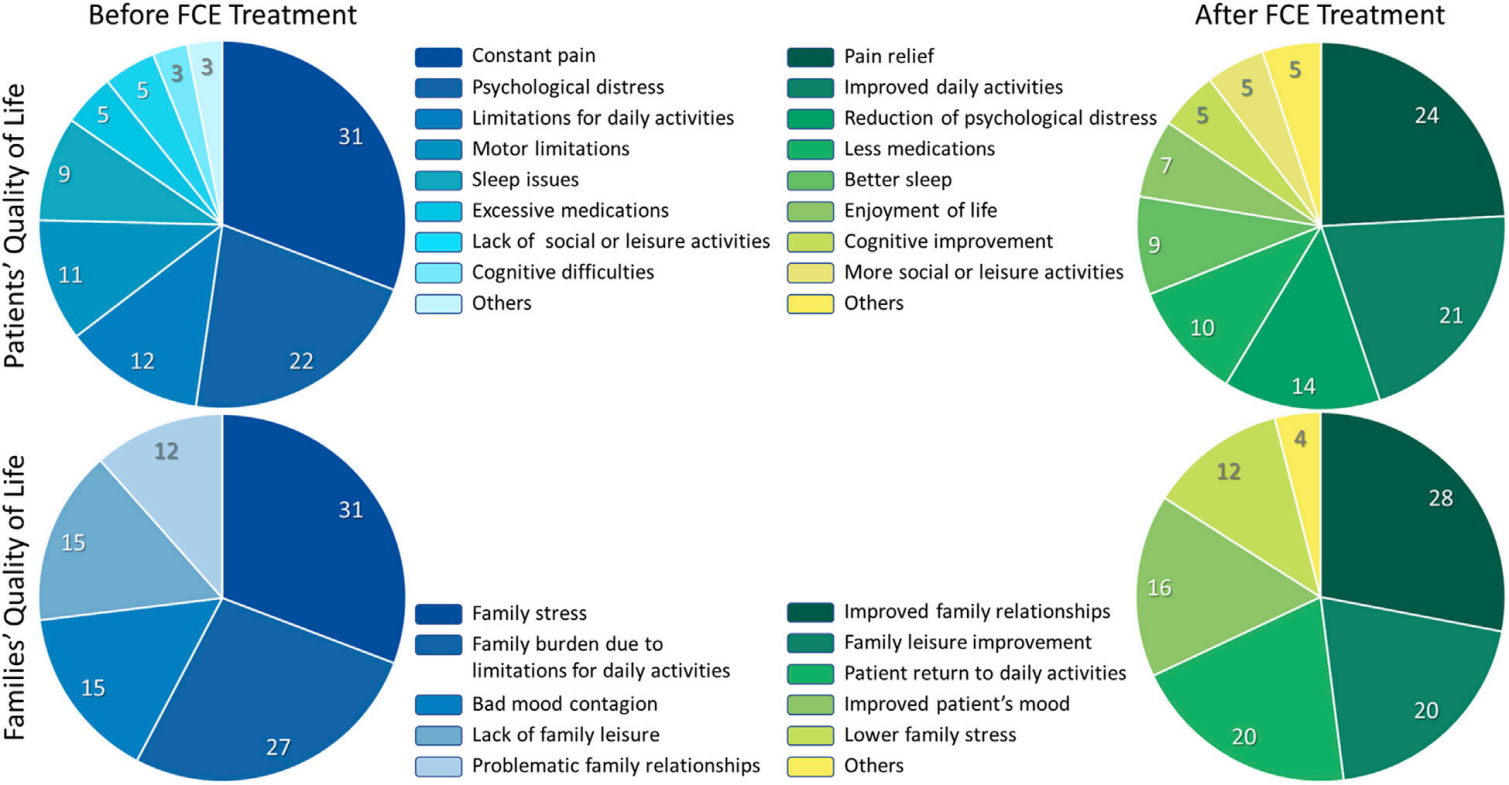
Qualitative Content Analysis of the open-ended answers. Two open-ended questions concerning quality of life were included at the end of our Patient Self-Reported Outcome survey. The content of the answers was categorized as described in the methods. Here we show the relative frequency of each category in four instances: Perceived situation concerning the patients’ quality of life before FCE treatment. Perceived situation concerning the patients’ quality of life after FCE treatment. Perceived situation concerning the families’ quality of life before FCE treatment. Perceived situation concerning the families’ quality of life after FCE treatment. The percentages refer to the frequency with which a given category was mentioned relative to the total number of mentions shared by all categories. At the time the survey was completed, all patients were using either only conventional drugs for pain, a combination of conventional drugs and FCE, or FCE alone. This indicates that, although symptom control was improved for all patients (see Figure 2) and was also reflected in open-ended responses regarding quality of life after FCE, all participants continued to require some form of pharmacological treatment.

Regarding the “before FCE treatment” situation, 9 main meaning categories have emerged from the answers (Table 3): “Constant pain” (frequency of 20, representing 30.77% of the total), Psychological distress (14, 21.54%), “Limitation for daily activities” (8, 12.31%), “Motor limitations” (7, 10.77%), Sleep issues” (6, 9.23%), “Excessive medications” (3, 4.62%), “Lack of social or leisure activities (3, 4.62%), “Cognitive difficulties” (2, 3.08%), “Others” (2, 3.08%). The category “Psychological distress’ was composed of 6 subcategories, “Anxiety” (5, 7.69%), “Melancholy (3, 4.62%), “Lack of motivation” (3, 4.62%), “Irritability” (2, 3.08%), and “Lack of libido” (1, 1.54%). Regarding the “after FCE treatment” situation, 9 meaning categories have emerged from the answers (Table 4): “Pain relief” (14, 24.14%), “Improved daily activities” (12, 20.69%), “Reduction of psychological distress” (8, 13.79%), “Less medication” (6, 10.34%), “Better sleep” (5, 8.62%), “Enjoyment of life” (4, 6.90%), “Cognitive improvement” (3, 5.17%), “More social or leisure activities” (3, 5.17%), “Others” (3, 5.17%). The QCA results regarding patients’ quality of life on both, “before FCE treatment” and “After FCE treatment” situations are represented as percentages of each category in Figure 3.

#### 3.4.2 Families’ quality of life

The open-ended query “Describe in detail, in your own words, what the family’s overall quality of life was like before the treatment and what it came to be like after the treatment.”, was answered by 26 patients (Supplementary Material S3), and 22 reported that there was an improvement in the family’s quality of life after treatment. Among those, two explained that there was an improvement in the quality of life of the family but did not specify what the improvement consisted of. Five did not explain in their answer to the open-ended question that there was an improvement in the quality of life of the family, but when answering the multiple-choice question, they pointed out the option “there was a considerable improvement”. One of these patients had to discontinue use. Four patients did not observe changes in the family’s quality of life. The answers were grouped into categories referring to the period “before FCE treatment” and “after FCE treatment”. Five categories emerged from the answers regarding families’ quality of life before FCE treatment (Table 5): “Family stress” (8, 30.77%), “Family burden due to limitations for daily activities” (7, 26.93%), “Bad mood contagion” (4, 15.38%), “Lack of family leisure” (4, 15.38%), and “Problematic family relationships” (3, 11.54%). The answers regarding families’ quality of life after FCE treatment were grouped into six categories (Table 6): “Improved family relationships” (7, 28.00%), “Family leisure improvement” (5, 20.00%), “Patient return to daily activities” (5, 20.00%), “Improvement in patient’s mood” (4, 16.00%), “Lower family stress” (3, 12.00%), “Others” (1, 4.00%). The QCA results regarding families’ quality of life on both, “before FCE treatment” and “After FCE treatment” situations are represented as percentages of each category in the Figure 3.

**TABLE 5:** Family’s perceived quality of life before FCE treatment.

Category (representative examples of the respective meaning unities)	Frequency^a^
Family stress (fears; tension; shock; worries; sleepless nights; family suffering together; need for hospitalization)	8
Family burden due to limitation for daily activity (patient’s limitations for daily activities; locomotion problems; difficulties for performing household tasks; incapacity for working in a job; impaired working performance; increased need for family assistance)	7
Bad mood contagion (patient feels discouraged, anxious, depressed, stressed, impatient and/or indisposed, leading to bitterness that affects the whole family)	4
Lack of family leisure (limitation or absence of playful activities with the family; incapacity, or lack of motivation for going out or travelling)	4
Problematic family interpersonal relationships (avoidance of hugs and sexual contact; frustration with family members because they do not completely understand the patient’s feelings; stressful conviviality)	3
Total	26

^a^ Number of patients that mentioned the meaning unites grouped into a category.

**TABLE 6:** Family’s perceived quality of life after FCE treatment.

Category (representative examples of the respective meaning unities)	Frequency^a^
Improved family relations (greater socialization; willingness to talk and interact; improvement in emotional life; healthier and happier coexistence)	7
Family leisure improvement (return of the patient to playful activities with the family)	5
Patient return to daily activities (patient’s return to important daily activities, including household tasks, and helping kids with their homework)	5
Improved patient´s mood (reduction of anxiety or depression; more joy; greater wellbeing)	4
Lower family stress (more tranquillity and happiness; less worry)	3
Others (the treatment impact on family´s quality of life is perceived as a miracle)	1
Total	25

^a^ Number of patients that mentioned the meaning unites grouped into a category.

### 3.5 Other medications and side effects observed during FCE treatment

Although patients in our survey reported experiencing a range of side effects (see Table 7), these were consistently categorized as mild to moderate. Importantly, none of the patients discontinued FCE treatment due to side effects throughout the duration covered by this report. Typically, side effects were transient and predominantly manifested at the onset of treatment (see Table 8). Notably, seven patients reported no side effects during the reporting period. No distinct correlation was observed between the use of other medications and the severity of side effects reported by patients when responding to the Patient-Reported Outcome Survey. However, it is noteworthy that even the mild or moderate effects outlined in Tables 7, 8 could potentially be attributed to drug interactions. Most patients (93%) underwent treatment for 3 months or longer, suggesting stability in these results. However, it is important to acknowledge that beyond our data collection period, other potential side effects may emerge due to slower interactions with other medications. We will further address this matter in the discussion section.

**TABLE 7:** Side effects reported by patients as being associated with FCE treatment. Occurrence marked as “?” indicates that information on the intensity and/or duration of the effect was omitted in the survey, and could not be obtained from patients’ clinical files, or follow-up contacts.

Side effect	Frequency	%	Severity	Occurrence
Weight gain	1	3,45	?	?
Cough	1	3,45	Mild	Only In the beginning
Red eyes	1	3,45	Mild	Still ongoing
Excessive appetite	1	3,45	Mild	Still ongoing
Depression	1	3,45	Mild	Only In the beginning
Ataxia	1	3,45	Mild	Only In the beginning
Euphoria	2	6,90	Mild to moderate	Only In the beginning
Agitation	2	6,90	Mild	Only In the beginning
Constipation	2	6,90	Mild	Only In the beginning
Confusion	1	3,45	Mild	Only In the beginning
Blurred vision	2	6,90	Mild	In the beginning or still ongoing
Excessive thirst	3	10,34	?	?
Diarrhoea	3	10,34	Mild	Only In the beginning
Feeling “high”	3	10,34	Mild	In the beginning or still ongoing
Tachycardia	4	13,79	Mild	Only In the beginning
Headache	5	17,24	Mild to moderate	In the beginning or still ongoing
Nausea	6	20,69	Mild	In the beginning or still ongoing
Anxiety	6	20,69	Mild to moderate	In the beginning or still ongoing
Drowsiness	7	24,14	Mild to moderate	In the beginning or still ongoing
Insomnia	8	27,59	Mild to Moderate	In the beginning or still ongoing
Dizziness getting up	9	31,03	Mild to moderate	In the beginning or still ongoing
Dry mouth	12	41,38	Mild	Only In the beginning

**TABLE 8:** Medications other than FCE used by the patients, and side effects observed during FCE treatment. By the time the survey was answered, all patients were either using only conventional drugs for pain, or both conventional drugs and FCE, or only FCE. This indicates that while symptom control had improved, patients still required some form of pharmacological treatment.

Case	GOS	Medication before FCE	Medication after FCE	Summary of changes	FCE treatment side effects
1f	1.3	Duloxetine^#$^; Pregabalin^#^	Duloxetine^#^	WD of Pregabalin^#$^ and DR of Duloxetine^#$^	Moderate insomnia and dry mouth, mild “high”, nausea, euphoria, agitation, dizziness, and constipation (all only in the beginning)
2f*	0.7	Pregabalin^#$^; Quetiapine	Pregabalin^#$^; Duloxetine^#$^	WD of Quetiapine^$^, DR of Pregabalin^#&^, addition of Duloxetine^#$^	None
3f	1.0	Fluoxetine^$^; Pregabalin^#$^; Amitriptyline^#^	Fluoxetine^$^; Amitriptyline^#^	WD of Pregabalin^#$^; and DR of Amitriptyline^#^	Mild dry mouth (only in the beginning)
4f	1.7	Levetiracetam^#^; Pregabalin^#$^	None	CWD	Mild dizziness and tachycardia (only in the beginning); Mild insomnia, dry mouth, nausea, and anxiety (still ongoing)
5f	0.9	Triptan^#^; Propranolol^#^	Triptan^#^	WD of Propranolol^#^ (before starting FCE treatment)	Mild insomnia, mild dizziness, mild anxiety
6f	1.4	Duloxetine^#$^; Pregabalin^#$^	Duloxetine^#$^; Pregabalin^#$^	None	None
7f	1.8	Diclofenac^#^	None	CWD	None
8f	0.9	Tramadol^#^; Pregabalin^#$^	None	CWD	None
9f*	0.4	Duloxetine^#$^; Pregabalin^#$^	Duloxetine^#$^	WD of Pregabalin^#$^ and DR of Duloxetine^#$^	Drowsiness, dry mouth, excessive thirst, weight gain
10f	1.0	Zolpidem; Duloxetine^#$^; Pregabalin^#$^; Clonazepam^$^	Pregabalin^#$^; Cyclobenzaprine; Clonazepam^$^	WD of Zolpidem and Duloxetine^#$^; DR of Clonazepam^$^	Mild dizziness and tachycardia (more in the beginning)
11f	0.9	None	None	None	Mild dizziness, dry mouth (only in the beginning)
12f	0.5	Venlafaxine^$^; Zolpidem	Venlafaxine^$^; Zolpidem	DR of Venlafaxine^$^	Mild drowsiness, dizziness, anxiety, nausea, diarrhoea
13f	1.2	None	None	None	Moderate insomnia, headaches, dizziness, dry mouth, anxiety, constipation
14f	1.0	Duloxetine^#$^; Promethazine	Duloxetine^#$^; Promethazine	None	Mild headache and nausea, moderate insomnia, coughs, and anxiety (only in the beginning)
15f	1.6	Duloxetine^#$^; Pregabalin^#$^; Codeine^#^; Tramadol^#^; Cyclobenzaprine^#^	(Lysine Clonixinate + Cyclobenzaprine)^#^	CWD and addition of (Lysine Clonixinate + Cyclobenzaprine)^#^	Mild drowsiness (only in the beginning)
16f*	1.6	Dihydroergotamine^#^	Dihydroergotamine^#^	DR of Dihydroergotamine^#^	Moderate drowsiness (still ongoing)
17f	0.8	None	None	None	Dry mouth, excessive thirst
18f*	1.6	Venlafaxine^$^; Zolpidem; Painkillers^#^ (unspecified)	Venlafaxine^$^	WD of Zolpidem and Painkillers^#^	Mild dry mouth
19f*	0.9	Pregabalin^#$^	Pregabalin^#$^	Increased dose of Pregabalin^#$^	Mild Insomnia, headaches, dry mouth, confusion, nausea, blurred vision (only in the beginning); Mild Drowsiness, dizziness, tachycardia, red eyes, excessive appetite (still ongoing)
20f*	1.1	Duloxetine^#$^; Lisdexamphetamine	Duloxetine^#$^; Lisdexamphetamine	None	Dry mouth, excessive thirst
21f*	0.7	None	None	None	Moderate insomnia, mild headaches, dry mouth, euphoria, agitation, anxiety, “high”, depression, nausea, ataxia, tachycardia, diarrhoea, eye redness (all only in the beginning)
22f	0.9	Cyclobenzaprine^#^; Paracetamol^#^; Ketoprofen^#^; varied antidepressants^$^	Varied antidepressants^$^	WD of Cyclobenzaprine^#^, Paracetamol^#^ and Ketoprofen^#^	Mild dry mouth (only in the beginning); Mild drowsiness, headaches, and cough (all still ongoing)
23f	2.0	Alprazolam; Trazodone; Duloxetine^#$^	Duloxetine^#$^	WD of Alprazolam and Trazodone	Mild diarrhoea (only in the beginning)
25f	0.6	Venlafaxine^$^; α-Methyltryptamine^$^; Topiramate^#^; Quetiapine^$^; Clonazepam^$^	Venlafaxine^$^	WD of α-Methyltryptamine^$^, Topiramate^#^, Quetiapine^$^ and Clonazepam^$^; and DR of Venlafaxine^$^	None
26f	0.4	Triptan^#^; (Metamizol + Orphenadrine + Caffeine)^#^; Venlafaxine^$^	Triptan^#^; (Metamizol + Orphenadrine + Caffeine)^#^; Venlafaxine^$^	DR of all medications	None
27f	1.4	Cyclobenzaprine^#^; (Metamizol + Orphenadrine + Caffeine)^#^	None	CWD	Mild dizziness (only in the beginning); Mild Drowsiness and blurred vision (still ongoing)
28f	1.4	Trazodone^$^; Tramadol^#^; Duloxetine^#$^; Gabapentin^#$^	Trazodone^$^; Tramadol^#^; Duloxetine^#$^; Gabapentin^#$^	None	None
29f	1.3	Flunarizine^#^	None	CWD	Mild insomnia and “high” (still ongoing)
30f*	0.4	Amitriptyline^#$^; Lithium^$^	Lithium^$^	WD of Amitriptyline^#$^ and DR of Lithium	Mild headache (still ongoing)

Cases that used both “CBD-dominant” and “THC-dominant” FCEs at some time during treatment were marked with an asterisk (*). GOS: general outcome score; DR: dose reduction; WD: withdrawal; CWD: complete withdrawal; #: medications that may be used to control pain; $: medications that may be used to control mood disorders (depression, anxiety, or bipolar disorders). Cases were identified by a number followed by the letter “f” for female.

Most patients (83%, n = 24 out of 29) were already taking traditional analgesics prior to starting FCE treatment (refer to Table 8). After starting FCE treatment, a considerable number of these patients (66%, n = 16 out of 24) reduced or discontinued their analgesic use, while a few patients (8%, n = 2 out of 24) switched to either duloxetine or a combination of lysine clonixinate and cyclobenzaprine, and some patients (21%, n = 5 out of 24) continued with the same analgesic dose. Only one patient (19f) increased the analgesic dose. Among the patients who were not taking analgesics before FCE treatment (n = 5), none of them added any medication during the treatment period. Additionally, 20 (69%) of all patients were taking medication for anxiety and/or mood disorders (see Table 8), and out of those, 10 (50%) patients reduced or completely stopped taking their medication after FCE treatment. One patient (2f) discontinued quetiapine, reduced pregabalin, and added duloxetine, while only one patient (19f) increased Pregabalin dosage. The remaining eight patients continued with the same dosage of their medication for anxiety and/or mood disorders.

## 4 Discussion

### 4.1 Main results

The individually tailored FCE dosage scheme reported here presented tangible and significant benefits across all the CPS symptomatic categories in the presented cohort (14 different ICD descriptors). Our data were drawn from medical records and a comprehensive patient survey combining multiple-choice and open-ended questions. The open-ended responses were analysed using QCA, a method widely applied to clinical data—such as narratives, medical records, and interviews—to explore patient experiences and assess treatment impacts (Bardin, 2004; Fontanella et al., 2006; Lim et al., 2017; Urech et al., 2019; Greenfield et al., 2020; Faria-Schutzer et al., 2021; McKeon et al., 2021; Mattila-Rautiainen et al., 2023). QCA has been especially useful in chronic illness research (Dehghani et al., 2019; Calmon Almeida et al., 2020; Abaah et al., 2023; Lundin et al., 2023) by offering deeper insight into patients’ subjective perceptions and attitudes toward care (Pope and Mays, 1995; Fontanella et al., 2006; Campos and Turato, 2009; Schwieger-Briel et al., 2015; Lim et al., 2017; Calmon Almeida et al., 2020; Faria-Schutzer et al., 2021). In our study, QCA revealed that FCE treatment positively impacted patients’ practical and emotional lives, with benefits often extending to family members—even among those already using conventional medications.

Our exploratory, real-life approach provided a broader understanding of the potential benefits of FCEs across diverse CPS conditions (see Supplementary Material S1). Although mild to moderate side effects were reported, none were mentioned in the open-ended responses.

Chronic pain, the core CPS symptom, was alleviated to some extent in all 29 patients: 60% reported considerable relief, and 40% moderate relief (Figure 2). While placebo effects cannot be ruled out in this open-label study, several factors support a pharmacologically mediated therapeutic benefit. At the time of the survey, most patients (69%) had used FCEs for 6 months or longer (Table 1), and 83% had been on analgesics prior to FCE treatment (Table 8). Among these, 16 (66%) discontinued or reduced use and only one increased dosage. None of the five patients who were not previously on analgesics started new medications during treatment. These findings suggest the reported pain relief is unlikely to be solely due to placebo.

Psychological distress was the second most frequent symptom group. After FCE treatment, most patients reported improvements in categories such as cognitive problems (64%), distress/irritability (78%), anxiety (79%), melancholy (83%), fatigue (85%), and sleep disturbances (89%) (Figure 2). At baseline, 70% were already on mood or anxiety medications; half of them reduced or discontinued use post-FCE (Table 8). Only one patient increased medication dosage. Together, these results reinforce that the benefits perceived were probably not merely placebo-driven, but accompanied by tangible reductions in pharmacological burden and improvements in wellbeing. See Section 4.3 (Limitations) for a deeper discussion about placebo effect and other potential sources of response bias.

### 4.2 Pharmacological considerations

Our findings are in line with the scientific literature, both basic and clinical, that demonstrate an interconnected combination of positive effects of cannabinoids on both the pain itself and the comorbid psychological aspects typically observed in CPS. For instance, cannabis is known to have significant effects on the affective and higher-order perceptual aspects of pain (La Porta et al., 2015; Racz et al., 2015; Bajic et al., 2018; Mecca et al., 2021; van den Hoogen et al., 2021) due to the distribution of cannabinoid receptors in frontal-limbic areas such as the amygdala, raphe, and anterior cingulate cortex, all of which are involved in pain perception (Lee et al., 2013; Weizman et al., 2018). Alterations in the medial prefrontal cortex endocannabinoid signalling have been shown to be an important contributing factor to depression following persistent neuropathic pain in rats (Mecca et al., 2021). Constant, unrelenting pain can lead to anxious and/or depressive states (Kao et al., 2021; Meda et al., 2022; Nowinka et al., 2022; Rogers et al., 2022) and can prevent CPS patients from engaging in everyday activities, including socialization (Di Tella et al., 2015; Grabli et al., 2021). Chronic pain can also impact cognitive processes such as memory and attention (Moriarty et al., 2011; Phelps et al., 2021), leading to increases in polypharmacy to manage not only pain but also secondary problems arising from the main disorder. Indeed, a significant portion of medication expenses are allocated to addressing these issues (Kronborg et al., 2009; Phillips, 2009), which could be reduced with the introduction of FCEs as a treatment option (Bellnier et al., 2018; Erku et al., 2021).

It is important to notice, however, that cannabinoids exert a significant inhibitory effect on hepatic metabolic enzymes responsible for metabolizing various pharmaceutical drugs (Bornheim et al., 1993; Yamaori et al., 2011; Jiang et al., 2013; Yamaori et al., 2014; Engeleit et al., 2021; Vaughn et al., 2021), including certain antidepressants, potentially intensifying their effects (Anderson et al., 2021). This can occasionally lead to adverse effects (Nanan et al., 2022). Cannabidiol, specifically, has demonstrated less pronounced effects on the metabolism of sertraline, fluoxetine, and mirtazapine *in vitro*, but it significantly affects the metabolism of citalopram in humans, resulting in increased plasma concentrations (Anderson et al., 2021). While we have not observed any adverse effects clearly linked to this interaction, we cannot rule out the possibility that some side effects and/or the observed psychological improvements may be partially attributable to the influence of cannabinoids on antidepressant metabolism.

Cannabinoid signalling through either CB1 or CB2 receptors is central to the analgesic effect of cannabinoids on inflammatory pain (Zhang et al., 2003; Valenzano et al., 2005; Pernia-Andrade et al., 2009; Berger et al., 2014; Krustev et al., 2014; Zhang et al., 2014; Xu et al., 2016; Thompson et al., 2020; Park and Watkins, 2021; Bogdan et al., 2022). Accordingly, both THC and CBD has been shown to be effective in reducing persistent inflammatory or neuropathic pain in rodents (Britch et al., 2020; Mitchell et al., 2021). In further agreement with our results, studies have demonstrated that a combination of CBD and THC in FCE may be more effective for treating chronic pain (Longo et al., 2021), and a growing number of evidences support the use of cannabis-based medicines to treat psychological, social and functional aspects of pain and CPS-associated comorbidities (Jones et al., 1981; Pertwee, 1999; Aggarwal et al., 2009; Avello et al., 2017; Kosiba et al., 2019; Lake et al., 2020; Li et al., 2020; Stith et al., 2020; Feinstein et al., 2021; Gruber et al., 2021; Khurshid et al., 2021; Aebischer et al., 2022; Berger et al., 2022; Kuhathasan et al., 2022; Leung et al., 2022; Sachedina et al., 2022).

It is also important to underscore the significance of the individualized approach employed here in defining the FCE dosage scheme in each patient. Contemporary medicine is exploring the benefits of a more personalized approach that considers various aspects of a patient’s medical history, such as genetic profiles and comorbidities (Cortese, 2007; Cilli et al., 2022). This is particularly important in chronic conditions, where idiosyncrasies necessitate regular dose adjustments to optimize treatment progression and adherence (Cilli et al., 2022). Because the endocannabinoid system regulates both central and peripheral pain-related circuits, and cannabinoids can block spinal, peripheral and gastrointestinal mechanisms underlying pain, it has been proposed, with growing data support, that some pain-inducing conditions may be related to congenital or acquired reduction of endocannabinoid signalling tone (Russo, 2004; Smith and Wagner, 2014; Russo, 2016), which would concur to individual variability in the tonic state of cannabinoid receptors and endocannabinoid-metabolizing enzymes. Furthermore, since it has been shown that there is important genetic variability in the metabolization of phytocannabinoids (Hryhorowicz et al., 2018), and in the individual susceptibility to the effects of phytocannabinoids over drug-metabolizing enzymes (Bardhi et al., 2022), both pharmacokinetics and drug-drug interactions of phytocannabinoids are likely to vary significantly among individuals. All these considerations are of particular importance when considering cannabis use for treating various CPS conditions in female patients, since cannabinoid pharmacology related to pain exhibits sex-specific differences (Fattore and Fratta, 2010; Blanton et al., 2021; Santoro et al., 2021). Notably, pain perception, coping mechanisms, and general pharmacological sensitivity vary between male and female patients (Fillingim, 2002; Frot et al., 2004; Garofalo et al., 2006; Gallagher, 2010; Gazerani et al., 2021; Failla et al., 2023). Additionally, risk factors for certain CPS conditions—such as pregnancy, motherhood, and hormonal fluctuations across a woman’s lifespan—are specific to females (Wijnhoven et al., 2006; Fattore and Fratta, 2010).

Here, each patient ultimately reached a personalized dosage regimen tailored to their overall needs (Table 1). To facilitate comparisons between individual symptomatic outcomes and CPS subtypes (see Supplementary Material S1), we developed the General Outcome Score (GOS, see Table 2) as a composite measure reflecting both benefits and potential trade-offs. It may also be useful for analyzing side effects and changes in previously used medications (see Table 8). The lowest possible GOS is −2.0, and the maximum is 2.0. All patients showed a positive GOS, ranging from 0.4 to 2.0, with a mean of 1.08 ± 0.43, indicating that every participant reported greater satisfaction with their treatment following the inclusion of FCE. While the small sample size limits definitive conclusions, no clear relationship emerged between dosage levels, CBD/THC ratios, or treatment outcomes across different CPS subtypes (see Supplementary Material S1). Therefore, although our data do not support specific recommendations regarding optimal extract composition or dosage for each CPS condition, the findings suggest that treating chronic pain with an individualized dosage scheme and as a syndromic entity—rather than as isolated subtypes—can be effective. The flexible, individualized titration protocol evaluated here, therefore, successfully addressed a broad spectrum of symptoms, including pain, regardless of the CPS subtype.

#### 4.2.1 Dosage scheme, safety concerns, and efficacy

Cannabis exerts its effects primarily via CB1 and CB2 receptors, with Δ9-tetrahydrocannabinol (THC) producing both therapeutic and adverse psychotropic outcomes, including euphoria, altered perception, and—above certain thresholds—acute impairment of memory, attention, and coordination (Volkow et al., 2014; National Academies of Sciences and Medicine, 2017; Bigand et al., 2019; Arnold, 2021). Cannabidiol (CBD), while also psychoactive in a broad sense, lacks euphoric properties and can mitigate some THC-induced anxiety or psychotomimetic effects, while exerting anxiolytic, antipsychotic, and anti-inflammatory actions via multiple receptor systems (Niesink and van Laar, 2013; Blessing et al., 2015; Ibeas Bih et al., 2015; Stauch et al., 2021; Englund et al., 2023). Adverse effects from medical cannabinoids are usually mild—dry mouth, dizziness, fatigue, appetite changes—and serious events are rare under medical supervision (Whiting et al., 2015; Stolar et al., 2022; Guldager et al., 2024; Lusawat et al., 2025). Nevertheless, long-term heavy cannabis abuse has been linked to cannabis use disorder, cognitive decline, and psychosis in vulnerable individuals (Meier et al., 2012; Niesink and van Laar, 2013; Di Forti et al., 2014), underscoring the need for cautious dosing, especially in developing, pregnant, or lactating patients, as well as in those genetically sensitive to the psychotomimetic effects of THC.

Dose thresholds are critical for safety assessment. In controlled settings with pure oral THC, acute cognitive effects emerge at ∼15 mg, with pronounced impairment above 25 mg, whereas at 10 mg subjective effects were perceptible but not performance-impairing (Curran et al., 2002; Schlienz et al., 2020). High-dose trials (≥30 mg pure THC) report transient moderate effects such as anxiety or sedation (Rozanc et al., 2024). In combined CBD–THC formulations (e.g., Sativex), 16–27 mg THC/day is considerate safe, while >30 mg/day often increases adverse events, and clinical recommendations rarely exceed 40 mg/day (Christensen et al., 2022). CBD was shown to attenuates specific THC-induced effects relevant to psychosis in a dose/ratio-dependent manner (Ganesh et al., 2022), although at high CBD doses pharmacokinetic interactions may enhance THC exposure and side effects (Zamarripa et al., 2023).

Pure CBD itself has a wide therapeutic window, with trials showing good tolerability up to 20–90 mg/kg/day (Taylor et al., 2018; Perkins et al., 2020; Hosseini et al., 2021). In the context of epilepsy, CBD is considered safe even at doses of up to 1,400 mg/day in humans (Devinsky et al., 2017; Devinsky et al., 2018). Common pure CBD side effects include, mild to moderate somnolence, gastrointestinal discomfort, and changes in appetite or weight, with lower incidence at typical clinical doses for non-epileptic conditions (100–300 mg/day) (Iffland and Grotenhermen, 2017; Millar et al., 2019; van de Donk et al., 2019; Tang et al., 2022).

The data presented above indicate that both the final doses of CBD and THC and their relative ratio are critical determinants of the therapeutic outcomes of cannabinoid-based treatments. In this report, FCEs were used with individualized CBD: THC dosages and proportions, titrated to patient response and tolerability (Table 1; Supplementary Material S1). The final mean THC dose was 10.81 mg/day (range: 1.80–22.50 mg/day) and the final mean CBD dose was 5.83 mg/day (range: 0.12–90.00 mg/day), divided into up to three daily intakes. The mean final CBD:THC ratio was 3.31: 1, with five different ratios used initially and six in the final regimen. Ratio changes occurred in 10 patients, while THC dose changed in 16 patients and CBD dose in 17 patients; reflecting the individualized titration approach. These regimens, even at the highest doses, were well within safety ranges established for combined formulations and likely benefited from individually adjusted dosing, which may have contributed to the mild side-effect profile observed. No consistent general correlation between cannabinoids dose, or cannabinoids ratio, and clinical outcome (GOS) was found (Supplementary Figure S6), highlighting the likely predominance of individual patient-specific factors over generalized dosage protocols in determining the optimal regimen in our sample.

### 4.3 Limitations

While randomized clinical trials (RCTs) remain the gold standard in clinical research, they pose challenges for cannabinoid therapies due to the vast variability in cannabis formulations and the genetically influenced differences in individual responses. These complexities make standardization of dosages and compositions difficult and highlight the value of real-world evidence (RWE) during this still exploratory stage of cannabinoid research. RWE studies offer broader inclusion criteria, flexible dosing, and the ability to address syndromic conditions and comorbidities beyond narrowly defined core symptoms (Baumfeld Andre et al., 2020; Banerjee et al., 2022). They also enhance ecological validity and may detect long-term or rare side effects, which are often missed in controlled trials.

In our study, the real-life approach—employing personalized FCE dosages across diverse CPS conditions, with long-term use and psychosocial evaluation—was essential to capture therapeutic outcomes realistically. However, inherent limitations must be acknowledged (Crisafulli et al., 2023). Patients who experienced positive outcomes may have been more likely to complete the retrospective, cross-sectional online survey, introducing possible selection bias. Additionally, the absence of direct researcher-participant interaction during the survey process, while minimizing interviewer influence, meant that the survey data were based solely on self-reported subjective patient perception (except for side effects and other medications, which were complemented with information from clinical records).

Ultimately, pain, lack of libido, fatigue, and mood disorders, for instance, are internal, inherently subjective experiences, making them largely inaccessible to direct quantitative measurement—especially in their chronic forms. Therefore, self-reported outcome instruments are essential tools for assessing the efficacy of treatments for CPS and related mood or functional disorders, as they provide unique access to the patient’s pain experience and overall wellbeing (Mosko et al., 1989; Schierhout and Myers, 1996; Bovier et al., 2004; Cuijpers et al., 2010; Morso et al., 2011; Karibe et al., 2014; Altenstein-Yamanaka et al., 2017; Ohno et al., 2017; Murphy et al., 2018; Ben Tekaya et al., 2019; Vincent et al., 2019; Riddle and Reza Jafarzadeh, 2022; Turnbull et al., 2022; Eiger et al., 2024; Gupta et al., 2024; Huang et al., 2025). In fact, self-report measures are often regarded as the “gold standard” for evaluating pain-related outcomes, particularly when supplemented with objective indicators such as changes in the use of rescue treatments (i.e., additional medications or interventions) (Dworkin et al., 2005), as was done in this report.

Despite their strengths, self-reported outcome instruments pose notable methodological challenges. On the positive side, they are easy to administer, cost-effective, and capable of capturing nuanced, individual variations in symptoms that may escape clinical observation—provided they are carefully designed and clearly communicated to ensure patient comprehension (Turk et al., 2003; Turk et al., 2008; Patrick et al., 2011b; a). In the context of chronic pain research, such tools offer direct insights into pain intensity, affective distress, and the psychosocial and functional impacts on daily life—dimensions that are not adequately captured by physiological markers alone (Dworkin et al., 2005; Turk et al., 2016). Likewise, mood disorders such as depression and anxiety often manifest internally, making self-assessment scales crucial for diagnosis and monitoring (Kroenke et al., 2001).

In this study, we adapted elements from multiple instruments to develop a customized, multifactorial, yet simplified, Patient-Reported Outcome Survey. This tool was designed to allow patients to easily and simultaneously evaluate multiple aspects of their CPS using a unified, non-numerical scale (later translated into numeric scores for analysis). However, like all self-report tools—particularly those used in open-label or non-blinded designs, which is the case here—our instrument is susceptible to several sources of response bias. These include social desirability, recall inaccuracies, participant expectations, placebo effects, and behavioral changes stemming from participants’ awareness of being observed, i.e., the Hawthorne effect (Enck et al., 2013; Hrobjartsson et al., 2014; Shiozawa et al., 2014; Chiarotto et al., 2019; Wang et al., 2023). In our study, we relied on three key aspects to help mitigate these potential sources of bias: 1) the duration of treatment, which exceeded 6 months for most participants, likely reducing the influence of short-term placebo responses and patient expectations, as well as the impact of possible Hawthorne effect; 2) the observation of reduction in the use of other medications—both for pain and mood disorders—which offer supportive and objective evidence of treatment impact, and 3) the use of an online survey format, completed without any direct contact with physicians or researchers, which may have helped reduce social desirability bias as well as the Hawthorne effect.

The original treatment protocol was not designed as a research intervention, but rather as compassionate clinical care conducted by the physician. Lacking a standardized baseline, we developed a custom cross-sectional outcome survey to assess psychological, functional, and symptomatic aspects of CPS using a single response scale. Although this limits direct numerical comparisons with other studies, the use of a narrow scale enhanced comparability across patients and better captured the complexity of chronic pain syndromes as experienced individually. It also helped mitigate the limitations inherent in assigning numerical values to subjective perceptions—an issue particularly problematic given the significant influence of emotional states and hormonal levels on pain perception (Wijnhoven et al., 2006; Reicherts et al., 2013; Lumley et al., 2021). Thus, the focus of this study was not statistical generalization, but rather an in-depth understanding of patient-reported outcomes across multiple CPS presentations.

We also developed our own questionnaire to assess adverse effects associated with cannabis extract use because conventional instruments—such as the Common Terminology Criteria for Adverse Events (CTCAE), the UKU Side Effect Rating Scale, and the Systematic Assessment for Treatment Emergent Events (SAFTEE)—do not specifically address some acute or subjective effects commonly associated with cannabis, including red eyes, and the sensation of feeling high (Levine and Schooler, 1986; Lingjaerde et al., 1987; Trotti et al., 2003). Although often mild, these effects are clinically relevant when evaluating the tolerability of cannabinoid-based therapies. By including cannabis-specific symptoms, we aimed to enhance the ecological validity of our study and provide a more comprehensive picture of real-world patient experiences.

Furthermore, our questionnaire captured not only the presence of adverse effects but also their intensity (mild, moderate, or severe) and temporality (transient vs. persistent). This level of detail is typically absent from standard instruments, which tend to focus on general or class-specific events and rarely incorporate patient-centered measures of symptom duration or subjective severity. We believe our approach yields a more nuanced and clinically meaningful profile of tolerability, supporting better-informed therapeutic decisions in real-life settings.

Finally, our Patient-Reported Outcome Survey has not yet undergone formal psychometric validation for use as a generalizable statistical instrument to support comparisons across different studies. This represents an important next step in our research agenda, but it falls beyond the scope of the present article.

## 5 Closing remarks

Our study provides compelling real-world evidence of the broad, integrative benefits of full-spectrum cannabis extracts (FCEs) for women with chronic pain syndromes (CPS). While further validation through RCTs and larger samples are necessary to eventually establish general guidelines for each CPS condition and to enable a clearer distinction between placebo and pharmacologically mediated therapeutic components, the individualized reductions in pharmaceutical burden and perceived improvements in pain, psychological distress, cognitive and motor function, and overall well-being observed here are fully consistent with the extensive body of previous pharmacological data.

The flexible, individualized dosing protocol used here—allowing adjustment of both dose and CBD:THC ratio—proved effective across a diverse group of patients and CPS conditions, encompassing nine main ICD categories formed independently of clinician or researcher bias. Positive outcomes were reported in all conditions, including fibromyalgia (n = 8), migraine (n = 6), back pain (n = 4), and coxarthrosis (n = 1). These conditions are not only representative of CPS but are also more common among women or influenced by sex-specific factors such as pregnancy and motherhood (Chitnavis et al., 2000; Toomey, 2008; Ulucay et al., 2013; Heidari et al., 2017; Marques et al., 2017; Arout et al., 2018; Lee H. J. et al., 2018; Shijagurumayum Acharya et al., 2019; Tavares et al., 2020; Rossi et al., 2022; Shetty et al., 2022; Park et al., 2023; Shi et al., 2023). In fact, as reported by patients here (see Supplementary Material S3), chronic musculoskeletal pain, in general, has been linked to hormonal and reproductive processes (Wijnhoven et al., 2006), and all CPS conditions in female patients are often compounded by caregiving and domestic responsibilities, which disproportionately affect women (Brazilian-Institute-of-Geography-and-Statistics, 2021; Melander, 2023). Given these physiological, psychological, and socio-cultural factors (Fillingim, 2000; Belfer, 2017; Planelles et al., 2020; Ramos et al., 2020; Blanton et al., 2021; Gazerani et al., 2021; Morales-Fernandez et al., 2021; Zhang H. et al., 2021; Aviram et al., 2022; Gok et al., 2022; Nguena Nguefack et al., 2022; Presto et al., 2022; Failla et al., 2023; Ghazisaeidi et al., 2023; Shen et al., 2023), our findings contribute to a more nuanced understanding of CPS in women and suggest cannabinoids as a promising adjunctive or main treatment option. Our patient-reported outcome survey, combining structured metrics with qualitative content analysis of open-ended responses, offered deep insight into how FCEs reduced physical and psychological suffering and improved functionality in daily life—even among those already on conventional medications.

These findings support the inclusion of FCEs in integrative care approaches and justify further clinical investigation. Although this study used locally produced FCEs, the flexible dosage protocol applied here may be adaptable to similar formulations elsewhere. Exploratory real-world studies like this are essential for informing future RCT design and expanding evidence-based options for chronic pain management in women.

## Data Availability

The original contributions presented in the study are included in the article/Supplementary Material, further inquiries can be directed to the corresponding author.

## Supplementary material

Supplementary Material S1 Individualized dosage protocols detailed per patient, organized by chronic pain syndrome category. CPS: chronic pain syndrome category; ICD: code in the International Classification of Diseases; ICDs: codes in the International Classification of Diseases; Wgt: weight; CBD: cannabidiol; THC: tetrahydrocannabinol.

Supplementary Material S2 Online Survey form.

Supplementary Material S3 Complete text of open-ended answers from each patient.

Supplementary Material S4 Certificates of cannabinoid analyses - AMAME Society.

Supplementary Material S5 Certificates of cannabinoid analyses - Maria Flor Society.

Supplementary Material S6 Final cannabinoids dosing and ratios vs clinical response. **(A)** Final THC dose (mg/day) vs. General Outcome Score (GOS). **(B)** Final CBD dose (mg/day) vs. GOS. **(C)** Final THC dose (mg/kg/day) vs. GOS. **(D)** Final CBD dose (mg/kg/day) vs. GOS. **(E)** Final CBD:THC ratio (mg/day) vs. GOS. Each filled circle represents one patient in panels A–D (n = 29). Final doses and ratios correspond to the stabilized regimens reported in Table 1 and Supplementary Material 1. The General Outcome Score (GOS) represents the mean of nine symptomatic categories, each rated from −2 (considerable worsening) to +2 (considerable improvement); higher values indicate greater perceived benefit. Because all patients reported positive GOS values, negative values are omitted from the x-axis for clarity. The strength of the associations between cannabinoid variables and GOS is expressed by the coefficient of determination (r). Values close to 1 denote a strong correlation, 0.3–0.5 indicate a moderate correlation, values below 0.3 reflect weak correlation, and values near 0 suggest no meaningful correlation. In this dataset, no consistent or robust correlations were observed across final doses or ratios, underscoring that individual patient-specific factors predominate over generalized dosage rules in determining clinical outcomes.

## References

[B1] Abaah D. Ohene L. A. Adjei C. A. (2023). Physical and social wellbeing of family caregivers of persons with hepatitis B associated chronic liver disease in Ghana: a qualitative study. BMC Prim. Care 24 (1), 82. 10.1186/s12875-023-02041-5 36964491 PMC10039581

[B2] Aebischer J. H. Dieckmann N. F. Jones K. D. St John A. W. (2022). Chronic pain clinical and prescriptive practices in the cannabis era. Pain Manag. Nurs. 23 (2), 109–121. 10.1016/j.pmn.2021.11.009 34973920

[B3] Aggarwal S. K. Carter G. T. Sullivan M. D. ZumBrunnen C. Morrill R. Mayer J. D. (2009). Medicinal use of cannabis in the United States: historical perspectives, current trends, and future directions. J. Opioid Manag. 5 (3), 153–168. 10.5055/jom.2009.0016 19662925

[B4] Altenstein-Yamanaka D. Zimmermann J. Krieger T. Dorig N. Grosse Holtforth M. (2017). Self-reported interpersonal problems and impact messages as perceived by significant others are differentially associated with the process and outcome of depression therapy. J. Couns. Psychol. 64 (4), 410–423. 10.1037/cou0000221 28481559

[B5] Anderson L. L. Doohan P. T. Oldfield L. Kevin R. C. Arnold J. C. Berger M. (2021). Citalopram and cannabidiol: *in vitro* and *in vivo* evidence of pharmacokinetic interactions relevant to the treatment of anxiety disorders in young people. J. Clin. Psychopharmacol. 41 (5), 525–533. 10.1097/JCP.0000000000001427 34121064

[B6] Andreae M. H. Carter G. M. Shaparin N. Suslov K. Ellis R. J. Ware M. A. (2015). Inhaled cannabis for chronic neuropathic pain: a meta-analysis of individual patient data. J. Pain 16 (12), 1221–1232. 10.1016/j.jpain.2015.07.009 26362106 PMC4666747

[B7] Arnold J. C. (2021). A primer on medicinal cannabis safety and potential adverse effects. Aust. J. Gen. Pract. 50 (6), 345–350. 10.31128/AJGP-02-21-5845 34059837

[B8] Arout C. A. Sofuoglu M. Bastian L. A. Rosenheck R. A. (2018). Gender differences in the prevalence of fibromyalgia and in concomitant medical and psychiatric disorders: a national veterans health administration study. J. Womens Health (Larchmt) 27 (8), 1035–1044. 10.1089/jwh.2017.6622 29608126 PMC6425926

[B9] Assarroudi A. Heshmati Nabavi F. Armat M. R. Ebadi A. Vaismoradi M. (2018). Directed qualitative content analysis: the description and elaboration of its underpinning methods and data analysis process. J. Res. Nurs. 23 (1), 42–55. 10.1177/1744987117741667 34394406 PMC7932246

[B10] Avello L. M. Pastene N. E. Fernandez R. P. Cordova M. P. (2017). Therapeutic potential of Cannabis sativa. Rev. Med. Chil. 145 (3), 360–367. 10.4067/S0034-98872017000300010 28548193

[B11] Aviram J. Lewitus G. M. Vysotski Y. Berman P. Shapira A. Procaccia S. (2022). Sex differences in medical cannabis-related adverse effects. Pain 163 (5), 975–983. 10.1097/j.pain.0000000000002463 34538843 PMC9009319

[B12] Bajic D. Monory K. Conrad A. Maul C. Schmid R. M. Wotjak C. T. (2018). Cannabinoid receptor type 1 in the brain regulates the affective component of visceral pain in mice. Neuroscience 384, 397–405. 10.1016/j.neuroscience.2018.05.041 29885522

[B13] Banerjee R. E. Salazar S. Mangal O. Couch N. Pacchetti D. Sodergren B. (2022). Real world evidence in medical cannabis research. Ther. Innov. Regul. Sci. 56 (1), 8–14. 10.1007/s43441-021-00346-0 34748204 PMC8688379

[B14] Bar-Lev Schleider L. Mechoulam R. Lederman V. Hilou M. Lencovsky O. Betzalel O. (2018). Prospective analysis of safety and efficacy of medical cannabis in large unselected population of patients with cancer. Eur. J. Intern Med. 49, 37–43. 10.1016/j.ejim.2018.01.023 29482741

[B15] Bardhi K. Coates S. Watson C. J. W. Lazarus P. (2022). Cannabinoids and drug metabolizing enzymes: potential for drug-drug interactions and implications for drug safety and efficacy. Expert Rev. Clin. Pharmacol. 15 (12), 1443–1460. 10.1080/17512433.2022.2148655 36384377

[B16] Bardin L. (2004). “Análise de conteúdo. 3^a^ ,”Edições, 70 1 223. Lisboa.

[B17] Baron E. P. (2018). Medicinal properties of cannabinoids, terpenes, and flavonoids in cannabis, and benefits in migraine, headache, and pain: an update on current evidence and cannabis science. Headache 58 (7), 1139–1186. 10.1111/head.13345 30152161

[B18] Baumfeld Andre E. Reynolds R. Caubel P. Azoulay L. Dreyer N. A. (2020). Trial designs using real-world data: the changing landscape of the regulatory approval process. Pharmacoepidemiol Drug Saf. 29 (10), 1201–1212. 10.1002/pds.4932 31823482 PMC7687110

[B19] Belfer I. (2017). Pain in women. Agri 29 (2), 51–54. 10.5505/agri.2017.87369 28895988

[B20] Bellnier T. Brown G. W. Ortega T. R. (2018). Preliminary evaluation of the efficacy, safety, and costs associated with the treatment of chronic pain with medical cannabis. Ment. Health Clin. 8 (3), 110–115. 10.9740/mhc.2018.05.110 29955555 PMC6007634

[B21] Ben Tekaya A. Mahmoud I. Hamdi I. Hechmi S. Saidane O. Tekaya R. (2019). Depression and anxiety in spondyloarthritis: prevalence and relationship with clinical parameters and self-reported outcome measures. Turk Psikiyatri Derg. 30 (2), 90–98. Available online at: https://pubmed.ncbi.nlm.nih.gov/31487374/ . 31487374

[B22] Berger N. D. Gadotti V. M. Petrov R. R. Chapman K. Diaz P. Zamponi G. W. (2014). NMP-7 inhibits chronic inflammatory and neuropathic pain *via* block of Cav3.2 T-type calcium channels and activation of CB2 receptors. Mol. Pain 10, 77. 10.1186/1744-8069-10-77 25481027 PMC4271433

[B23] Berger G. Arora N. Burkovskiy I. Xia Y. Chinnadurai A. Westhofen R. (2019). Experimental cannabinoid 2 receptor activation by phyto-derived and synthetic cannabinoid ligands in LPS-induced interstitial cystitis in mice. Molecules 24 (23), 4239. 10.3390/molecules24234239 31766439 PMC6930590

[B24] Berger M. Amminger G. P. McGregor I. S. (2022). Medicinal cannabis for the treatment of anxiety disorders. Aust. J. Gen. Pract. 51 (8), 586–592. 10.31128/AJGP-04-21-5936 35908759

[B25] Bigand T. Anderson C. L. Roberts M. L. Shaw M. R. Wilson M. (2019). Benefits and adverse effects of cannabis use among adults with persistent pain. Nurs. Outlook 67 (3), 223–231. 10.1016/j.outlook.2018.12.014 30616866

[B26] Biz C. de Iudicibus G. Belluzzi E. Dalmau-Pastor M. Bragazzi N. L. Funes M. (2021). Prevalence of chronic pain syndrome in patients who have undergone hallux valgus percutaneous surgery: a comparison of sciatic-femoral and ankle regional ultrasound-guided nerve blocks. BMC Musculoskelet. Disord. 22 (1), 1043. 10.1186/s12891-021-04911-4 34911525 PMC8675526

[B27] Blanton H. L. Barnes R. C. McHann M. C. Bilbrey J. A. Wilkerson J. L. Guindon J. (2021). Sex differences and the endocannabinoid system in pain. Pharmacol. Biochem. Behav. 202, 173107. 10.1016/j.pbb.2021.173107 33444598 PMC8216879

[B28] Blessing E. M. Steenkamp M. M. Manzanares J. Marmar C. R. (2015). Cannabidiol as a potential treatment for anxiety disorders. Neurotherapeutics 12 (4), 825–836. 10.1007/s13311-015-0387-1 26341731 PMC4604171

[B29] Bogdan D. M. Studholme K. DiBua A. Gordon C. Kanjiya M. P. Yu M. (2022). FABP5 deletion in nociceptors augments endocannabinoid signaling and suppresses TRPV1 sensitization and inflammatory pain. Sci. Rep. 12 (1), 9241. 10.1038/s41598-022-13284-0 35655086 PMC9163147

[B30] Bornheim L. M. Everhart E. T. Li J. Correia M. A. (1993). Characterization of cannabidiol-mediated cytochrome P450 inactivation. Biochem. Pharmacol. 45 (6), 1323–1331. 10.1016/0006-2952(93)90286-6 8466552

[B31] Bovier P. A. Charvet A. Cleopas A. Vogt N. Perneger T. V. (2004). Self-reported management of pain in hospitalized patients: link between process and outcome. Am. J. Med. 117 (8), 569–574. 10.1016/j.amjmed.2004.05.020 15465505

[B32] Brazilian-Institute-of-Geography-and-Statistics (2021). “Estatísticas de Gênero Indicadores sociais das mulheres no Brasil,” in Estatísticas de Gênero Indicadores sociais das mulheres no Brasil. I.B.d.G.e.E.B.I.o.G.a.S.-. IBGE. second ed. (Estudos e Pesquisas: Informação Demográfica e Socioeconômica IBGE).

[B33] Britch S. C. Goodman A. G. Wiley J. L. Pondelick A. M. Craft R. M. (2020). Antinociceptive and immune effects of Delta-9-Tetrahydrocannabinol or cannabidiol in Male *versus* female rats with persistent inflammatory pain. J. Pharmacol. Exp. Ther. 373 (3), 416–428. 10.1124/jpet.119.263319 32179573 PMC7250366

[B34] Calmon Almeida V. da Silva Junior W. M. de Camargo O. K. de Santana Filho V. J. Oliveira G. U. Santana M. S. (2020). Do the commonly used standard questionnaires measure what is of concern to patients with low back pain? Clin. Rehabil. 34 (10), 1313–1324. 10.1177/0269215520941042 32646318

[B35] Campos C. J. Turato E. R. (2009). Content analysis in studies using the clinical-qualitative method: application and perspectives. Rev. Lat. Am. Enferm. 17 (2), 259–264. 10.1590/s0104-11692009000200019 19551282

[B36] Campos R. M. P. Aguiar A. F. L. Paes-Colli Y. Trindade P. M. P. Ferreira B. K. de Melo Reis R. A. (2021). Cannabinoid therapeutics in chronic neuropathic pain: from animal research to human treatment. Front. Physiol. 12, 785176. 10.3389/fphys.2021.785176 34916962 PMC8669747

[B37] Castro M. Kraychete D. Daltro C. Lopes J. Menezes R. Oliveira I. (2009). Comorbid anxiety and depression disorders in patients with chronic pain. Arq. Neuropsiquiatr. 67 (4), 982–985. 10.1590/s0004-282x2009000600004 20069205

[B38] Chaves C. Bittencourt P. C. T. Pelegrini A. (2020). Ingestion of a THC-rich cannabis oil in people with fibromyalgia: a randomized, double-blind, placebo-controlled clinical trial. Pain Med. 21 (10), 2212–2218. 10.1093/pm/pnaa303 33118602 PMC7593796

[B39] Chen X. Cheng H. G. Huang Y. Liu Z. Luo X. (2012). Depression symptoms and chronic pain in the community population in beijing, China. Psychiatry Res. 200 (2-3), 313–317. 10.1016/j.psychres.2012.04.013 22560805

[B40] Chiarotto A. Maxwell L. J. Ostelo R. W. Boers M. Tugwell P. Terwee C. B. (2019). Measurement properties of visual analogue scale, numeric rating scale, and pain severity subscale of the brief pain inventory in patients with low back pain: a systematic review. J. Pain 20 (3), 245–263. 10.1016/j.jpain.2018.07.009 30099210

[B41] Chitnavis J. Sinsheimer J. S. Suchard M. A. Clipsham K. Carr A. J. (2000). End-stage coxarthrosis and gonarthrosis. Aetiology, clinical patterns and radiological features of idiopathic osteoarthritis. Rheumatol. Oxf. 39 (6), 612–619. 10.1093/rheumatology/39.6.612 10888705

[B42] Christensen C. Allesø M. Rose M. Cornett C. (2022). Clinical research evidence supporting administration and dosing recommendations of medicinal cannabis as analgesic in cancer patients. J. Clin. Med. 12 (1), 307. 10.3390/jcm12010307 36615107 PMC9821014

[B43] Cilli E. Ranieri J. Guerra F. Ferri C. Di Giacomo D. (2022). Naturalizing digital and quality of life in chronic diseases: systematic review to research perspective into technological advancing and personalized medicine. Digit. Health 8, 20552076221144857. 10.1177/20552076221144857 36578515 PMC9791272

[B45] Cohen L. L. Vowles K. E. Eccleston C. (2010). The impact of adolescent chronic pain on functioning: disentangling the complex role of anxiety. J. Pain 11 (11), 1039–1046. 10.1016/j.jpain.2009.09.009 20015706

[B46] Cortese D. A. (2007). A vision of individualized medicine in the context of global health. Clin. Pharmacol. Ther. 82 (5), 491–493. 10.1038/sj.clpt.6100390 17952101

[B47] Crisafulli S. Khan Z. Karatas Y. Tuccori M. Trifiro G. (2023). An overview of methodological flaws of real-world studies investigating drug safety in the post-marketing setting. Expert Opin. Drug Saf. 22, 373–380. 10.1080/14740338.2023.2219892 37243676

[B48] Cuijpers P. Li J. Hofmann S. G. Andersson G. (2010). Self-reported *versus* clinician-rated symptoms of depression as outcome measures in psychotherapy research on depression: a meta-analysis. Clin. Psychol. Rev. 30 (6), 768–778. 10.1016/j.cpr.2010.06.001 20619943

[B49] Curran H. V. Brignell C. Fletcher S. Middleton P. Henry J. (2002). Cognitive and subjective dose-response effects of acute oral Delta 9-tetrahydrocannabinol (THC) in infrequent cannabis users. Psychopharmacol. Berl. 164 (1), 61–70. 10.1007/s00213-002-1169-0 12373420

[B50] Dehghani A. Khoramkish M. Shahsavari Isfahani S. (2019). Challenges in the daily living activities of patients with multiple sclerosis: a qualitative content analysis. Int. J. Community Based Nurs. Midwifery 7 (3), 201–210. 10.30476/IJCBNM.2019.44995 31341919 PMC6614347

[B51] Delle J. M. Gazley C. (2021). Advocating for multimodal pain management and reducing the need for opioids in the acute and chronic pain setting. Nurs. Clin. North Am. 56 (3), 357–367. 10.1016/j.cnur.2021.04.003 34366156

[B52] Devinsky O. Cross J. H. Laux L. Marsh E. Miller I. Nabbout R. (2017). Trial of cannabidiol for drug-resistant seizures in the Dravet syndrome. N. Engl. J. Med. 376(21)**,** 2011–2020. 10.1056/NEJMoa1611618 28538134

[B53] Devinsky O. Patel A. D. Thiele E. A. Wong M. H. Appleton R. Harden C. L. (2018). Randomized, dose-ranging safety trial of cannabidiol in Dravet syndrome. Neurology 90 (14), e1204–e1211. 10.1212/WNL.0000000000005254 29540584 PMC5890607

[B54] Di Forti M. Sallis H. Allegri F. Trotta A. Ferraro L. Stilo S. A. (2014). Daily use, especially of high-potency cannabis, drives the earlier onset of psychosis in cannabis users. Schizophr. Bull. 40 (6), 1509–1517. 10.1093/schbul/sbt181 24345517 PMC4193693

[B55] Di Tella M. Castelli L. Colonna F. Fusaro E. Torta R. Ardito R. B. (2015). Theory of mind and emotional functioning in fibromyalgia syndrome: an investigation of the relationship between social cognition and executive function. PLoS One 10 (1), e0116542. 10.1371/journal.pone.0116542 25594169 PMC4296933

[B56] Duenas M. Ojeda B. Salazar A. Mico J. A. Failde I. (2016). A review of chronic pain impact on patients, their social environment and the health care system. J. Pain Res. 9, 457–467. 10.2147/JPR.S105892 27418853 PMC4935027

[B57] Dworkin R. H. Turk D. C. Farrar J. T. Haythornthwaite J. A. Jensen M. P. Katz N. P. (2005). Core outcome measures for chronic pain clinical trials: IMMPACT recommendations. Pain 113 (1-2), 9–19. 10.1016/j.pain.2004.09.012 15621359

[B58] Eiger B. Straszek C. L. Pate J. W. Rathleff M. S. (2024). Translation, contextual adaptation, and reliability of the Danish concept of pain inventory (COPI-Adult (DK)) - a self-reported outcome measure. Scand. J. Pain 24 (1), 20230092. 10.1515/sjpain-2023-0092 38452182

[B59] Enck P. Bingel U. Schedlowski M. Rief W. (2013). The placebo response in medicine: minimize, maximize or personalize? Nat. Rev. Drug Discov. 12 (3), 191–204. 10.1038/nrd3923 23449306

[B60] Engeleit A. Crosby S. Schuh M. J. (2021). Implications of cannabidiol in pharmacogenomic-based drug interactions with CYP2C19 substrates. Sr. Care Pharm. 36 (12), 674–680. 10.4140/TCP.n.2021.674 34861907

[B61] Englund A. Oliver D. Chesney E. Chester L. Wilson J. Sovi S. (2023). Does cannabidiol make cannabis safer? A randomised, double-blind, cross-over trial of cannabis with four different CBD:THC ratios. Neuropsychopharmacology 48 (6), 869–876. 10.1038/s41386-022-01478-z 36380220 PMC10156730

[B62] Erku D. Shrestha S. Scuffham P. (2021). Cost-effectiveness of medicinal cannabis for management of refractory symptoms associated with chronic conditions: a systematic review of economic evaluations. Value Health 24 (10), 1520–1530. 10.1016/j.jval.2021.04.1276 34593176

[B63] Failla M. D. Beach P. A. Atalla S. Dietrich M. S. Bruehl S. P. Cowan R. L. (2023). Gender differences in pain threshold, unpleasantness, and descending pain modulatory activation across the adult life span: a cross sectional study. J. Pain 25, 1059–1069. 10.1016/j.jpain.2023.10.027 37956742 PMC10960699

[B64] Fallon M. Dierberger K. Leng M. Hall P. S. Allende S. Sabar R. (2022). An international, open-label, randomised trial comparing a two-step approach *versus* the standard three-step approach of the WHO analgesic ladder in patients with cancer. Ann. Oncol. 33, 1296–1303. 10.1016/j.annonc.2022.08.083 36055465

[B65] Faria-Schutzer D. B. Surita F. G. Alves V. L. P. Bastos R. A. Campos C. J. G. Turato E. R. (2021). Seven steps for qualitative treatment in health research: the clinical-qualitative content analysis. Cien Saude Colet. 26 (1), 265–274. 10.1590/1413-81232020261.07622019 33533847

[B66] Fattore L. Fratta W. (2010). How important are sex differences in cannabinoid action? Br. J. Pharmacol. 160 (3), 544–548. 10.1111/j.1476-5381.2010.00776.x 20590564 PMC2931555

[B67] Feinstein A. Meza C. Stefan C. Staines W. R. (2021). Discontinuing cannabis improves depression in people with multiple sclerosis: a short report. Mult. Scler. 27 (4), 636–639. 10.1177/1352458520934070 32589554

[B68] Fillingim R. B. (2000). Sex, gender, and pain: women and men really are different. Curr. Rev. Pain 4 (1), 24–30. 10.1007/s11916-000-0006-6 10998712

[B69] Fillingim R. B. (2002). Sex differences in analgesic responses: evidence from experimental pain models. Eur. J. Anaesthesiol. Suppl. 26, 16–24. 10.1097/00003643-200219261-00004 12512212

[B70] Fitzcharles M. A. Ste-Marie P. A. Hauser W. Clauw D. J. Jamal S. Karsh J. (2016). Efficacy, tolerability, and safety of cannabinoid treatments in the rheumatic diseases: a systematic review of randomized controlled trials. Arthritis Care Res. Hob. 68 (5), 681–688. 10.1002/acr.22727 26548380

[B71] Fleury-Teixeira P. Caixeta F. V. Ramires da Silva L. C. Brasil-Neto J. P. Malcher-Lopes R. (2019). Effects of CBD-enriched Cannabis sativa extract on autism spectrum disorder symptoms: an observational study of 18 participants undergoing compassionate use. Front. Neurol. 10, 1145. 10.3389/fneur.2019.01145 31736860 PMC6834767

[B72] Fontanella B. Campos C. Turato E. (2006). Data collection in clinical-qualitative research: use of non-directed interviews with open-ended questions by health professionals. Rev. latino-americana Enferm. 14, 812–820. 10.1590/S0104-11692006000500025 17117269

[B73] Frot M. Feine J. S. Bushnell C. M. (2004). Sex differences in pain perception and anxiety. A psychophysical study with topical capsaicin. Pain 108 (3), 230–236. 10.1016/j.pain.2003.11.017 15030942

[B74] Gallagher R. M. (2010). Gender differences in the affective processing of pain: brain neuroscience and training in “biopsychosocial” pain medicine. Pain Med. 11 (9), 1311–1312. 10.1111/j.1526-4637.2010.00944.x 21050379

[B75] Ganesh S. Cortes-Briones J. Schnakenberg Martin A. M. Skosnik P. D. D'Souza D. C. Ranganathan M. (2022). Delta-9-Tetrahydrocannabinol, cannabidiol, and acute psychotomimetic states: a balancing act of the principal phyto-cannabinoids on human brain and behavior. Cannabis Cannabinoid Res. 8, 846–856. 10.1089/can.2021.0166 35319274 PMC10589482

[B76] Garofalo J. P. Lawler C. Robinson R. Morgan M. Kenworthy-Heinige T. (2006). The role of mood states underlying sex differences in the perception and tolerance of pain. Pain Pract. 6 (3), 186–196. 10.1111/j.1533-2500.2006.00084.x 17147596

[B77] Gazerani P. Aloisi A. M. Ueda H. (2021). Editorial: differences in pain biology, perception, and coping strategies: towards sex and gender specific treatments. Front. Neurosci. 15, 697285. 10.3389/fnins.2021.697285 34220445 PMC8253513

[B78] Ghazisaeidi S. Muley M. M. Salter M. W. (2023). Neuropathic pain: mechanisms, sex differences, and potential therapies for a global problem. Annu. Rev. Pharmacol. Toxicol. 63, 565–583. 10.1146/annurev-pharmtox-051421-112259 36662582

[B79] Gok K. Erol K. Kilic G. Kilic E. Ozgocmen S. (2022). Gender differences in the discriminative value of inflammatory low back pain criteria. ARP Rheumatol. 1 (4), 293–299. Available online at: https://pubmed.ncbi.nlm.nih.gov/36617312/ . 36617312

[B80] Goldberg D. S. McGee S. J. (2011). Pain as a global public health priority. BMC Public Health 11 (1), 770–775. 10.1186/1471-2458-11-770 21978149 PMC3201926

[B81] Gonzalez-Sepulveda M. Pozo O. J. Marcos J. Valverde O. (2016). Chronic pain causes a persistent anxiety state leading to increased ethanol intake in CD1 mice. J. Psychopharmacol. 30 (2), 188–203. 10.1177/0269881115622238 26681793

[B82] Grabli F. E. Quesque F. Borg C. Witthoft M. Michael G. A. Lucas C. (2021). Interoception and social cognition in chronic low back pain: a common inference disturbance? An exploratory study. Pain Manag. 12, 471–485. 10.2217/pmt-2021-0090 34894713

[B83] Graneheim U. H. Lindgren B. M. Lundman B. (2017). Methodological challenges in qualitative content analysis: a discussion paper. Nurse Educ. Today 56, 29–34. 10.1016/j.nedt.2017.06.002 28651100

[B84] Greenfield J. A. Kimberly L. L. Berman Z. P. Ramly E. P. Alfonso A. R. Lee O. (2020). Perceptions of quality of life among face transplant recipients: a qualitative content analysis. Plast. Reconstr. Surg. Glob. Open 8 (8), e2956. 10.1097/GOX.0000000000002956 32983761 PMC7489701

[B85] Groenewald C. B. Essner B. S. Wright D. Fesinmeyer M. D. Palermo T. M. (2014). The economic costs of chronic pain among a cohort of treatment-seeking adolescents in the United States. J. Pain 15 (9), 925–933. 10.1016/j.jpain.2014.06.002 24953887 PMC4150826

[B86] Grove B. E. Valen Schougaard L. M. Ivarsen P. Hjollund N. H. de Thurah A. Mejdahl C. T. (2023). Remote follow-up based on patient-reported outcomes in patients with chronic kidney disease: a qualitative study of patient perspectives. PLoS One 18 (2), e0281393. 10.1371/journal.pone.0281393 36763600 PMC9916608

[B87] Gruber S. A. Smith R. T. Dahlgren M. K. Lambros A. M. Sagar K. A. (2021). No pain, all gain? Interim analyses from a longitudinal, observational study examining the impact of medical cannabis treatment on chronic pain and related symptoms. Exp. Clin. Psychopharmacol. 29 (2), 147–156. 10.1037/pha0000435 33764103

[B88] Guldager M. B. Chaves Filho A. M. Biojone C. Joca S. (2024). Therapeutic potential of cannabidiol in depression. Int. Rev. Neurobiol. 177, 251–293. 10.1016/bs.irn.2024.06.001 39029987

[B89] Gupta A. Johnson S. Barraclough M. Su J. Bingham K. Knight A. M. (2024). Outcome clusters and their stability over 1 year in patients with SLE: self-reported and performance-based cognitive function, disease activity, mood and health-related quality of life. Lupus Sci. Med. 11 (2), e001006. 10.1136/lupus-2023-001006 38991833 PMC11243126

[B90] Heidari F. Afshari M. Moosazadeh M. (2017). Prevalence of fibromyalgia in general population and patients, a systematic review and meta-analysis. Rheumatol. Int. 37 (9), 1527–1539. 10.1007/s00296-017-3725-2 28447207

[B91] Hosseini A. McLachlan A. J. Lickliter J. D. (2021). A phase I trial of the safety, tolerability and pharmacokinetics of cannabidiol administered as single-dose oil solution and single and multiple doses of a sublingual wafer in healthy volunteers. Br. J. Clin. Pharmacol. 87 (4), 2070–2077. 10.1111/bcp.14617 33075170

[B92] Hrobjartsson A. Thomsen A. S. Emanuelsson F. Tendal B. Rasmussen J. V. Hilden J. (2014). Observer bias in randomized clinical trials with time-to-event outcomes: systematic review of trials with both blinded and non-blinded outcome assessors. Int. J. Epidemiol. 43 (3), 937–948. 10.1093/ije/dyt270 24448109

[B93] Hryhorowicz S. Walczak M. Zakerska-Banaszak O. Slomski R. Skrzypczak-Zielinska M. (2018). Pharmacogenetics of cannabinoids. Eur. J. Drug Metab. Pharmacokinet. 43 (1), 1–12. 10.1007/s13318-017-0416-z 28534260 PMC5794848

[B94] Hsieh H. F. Shannon S. E. (2005). Three approaches to qualitative content analysis. Qual. Health Res. 15 (9), 1277–1288. 10.1177/1049732305276687 16204405

[B95] Huang F. F. Liang J. Lin C. Y. Samartzis D. Karppinen J. Zheng Y. (2025). Measurement properties of self-reported outcome measures for older adults with nonspecific low back pain: a systematic review. Age Ageing 54 (3), afaf045. 10.1093/ageing/afaf045 40139218 PMC11942786

[B96] Hylands-White N. Duarte R. V. Raphael J. H. (2017). An overview of treatment approaches for chronic pain management. Rheumatol. Int. 37 (1), 29–42. 10.1007/s00296-016-3481-8 27107994

[B97] Ibeas Bih C. Chen T. Nunn A. V. Bazelot M. Dallas M. Whalley B. J. (2015). Molecular targets of cannabidiol in neurological disorders. Neurotherapeutics 12 (4), 699–730. 10.1007/s13311-015-0377-3 26264914 PMC4604182

[B98] Iffland K. Grotenhermen F. (2017). An update on safety and side effects of cannabidiol: a review of clinical data and relevant animal studies. Cannabis Cannabinoid Res. 2 (1), 139–154. 10.1089/can.2016.0034 28861514 PMC5569602

[B99] Jackson T. Thomas S. Stabile V. Han X. Shotwell M. McQueen K. (2015). Prevalence of chronic pain in low-income and middle-income countries: a systematic review and meta-analysis. Lancet 385, S10. 10.1016/S0140-6736(15)60805-4 26313056

[B100] Jiang R. Yamaori S. Okamoto Y. Yamamoto I. Watanabe K. (2013). Cannabidiol is a potent inhibitor of the catalytic activity of cytochrome P450 2C19. Drug Metab. Pharmacokinet. 28 (4), 332–338. 10.2133/dmpk.dmpk-12-rg-129 23318708

[B101] Jones R. T. Benowitz N. L. Herning R. I. (1981). Clinical relevance of cannabis tolerance and dependence. J. Clin. Pharmacol. 21 (S1), 143S–152S. 10.1002/j.1552-4604.1981.tb02589.x 6271820

[B102] Joshi A. Kale S. Chandel S. Pal D. K. (2015). Likert scale: explored and explained. Br. J. Appl. Sci. and Technol. 7 (4), 396–403. 10.9734/bjast/2015/14975

[B103] Jugl S. Okpeku A. Costales B. Morris E. J. Alipour-Haris G. Hincapie-Castillo J. M. (2021). A mapping literature review of medical cannabis clinical outcomes and quality of evidence in approved conditions in the USA from 2016 to 2019. Med. Cannabis Cannabinoids 4 (1), 21–42. 10.1159/000515069 34676348 PMC8525213

[B104] Kao Y. C. Chen J. Y. Chen H. H. Liao K. W. Huang S. S. (2021). The association between depression and chronic lower back pain from disc degeneration and herniation of the lumbar spine. Int. J. Psychiatry Med. 57, 165–177. 10.1177/00912174211003760 33840233

[B105] Karibe H. Goddard G. Shimazu K. Kato Y. Warita-Naoi S. Kawakami T. (2014). Comparison of self-reported pain intensity, sleeping difficulty, and treatment outcomes of patients with myofascial temporomandibular disorders by age group: a prospective outcome study. BMC Musculoskelet. Disord. 15, 423. 10.1186/1471-2474-15-423 25496226 PMC4295233

[B106] Kashikar-Zuck S. King C. Ting T. V. Arnold L. M. (2016). Juvenile fibromyalgia: different from the adult chronic pain syndrome? Curr. Rheumatol. Rep. 18 (4), 19. 10.1007/s11926-016-0569-9 26984803

[B107] Kawai K. Kawai A. T. Wollan P. Yawn B. P. (2017). Adverse impacts of chronic pain on health-related quality of life, work productivity, depression and anxiety in a community-based study. Fam. Pract. 34 (6), 656–661. 10.1093/fampra/cmx034 28444208 PMC6260800

[B108] Khurshid H. Qureshi I. A. Jahan N. Went T. R. Sultan W. Sapkota A. (2021). A systematic review of fibromyalgia and recent advancements in treatment: is medicinal cannabis a new hope? Cureus 13 (8), e17332. 10.7759/cureus.17332 34567876 PMC8451533

[B109] Kosiba J. D. Maisto S. A. Ditre J. W. (2019). Patient-reported use of medical cannabis for pain, anxiety, and depression symptoms: systematic review and meta-analysis. Soc. Sci. Med. 233, 181–192. 10.1016/j.socscimed.2019.06.005 31207470

[B110] Kroenke K. Spitzer R. L. Williams J. B. (2001). The PHQ-9: validity of a brief depression severity measure. J. Gen. Intern Med. 16 (9), 606–613. 10.1046/j.1525-1497.2001.016009606.x 11556941 PMC1495268

[B111] Kronborg C. Handberg G. Axelsen F. (2009). Health care costs, work productivity and activity impairment in non-malignant chronic pain patients. Eur. J. Health Econ. 10 (1), 5–13. 10.1007/s10198-008-0096-3 18256865

[B112] Krustev E. Reid A. McDougall J. J. (2014). Tapping into the endocannabinoid system to ameliorate acute inflammatory flares and associated pain in mouse knee joints. Arthritis Res. Ther. 16 (5), 437. 10.1186/s13075-014-0437-9 25260980 PMC4201700

[B113] Kuhathasan N. Minuzzi L. MacKillop J. Frey B. N. (2022). An investigation of cannabis use for insomnia in depression and anxiety in a naturalistic sample. BMC Psychiatry 22 (1), 303. 10.1186/s12888-022-03948-6 35484520 PMC9052466

[B114] La Porta C. Bura S. A. Llorente-Onaindia J. Pastor A. Navarrete F. Garcia-Gutierrez M. S. (2015). Role of the endocannabinoid system in the emotional manifestations of osteoarthritis pain. Pain 156 (10), 2001–2012. 10.1097/j.pain.0000000000000260 26067584 PMC4770330

[B115] Lake S. Kerr T. Buxton J. Walsh Z. Marshall B. D. Wood E. (2020). Does cannabis use modify the effect of post-traumatic stress disorder on severe depression and suicidal ideation? Evidence from a population-based cross-sectional study of Canadians. J. Psychopharmacol. 34 (2), 181–188. 10.1177/0269881119882806 31684805

[B116] Leadley R. Armstrong N. Lee Y. Allen A. Kleijnen J. (2012). Chronic diseases in the european union: the prevalence and health cost implications of chronic pain. J. Pain and Palliat. Care Pharmacother. 26 (4), 310–325. 10.3109/15360288.2012.736933 23216170

[B117] Lee M. C. Ploner M. Wiech K. Bingel U. Wanigasekera V. Brooks J. (2013). Amygdala activity contributes to the dissociative effect of cannabis on pain perception. Pain 154 (1), 124–134. 10.1016/j.pain.2012.09.017 23273106 PMC3549497

[B118] Lee G. Grovey B. Furnish T. Wallace M. (2018a). Medical cannabis for neuropathic pain. Curr. Pain Headache Rep. 22 (1), 8. 10.1007/s11916-018-0658-8 29388063

[B119] Lee H. J. Choi E. Nahm F. S. Choi S. S. Kim Y. H. Moon J. Y. (2018b). Prevalence of fibromyalgia in fourteen Korean tertiary care university hospital pain clinics. J. Pain Res. 11, 2417–2423. 10.2147/JPR.S172221 30425555 PMC6200436

[B44] Levine J. Schooler N. R. (1986). SAFTEE: A technique for the systematic assessment of side effects in clinical trials. Psychopharmacology Bulletin. 22 (2), 343–381. Available online at: https://pubmed.ncbi.nlm.nih.gov/3774930/ . 3774930

[B120] Leo R. J. (2005). Chronic pain and comorbid depression. Curr. Treat. Options Neurol. 7 (5), 403–412. 10.1007/s11940-005-0032-0 16079044

[B121] Leung J. Lim C. C. W. Chiu V. Chung J. Mekonen T. Dawson D. (2022). Prevalence and correlates of cannabis use for medicinal reasons - an Australian cross-sectional study. Addict. Behav. Rep. 15, 100436. 10.1016/j.abrep.2022.100436 35662918 PMC9160481

[B122] Li X. Diviant J. P. Stith S. S. Brockelman F. Keeling K. Hall B. (2020). The effectiveness of cannabis flower for immediate relief from symptoms of depression. Yale J. Biol. Med. 93 (2), 251–264. Available online at: https://pmc.ncbi.nlm.nih.gov/articles/PMC7309674/ . 32607086 PMC7309674

[B123] Lim C. T. Tadmor A. Fujisawa D. MacDonald J. J. Gallagher E. R. Eusebio J. (2017). Qualitative research in palliative care: applications to clinical trials work. J. Palliat. Med. 20 (8), 857–861. 10.1089/jpm.2017.0061 28388341 PMC5564028

[B124] Lingjaerde O. Ahlfors U. G. Bech P. Dencker S. J. Elgen K. (1987). The UKU side effect rating scale. A new comprehensive rating scale for psychotropic drugs and a cross-sectional study of side effects in neuroleptic-treated patients. Acta Psychiatr. Scand. Suppl. 334, 1–100. 10.1111/j.1600-0447.1987.tb10566.x 2887090

[B125] Longo R. Oudshoorn A. Befus D. (2021). Cannabis for chronic pain: a rapid systematic review of randomized control trials. Pain Manag. Nurs. 22 (2), 141–149. 10.1016/j.pmn.2020.11.006 33353819

[B126] Lumley M. A. Krohner S. Marshall L. M. Kitts T. C. Schubiner H. Yarns B. C. (2021). Emotional awareness and other emotional processes: implications for the assessment and treatment of chronic pain. Pain Manag. 11 (3), 325–332. 10.2217/pmt-2020-0081 33533272 PMC7923252

[B127] Lundin A. Ekman I. Wallstrom S. Andrell P. Lundberg M. (2023). Suffering out of sight but not out of mind - interpreting experiences of sick leave due to chronic pain in a community setting: a qualitative study. BMJ Open 13 (4), e066617. 10.1136/bmjopen-2022-066617 37041054 PMC10106073

[B128] Lusawat A. Khongkhatithum C. Suwannachote S. Katanyuwong K. Fangsa-Ad T. Anurat K. (2025). National multicenter cohort study: adjunctive cannabidiol-enriched cannabis oil for pediatric drug-resistant epilepsy treatment in Thailand. Pediatr. Neurol. 169, 59–68. 10.1016/j.pediatrneurol.2025.04.015 40460512

[B129] Manhapra A. (2022). Complex persistent opioid Dependence-an opioid-induced chronic pain syndrome. Curr. Treat. Options Oncol. 23 (7), 921–935. 10.1007/s11864-022-00985-x 35435616

[B130] Marques A. P. Santo A. Berssaneti A. A. Matsutani L. A. Yuan S. L. K. (2017). Prevalence of fibromyalgia: literature review update. Rev. Bras. Reumatol. Engl. 57 (4), 356–363. 10.1016/j.rbre.2017.01.005 57 28743363

[B131] Mattila-Rautiainen S. Venojarvi M. Rautiainen H. Keski-Valkama A. (2023). The impact on physical performance, pain and psychological wellbeing of chronic low back pain patients during 12-weeks of equine-facilitated therapy intervention. Front. Vet. Sci. 10, 1085768. 10.3389/fvets.2023.1085768 36998640 PMC10043450

[B132] McKeon G. Parker S. Warren N. Scott J. G. (2021). The patient experience of recovery following Anti-NMDA receptor encephalitis: a qualitative content analysis. J. Neuropsychiatry Clin. Neurosci. 33 (1), 57–63. 10.1176/appi.neuropsych.20030049 32873136

[B133] Mecca C. M. Chao D. Yu G. Feng Y. Segel I. Zhang Z. (2021). Dynamic change of endocannabinoid signaling in the medial prefrontal cortex controls the development of depression after neuropathic pain. J. Neurosci. 41 (35), 7492–7508. 10.1523/JNEUROSCI.3135-20.2021 34244365 PMC8412994

[B134] Meda R. T. Nuguru S. P. Rachakonda S. Sripathi S. Khan M. I. Patel N. (2022). Chronic pain-induced depression: a review of prevalence and management. Cureus 14 (8), e28416. 10.7759/cureus.28416 36171845 PMC9509520

[B135] Meier M. H. Caspi A. Ambler A. Harrington H. Houts R. Keefe R. S. E. (2012). Persistent cannabis users show neuropsychological decline from childhood to midlife. Proc. Natl. Acad. Sci. 109(40)**,** E2657–E2664. 10.1073/pnas.1206820109 22927402 PMC3479587

[B136] Melander S. (2023). Different logics of pain: the gendered dimension of chronic pain in a relational setting. Soc. Sci. Med. 335, 116229. 10.1016/j.socscimed.2023.116229 37703783

[B137] Midenfjord I. Grinsvall C. Koj P. Carnerup I. Tornblom H. Simren M. (2021). Central sensitization and severity of gastrointestinal symptoms in irritable bowel syndrome, chronic pain syndromes, and inflammatory bowel disease. Neurogastroenterol. Motil. 33 (12), e14156. 10.1111/nmo.14156 33860970

[B138] Millar S. A. Stone N. L. Bellman Z. D. Yates A. S. England T. J. O'Sullivan S. E. (2019). A systematic review of cannabidiol dosing in clinical populations. Br. J. Clin. Pharmacol. 85 (9), 1888–1900. 10.1111/bcp.14038 31222854 PMC6710502

[B139] Mitchell V. A. Harley J. Casey S. L. Vaughan A. C. Winters B. L. Vaughan C. W. (2021). Oral efficacy of Δ(9)-tetrahydrocannabinol and cannabidiol in a mouse neuropathic pain model. Neuropharmacology 189, 108529. 10.1016/j.neuropharm.2021.108529 33741405

[B140] Morales-Fernandez A. Jimenez Martin J. M. Vergara-Romero M. Morales-Asencio J. M. Mora-Bandera A. M. Gomez-Ortigosa M. I. (2021). Gender differences in perceived pain and health-related quality of life in people with chronic non-malignant pain: a cross-sectional study. Contemp. Nurse 57 (3-4), 280–289. 10.1080/10376178.2021.1999836 34709980

[B141] Moriarty O. McGuire B. E. Finn D. P. (2011). The effect of pain on cognitive function: a review of clinical and preclinical research. Prog. Neurobiol. 93 (3), 385–404. 10.1016/j.pneurobio.2011.01.002 21216272

[B142] Morso L. Kent P. M. Albert H. B. (2011). Are self-reported pain characteristics, classified using the PainDETECT questionnaire, predictive of outcome in people with low back pain and associated leg pain? Clin. J. Pain 27 (6), 535–541. 10.1097/AJP.0b013e318208c941 21562413

[B143] Mosko S. Zetin M. Glen S. Garber D. DeAntonio M. Sassin J. (1989). Self-reported depressive symptomatology, mood ratings, and treatment outcome in sleep disorders patients. J. Clin. Psychol. 45 (1), 51–60. 10.1002/1097-4679(198901)45:1<51::aid-jclp2270450107>3.0.co;2-h 2925884

[B144] Murphy M. Rio E. Debenham J. Docking S. Travers M. Gibson W. (2018). Evaluating the progress of mid-portion achilles tendinopathy during rehabilitation: a review of outcome measures for Self- reported pain and function. Int. J. Sports Phys. Ther. 13 (2), 283–292. 10.26603/ijspt20180283 30090686 PMC6063067

[B145] Nanan J. C. Crosby S. Schuh M. J. (2022). Hyponatremic cognitive dysfunction resulting from drug-drug-gene interaction between sertraline and cannabidiol in an intermediate CYP2C19 metabolizer patient. Innov. Pharm. 13 (3), 2. 10.24926/iip.v13i3.4890 36627907 PMC9815864

[B146] National Academies of Sciences and Medicine (2017). in The health effects of cannabis and cannabinoids: the current state of evidence andRecommendations for research. Editor A T. N. (Washington, DC: Press). National Academies of Sciences, Engineering, and Medicine. 28182367

[B147] Nguena Nguefack H. L. Gabrielle Page M. Guenette L. Blais L. Diallo M. Godbout-Parent M. (2022). Gender differences in medication adverse effects experienced by people living with chronic pain. Front. Pain Res. (Lausanne) 3, 830153. 10.3389/fpain.2022.830153 35620635 PMC9128021

[B148] Niesink R. J. van Laar M. W. (2013). Does cannabidiol protect against adverse psychological effects of THC? Front. Psychiatry 4, 130. 10.3389/fpsyt.2013.00130 24137134 PMC3797438

[B149] Nowinka Z. Alagha M. A. Mahmoud K. Jones G. G. (2022). Predicting depression in patients with knee osteoarthritis using machine learning: model development and validation study. JMIR Form. Res. 6 (9), e36130. 10.2196/36130 36099008 PMC9518113

[B150] Ohno S. Takahashi K. Inoue A. Takada K. Ishihara Y. Tanigawa M. (2017). Smallest detectable change and test-retest reliability of a self-reported outcome measure: results of the center for epidemiologic studies depression scale, general self-efficacy scale, and 12-item general health questionnaire. J. Eval. Clin. Pract. 23 (6), 1348–1354. 10.1111/jep.12795 28758322

[B151] Park Y. Watkins B. A. (2021). Endocannabinoids and aging-Inflammation, neuroplasticity, mood and pain. Vitam. Horm. 115, 129–172. 10.1016/bs.vh.2020.12.007 33706946

[B152] Park H. J. Choi J. Y. Lee W. M. Park S. M. (2023). Prevalence of chronic low back pain and its associated factors in the general population of South Korea: a cross-sectional study using the national health and nutrition examination surveys. J. Orthop. Surg. Res. 18 (1), 29. 10.1186/s13018-023-03509-x 36631903 PMC9832776

[B153] Patrick D. L. Burke L. B. Gwaltney C. J. Leidy N. K. Martin M. L. Molsen E. (2011a). Content validity--establishing and reporting the evidence in newly developed patient-reported outcomes (PRO) instruments for medical product evaluation: ISPOR PRO good research practices task force report: part 1--eliciting concepts for a new PRO instrument. Value Health 14 (8), 967–977. 10.1016/j.jval.2011.06.014 22152165

[B154] Patrick D. L. Burke L. B. Gwaltney C. J. Leidy N. K. Martin M. L. Molsen E. (2011b). Content validity--establishing and reporting the evidence in newly developed patient-reported outcomes (PRO) instruments for medical product evaluation: ISPOR PRO good research practices task force report: part 2--assessing respondent understanding. Value Health 14 (8), 978–988. 10.1016/j.jval.2011.06.013 22152166

[B155] Perkins D. Butler J. Ong K. Nguyen T. H. Cox S. Francis B. (2020). A phase 1, randomised, placebo-controlled, dose escalation study to investigate the safety, tolerability and pharmacokinetics of cannabidiol in fed healthy volunteers. Eur. J. Drug Metab. Pharmacokinet. 45 (5), 575–586. 10.1007/s13318-020-00624-6 32409982 PMC7511474

[B156] Pernia-Andrade A. J. Kato A. Witschi R. Nyilas R. Katona I. Freund T. F. (2009). Spinal endocannabinoids and CB1 receptors mediate C-fiber-induced heterosynaptic pain sensitization. Science 325 (5941), 760–764. 10.1126/science.1171870 19661434 PMC2835775

[B157] Pertwee R. G. (1999). Cannabis and cannabinoids: pharmacology and rationale for clinical use. Forsch Komplementarmed 6 (Suppl. 3), 12–15. 10.1159/000057150 10575283

[B158] Phelps C. E. Navratilova E. Porreca F. (2021). Cognition in the chronic pain experience: preclinical insights. Trends Cogn. Sci. 25 (5), 365–376. 10.1016/j.tics.2021.01.001 33509733 PMC8035230

[B159] Phillips C. J. (2006). Economic burden of chronic pain. Expert Rev. Pharmacoecon Outcomes Res. 6 (5), 591–601. 10.1586/14737167.6.5.591 20528505

[B160] Phillips C. J. (2009). The cost and burden of chronic pain. Rev. Pain 3 (1), 2–5. 10.1177/204946370900300102 26526940 PMC4590036

[B161] Planelles B. Margarit C. Inda M. D. Ballester P. Muriel J. Barrachina J. (2020). Gender based differences, pharmacogenetics and adverse events in chronic pain management. Pharmacogenomics J. 20 (2), 320–328. 10.1038/s41397-019-0118-9 31745220

[B162] Pope C. Mays N. (1995). Reaching the parts other methods cannot reach: an introduction to qualitative methods in health and health services research. BMJ 311 (6996), 42–45. 10.1136/bmj.311.6996.42 7613329 PMC2550091

[B163] Presto P. Mazzitelli M. Junell R. Griffin Z. Neugebauer V. (2022). Sex differences in pain along the neuraxis. Neuropharmacology 210, 109030. 10.1016/j.neuropharm.2022.109030 35331712 PMC9354808

[B164] Racz I. Nent E. Erxlebe E. Zimmer A. (2015). CB1 receptors modulate affective behaviour induced by neuropathic pain. Brain Res. Bull. 114, 42–48. 10.1016/j.brainresbull.2015.03.005 25863168

[B165] Ramos A. K. Carvajal-Suarez M. Trinidad N. Michaud T. L. Grimm B. LeVan T. (2020). A cross-sectional study of gender-related differences in reporting fatigue and pain among Latino/A migrant farmworkers. J. Agromedicine 25 (3), 319–329. 10.1080/1059924X.2020.1713272 31941431

[B166] Razavi Y. Rashvand M. Sharifi A. Haghparast A. Keyhanfar F. Haghparast A. (2021). Cannabidiol microinjection into the nucleus accumbens attenuated nociceptive behaviors in an animal model of tonic pain. Neurosci. Lett. 762, 136141. 10.1016/j.neulet.2021.136141 34324957

[B167] Reicherts P. Gerdes A. B. Pauli P. Wieser M. J. (2013). On the mutual effects of pain and emotion: facial pain expressions enhance pain perception and *vice versa* are perceived as more arousing when feeling pain. Pain 154 (6), 793–800. 10.1016/j.pain.2013.02.012 23541426

[B168] Reid C. Davies A. (2004). The world health organization three-step analgesic ladder comes of age. Palliat. Med. 18 (3), 175–176. 10.1191/0269216304pm897ed 15198129

[B169] Reitsma M. Tranmer J. E. Buchanan D. M. VanDenKerkhof E. G. (2012). The epidemiology of chronic pain in Canadian men and women between 1994 and 2007: longitudinal results of the national population health survey. Pain Res. Manag. 17 (3), 166–172. 10.1155/2012/875924 22606681 PMC3401087

[B170] Rice A. S. Smith B. H. Blyth F. M. (2016). Pain and the global burden of disease. Pain 157 (4), 791–796. 10.1097/j.pain.0000000000000454 26670465

[B171] Riddle D. L. Reza Jafarzadeh S. (2022). Effects of psychological distress on the general health to self-reported pain and function outcome relationship in knee arthroplasty: a causal mediation study. Osteoarthr. Cartil. Open 4 (4), 100315. 10.1016/j.ocarto.2022.100315 36474788 PMC9718105

[B172] Rodriguez-Munoz M. Onetti Y. Cortes-Montero E. Garzon J. Sanchez-Blazquez P. (2018). Cannabidiol enhances morphine antinociception, diminishes NMDA-Mediated seizures and reduces stroke damage *via* the sigma 1 receptor. Mol. Brain 11 (1), 51. 10.1186/s13041-018-0395-2 30223868 PMC6142691

[B173] Rogers A. H. Garey L. Raines A. M. Allan N. P. Schmidt N. B. Zvolensky M. J. (2022). Anxiety sensitivity and opioid use motives among adults with chronic low back pain. Exp. Clin. Psychopharmacol. 30 (1), 23–30. 10.1037/pha0000381 32772532

[B174] Rossi M. F. Tumminello A. Marconi M. Gualano M. R. Santoro P. E. Malorni W. (2022). Sex and gender differences in migraines: a narrative review. Neurol. Sci. 43 (9), 5729–5734. 10.1007/s10072-022-06178-6 35676560 PMC9176156

[B175] Rozanc J. Klumpers L. E. Huestis M. A. Tagen M. (2024). Tolerability of high-dose oral Δ(9)-THC: implications for human laboratory study design. Cannabis Cannabinoid Res. 9 (2), 437–448. 10.1089/can.2023.0209 38377580

[B176] Russo E. B. (2004). Clinical endocannabinoid deficiency (CECD): can this concept explain therapeutic benefits of cannabis in migraine, fibromyalgia, irritable bowel syndrome and other treatment-resistant conditions? Neuro Endocrinol. Lett. 25 (1-2), 31–39. Available online at: https://pubmed.ncbi.nlm.nih.gov/36617312/ . 15159679

[B178] Russo E. B. (2016). Clinical endocannabinoid deficiency reconsidered: current research supports the theory in migraine, fibromyalgia, irritable bowel, and other treatment-resistant syndromes. Cannabis Cannabinoid Res. 1 (1), 154–165. 10.1089/can.2016.0009 28861491 PMC5576607

[B179] Russo E. B. Guy G. W. Robson P. J. (2007). Cannabis, pain, and sleep: lessons from therapeutic clinical trials of sativex, a cannabis-based medicine. Chem. Biodivers. 4 (8), 1729–1743. 10.1002/cbdv.200790150 17712817

[B180] Sachedina F. Chan C. Damji R. S. de Sanctis O. J. (2022). Medical cannabis use in Canada and its impact on anxiety and depression: a retrospective study. Psychiatry Res. 313, 114573. 10.1016/j.psychres.2022.114573 35598566

[B181] Santiago B. V. M. Oliveira A. B. G. Silva G. Silva M. F. D. Bergamo P. E. Parise M. (2023). Prevalence of chronic pain in Brazil: a systematic review and meta-analysis. Clin. (Sao Paulo) 78, 100209. 10.1016/j.clinsp.2023.100209 37201302 PMC10206159

[B182] Santoro A. Mele E. Marino M. Viggiano A. Nori S. L. Meccariello R. (2021). The complex interplay between endocannabinoid system and the estrogen system in central nervous system and periphery. Int. J. Mol. Sci. 22 (2), 972. 10.3390/ijms22020972 33478092 PMC7835826

[B183] Schierhout G. H. Myers J. E. (1996). Is self-reported pain an appropriate outcome measure in ergonomic-epidemiologic studies of work-related musculoskeletal disorders? Am. J. Ind. Med. 30 (1), 93–98. 10.1002/(SICI)1097-0274(199607)30:1<93::AID-AJIM16>3.0.CO;2-3 8837690

[B184] Schlienz N. J. Spindle T. R. Cone E. J. Herrmann E. S. Bigelow G. E. Mitchell J. M. (2020). Pharmacodynamic dose effects of oral cannabis ingestion in healthy adults who infrequently use cannabis. Drug Alcohol Depend. 211, 107969. 10.1016/j.drugalcdep.2020.107969 32298998 PMC8221366

[B185] Schwieger-Briel A. Chakkittakandiyil A. Lara-Corrales I. Aujla N. Lane A. T. Lucky A. W. (2015). Instrument for scoring clinical outcome of research for epidermolysis bullosa: a consensus-generated clinical research tool. Pediatr. Dermatol 32 (1), 41–52. 10.1111/pde.12317 24650374

[B186] Seidel M. F. Hugle T. Morlion B. Koltzenburg M. Chapman V. MaassenVanDenBrink A. (2022). Neurogenic inflammation as a novel treatment target for chronic pain syndromes. Exp. Neurol. 356, 114108. 10.1016/j.expneurol.2022.114108 35551902

[B187] Shen Z. Li W. Chang W. Yue N. Yu J. (2023). Sex differences in chronic pain-induced mental disorders: mechanisms of cerebral circuitry. Front. Mol. Neurosci. 16, 1102808. 10.3389/fnmol.2023.1102808 36891517 PMC9986270

[B188] Shetty G. M. Jain S. Thakur H. Khanna K. (2022). Prevalence of low back pain in India: a systematic review and meta-analysis. Work 73 (2), 429–452. 10.3233/WOR-205300 35964222

[B189] Shi Z. Yan F. Lu Y. Liu W. Wang Z. Zhang H. (2023). Pregnancy-related low back/pelvic girdle pain: prevalence, severity, and risk factors in zhengzhou, China. J. Back Musculoskelet. Rehabil. 36 (4), 895–902. 10.3233/BMR-220147 37248876

[B190] Shijagurumayum Acharya R. Tveter A. T. Grotle M. Eberhard-Gran M. Stuge B. (2019). Prevalence and severity of low back- and pelvic girdle pain in pregnant Nepalese women. BMC Pregnancy Childbirth 19 (1), 247. 10.1186/s12884-019-2398-0 31307421 PMC6631866

[B191] Shiozawa P. Duailibi M. S. da Silva M. E. Cordeiro Q. (2014). Trigeminal nerve stimulation (TNS) protocol for treating major depression: an open-label proof-of-concept trial. Epilepsy Behav. 39, 6–9. 10.1016/j.yebeh.2014.07.021 25150403

[B192] Sirianni J. Ibrahim M. Patwardhan A. (2015). Chronic pain syndromes, mechanisms, and current treatments. Prog. Mol. Biol. Transl. Sci. 131, 565–611. 10.1016/bs.pmbts.2015.01.004 25744686

[B193] Smith S. C. Wagner M. S. (2014). Clinical endocannabinoid deficiency (CECD) revisited: can this concept explain the therapeutic benefits of cannabis in migraine, fibromyalgia, irritable bowel syndrome and other treatment-resistant conditions? Neuro Endocrinol. Lett. 35 (3), 198–201. Available online at: https://pubmed.ncbi.nlm.nih.gov/24977967/ . 24977967

[B194] Stauch C. M. Ammerman B. Sepulveda D. Aynardi M. C. Garner M. R. Lewis G. (2021). Biomechanical effects of Δ9-Tetrahydrocannabinol (THC) and cannabidiol (CBD), the major constituents of cannabis, in a sprague dawley rat achilles tendon surgical repair model: a pilot study. Am. J. Sports Med. 49 (9), 2522–2527. 10.1177/03635465211016840 34097540

[B195] Stith S. S. Li X. Diviant J. P. Brockelman F. C. Keeling K. S. Hall B. (2020). The effectiveness of inhaled cannabis flower for the treatment of agitation/irritability, anxiety, and common stress. J. Cannabis Res. 2 (1), 47. 10.1186/s42238-020-00051-z 33526145 PMC7819324

[B196] Stolar O. Hazan A. Vissoker R. E. Kishk I. A. Barchel D. Lezinger M. (2022). Medical cannabis for the treatment of comorbid symptoms in children with autism spectrum disorder: an interim analysis of biochemical safety. Front. Pharmacol. 13, 977484. 10.3389/fphar.2022.977484 36249785 PMC9559854

[B197] Tang Y. Tonkovich K. L. Rudisill T. M. (2022). The effectiveness and safety of cannabidiol in non-seizure-related indications: a systematic review of published randomized clinical trials. Pharm. Med. 36 (6), 353–385. 10.1007/s40290-022-00446-8 36271316 PMC9708636

[B198] Tavares P. Barrett J. Hogg-Johnson S. Ho S. Corso M. Batley S. (2020). Prevalence of low back pain, pelvic girdle pain, and combination pain in a postpartum Ontario population. J. Obstet. Gynaecol. Can. 42 (4), 473–480. 10.1016/j.jogc.2019.08.030 31864910

[B199] Taylor L. Gidal B. Blakey G. Tayo B. Morrison G. (2018). A phase I, randomized, double-blind, placebo-controlled, single ascending dose, multiple dose, and food effect trial of the safety, tolerability and pharmacokinetics of highly purified cannabidiol in healthy subjects. CNS Drugs 32 (11), 1053–1067. 10.1007/s40263-018-0578-5 30374683 PMC6223703

[B200] Thomas D. Frascella J. Hall T. Smith W. Compton W. Koroshetz W. (2015). Reflections on the role of opioids in the treatment of chronic pain: a shared solution for prescription opioid abuse and pain. J. Intern Med. 278 (1), 92–94. 10.1111/joim.12345 25556772 PMC4964933

[B201] Thompson A. L. Grenald S. A. Ciccone H. A. BassiriRad N. Niphakis M. J. Cravatt B. F. (2020). The endocannabinoid system alleviates pain in a murine model of cancer-induced bone pain. J. Pharmacol. Exp. Ther. 373 (2), 230–238. 10.1124/jpet.119.262337 32054717 PMC7160863

[B202] Toomey M. (2008). Gender differences in pain: does X = Y? AANA J. 76 (5), 355–359. 18947163

[B203] Treede R.-D. Rief W. Barke A. Aziz Q. Bennett M. I. Benoliel R. (2015). A classification of chronic pain for ICD-11. Pain 156 (6), 1003–1007. 10.1097/j.pain.0000000000000160 25844555 PMC4450869

[B204] Treede R. D. Rief W. Barke A. Aziz Q. Bennett M. I. Benoliel R. (2019). Chronic pain as a symptom or a disease: the IASP classification of chronic pain for the international classification of diseases (ICD-11). Pain 160 (1), 19–27. 10.1097/j.pain.0000000000001384 30586067

[B205] Trotti A. Colevas A. D. Setser A. Rusch V. Jaques D. Budach V. (2003). CTCAE v3.0: development of a comprehensive grading system for the adverse effects of cancer treatment. Semin. Radiat. Oncol. 13 (3), 176–181. 10.1016/S1053-4296(03)00031-6 12903007

[B206] Turk D. C. Dworkin R. H. Allen R. R. Bellamy N. Brandenburg N. Carr D. B. (2003). Core outcome domains for chronic pain clinical trials: IMMPACT recommendations. Pain 106 (3), 337–345. 10.1016/j.pain.2003.08.001 14659516

[B207] Turk D. C. Dworkin R. H. Revicki D. Harding G. Burke L. B. Cella D. (2008). Identifying important outcome domains for chronic pain clinical trials: an IMMPACT survey of people with pain. Pain 137 (2), 276–285. 10.1016/j.pain.2007.09.002 17937976

[B208] Turk D. C. Fillingim R. B. Ohrbach R. Patel K. V. (2016). Assessment of psychosocial and functional impact of chronic pain. J. Pain 17 (9 Suppl. l), T21–T49. 10.1016/j.jpain.2016.02.006 27586830

[B209] Turnbull A. Sculley D. Santos D. Maarj M. Chapple L. Girones X. (2022). Emerging tools to capture self-reported acute and chronic pain outcome in children and adolescents: a literature review. Med. Sci. (Basel) 10 (1), 6. 10.3390/medsci10010006 35225940 PMC8884018

[B210] Ulucay C. Ozler T. Guven M. Akman B. Kocadal A. O. Altintas F. (2013). Etiology of coxarthrosis in patients with total hip replacement. Acta Orthop. Traumatol. Turc 47 (5), 330–333. 10.3944/aott.2013.3103 24164942

[B211] Urech A. Krieger T. Moseneder L. Biaggi A. Vincent A. Poppe C. (2019). A patient post hoc perspective on advantages and disadvantages of blended cognitive behaviour therapy for depression: a qualitative content analysis. Psychother. Res. 29 (8), 986–998. 10.1080/10503307.2018.1430910 29385964

[B212] Valenzano K. J. Tafesse L. Lee G. Harrison J. E. Boulet J. M. Gottshall S. L. (2005). Pharmacological and pharmacokinetic characterization of the cannabinoid receptor 2 agonist, GW405833, utilizing rodent models of acute and chronic pain, anxiety, ataxia and catalepsy. Neuropharmacology 48 (5), 658–672. 10.1016/j.neuropharm.2004.12.008 15814101

[B213] van de Donk T. Niesters M. Kowal M. A. Olofsen E. Dahan A. van Velzen M. (2019). An experimental randomized study on the analgesic effects of pharmaceutical-grade cannabis in chronic pain patients with fibromyalgia. PAIN 160 (4), 860–869. 10.1097/j.pain.0000000000001464 30585986 PMC6430597

[B214] van den Hoogen N. J. Harding E. K. Davidson C. E. D. Trang T. (2021). Cannabinoids in chronic pain: therapeutic potential through microglia modulation. Front. Neural Circuits 15, 816747. 10.3389/fncir.2021.816747 35069129 PMC8777271

[B215] Vaughn S. E. Strawn J. R. Poweleit E. A. Sarangdhar M. Ramsey L. B. (2021). The impact of marijuana on antidepressant treatment in adolescents: clinical and pharmacologic considerations. J. Pers. Med. 11 (7), 615. 10.3390/jpm11070615 34209709 PMC8307883

[B216] Vazquez-Leon P. Miranda-Paez A. Chavez-Reyes J. Allende G. Barragan-Iglesias P. Marichal-Cancino B. A. (2021). The periaqueductal gray and its extended participation in drug addiction phenomena. Neurosci. Bull. 37 (10), 1493–1509. 10.1007/s12264-021-00756-y 34302618 PMC8490541

[B217] Vigli D. Cosentino L. Pellas M. De Filippis B. (2021). Chronic treatment with cannabidiolic acid (CBDA) reduces thermal pain sensitivity in Male mice and rescues the hyperalgesia in a mouse model of Rett syndrome. Neuroscience 453, 113–123. 10.1016/j.neuroscience.2020.09.041 33010341

[B218] Vincent J. I. MacDermid J. C. King G. J. W. Grewal R. Lalone E. (2019). Establishing the psychometric properties of 2 self-reported outcome measures of elbow pain and function: a systematic review. J. Hand Ther. 32 (2), 222–232. 10.1016/j.jht.2018.07.004 30587433

[B219] Volkow N. D. Baler R. D. Compton W. M. Weiss S. R. (2014). Adverse health effects of marijuana use. N. Engl. J. Med. 370 (23), 2219–2227. 10.1056/NEJMra1402309 24897085 PMC4827335

[B220] Vowles K. E. McEntee M. L. Julnes P. S. Frohe T. Ney J. P. van der Goes D. N. (2015). Rates of opioid misuse, abuse, and addiction in chronic pain: a systematic review and data synthesis. Pain 156 (4), 569–576. 10.1097/01.j.pain.0000460357.01998.f1 25785523

[B221] Wang X. Qiu Q. Shen Z. Yang S. Shen X. (2023). A systematic review of interpersonal psychotherapy for postpartum depression. J. Affect Disord. 339, 823–831. 10.1016/j.jad.2023.07.067 37459968

[B222] Weizman L. Dayan L. Brill S. Nahman-Averbuch H. Hendler T. Jacob G. (2018). Cannabis analgesia in chronic neuropathic pain is associated with altered brain connectivity. Neurology 91 (14), e1285–e1294. 10.1212/WNL.0000000000006293 30185448 PMC6177269

[B223] Whiting P. F. Wolff R. F. Deshpande S. Di Nisio M. Duffy S. Hernandez A.V. (2015). Cannabinoids for medical use: a systematic review and meta-analysis. JAMA 313 (24), 2456–2473. 10.1001/jama.2015.6358 26103030

[B224] Who W. H. O. (2004). International statistical classification of diseases and related health problems: alphabetical index. Geneva: World Health Organization.

[B225] Wijnhoven H. A. de Vet H. C. Smit H. A. Picavet H. S. (2006). Hormonal and reproductive factors are associated with chronic low back pain and chronic upper extremity pain in women--the MORGEN study. Spine (Phila Pa 1976) 31 (13), 1496–1502. 10.1097/01.brs.0000220706.96724.76 16741461

[B226] Xu J. Tang Y. Xie M. Bie B. Wu J. Yang H. (2016). Activation of cannabinoid receptor 2 attenuates mechanical allodynia and neuroinflammatory responses in a chronic post-ischemic pain model of complex regional pain syndrome type I in rats. Eur. J. Neurosci. 44 (12), 3046–3055. 10.1111/ejn.13414 27717112

[B227] Yamaori S. Ebisawa J. Okushima Y. Yamamoto I. Watanabe K. (2011). Potent inhibition of human cytochrome P450 3A isoforms by cannabidiol: role of phenolic hydroxyl groups in the resorcinol moiety. Life Sci. 88 (15-16), 730–736. 10.1016/j.lfs.2011.02.017 21356216

[B228] Yamaori S. Okushima Y. Yamamoto I. Watanabe K. (2014). Characterization of the structural determinants required for potent mechanism-based inhibition of human cytochrome P450 1A1 by cannabidiol. Chem. Biol. Interact. 215, 62–68. 10.1016/j.cbi.2014.03.007 24667653

[B229] Yasaei R. Peterson E. Saadabadi A. (2022). Chronic pain syndrome. *StatPearls*. (Treasure Isl. (FL)). Available online at: https://pubmed.ncbi.nlm.nih.gov/29262143/ . 29262143

[B230] Yong R. J. Mullins P. M. Bhattacharyya N. (2022). Prevalence of chronic pain among adults in the United States. PAIN 163 (2), e328–e332. 10.1097/j.pain.0000000000002291 33990113

[B231] Zamarripa C. A. Spindle T. R. Surujunarain R. Weerts E. M. Bansal S. Unadkat J. D. (2023). Assessment of orally administered Δ9-Tetrahydrocannabinol when coadministered with cannabidiol on Δ9-Tetrahydrocannabinol pharmacokinetics and pharmacodynamics in healthy adults: a randomized clinical trial. JAMA Netw. Open 6 (2), e2254752. 10.1001/jamanetworkopen.2022.54752 36780161 PMC9926328

[B232] Zeraatkar D. Cooper M. A. Agarwal A. Vernooij R. W. M. Leung G. Loniewski K. (2022). Long-term and serious harms of medical cannabis and cannabinoids for chronic pain: a systematic review of non-randomised studies. BMJ Open 12 (8), e054282. 10.1136/bmjopen-2021-054282 35926992 PMC9358949

[B233] Zhang J. Hoffert C. Vu H. K. Groblewski T. Ahmad S. O'Donnell D. (2003). Induction of CB2 receptor expression in the rat spinal cord of neuropathic but not inflammatory chronic pain models. Eur. J. Neurosci. 17 (12), 2750–2754. 10.1046/j.1460-9568.2003.02704.x 12823482

[B234] Zhang L. Kline R. H. t. McNearney T. A. Johnson M. P. Westlund K. N. (2014). Cannabinoid receptor 2 agonist attenuates pain related behavior in rats with chronic alcohol/high fat diet induced pancreatitis. Mol. Pain 10, 66. 10.1186/1744-8069-10-66 25403433 PMC4242547

[B235] Zhang H. Bi Y. Hou X. Lu X. Tu Y. Hu L. (2021a). The role of negative emotions in sex differences in pain sensitivity. Neuroimage 245, 118685. 10.1016/j.neuroimage.2021.118685 34740794

[B236] Zhang L. Wang J. Niu C. Zhang Y. Zhu T. Huang D. (2021b). Activation of parabrachial nucleus - ventral tegmental area pathway underlies the comorbid depression in chronic neuropathic pain in mice. Cell Rep. 37 (5), 109936. 10.1016/j.celrep.2021.109936 34731609 PMC8578703

[B237] Zieglgansberger W. Brenneisen R. Berthele A. Wotjak C. T. Bandelow B. Tolle T. R. (2022). Chronic pain and the endocannabinoid system: smart lipids - a novel therapeutic option? Med. Cannabis Cannabinoids 5 (1), 61–75. 10.1159/000522432 35702403 PMC9149512

