# Supplementary material for: Full-spectrum cannabis extracts for women with chronic pain syndromes: a real-life retrospective report of multi-symptomatic benefits after treatment with individually tailored dosage schemes
Source: Front Pharmacol. 2025 Nov 20;16:1538518. doi: 10.3389/fphar.2025.1538518 (PMC12675365; doi:10.3389/fphar.2025.1538518)
Supplement: Supplementary file 1 [file DataSheet2.pdf]

# Evaluation of the therapeutic effects of Cannabis extracts over Chronic Pain symptoms

SURVEY RELEVANCE:

First of all, THANK YOU for taking the time to answer this questionnaire. hronic pain has varied causes and presents in different ways for each person, just as Cannabis extracts vary in composition. Because of this, your help in understanding the results of using Cannabis extracts for Chronic pain treatment is very important to us. Your answers can help us improve the use of Cannabis for you and plenty of other patients who suffer from this condition. Your participation in this research is fundamental!

PEOPLE WHO SHOULD ANSWER THE SURVEY:

Ideally, the survey should be answered by the patient themself or, if they are unable to do so, by the person who lives with the patient and spends most of the time with them to get the most accurate information.

GUIDELINES FOR FILLING THE SURVEY:

We estimate it takes around 40 minutes for you to finish this survey but it must be answered carefully, so feel free to take breaks between questions or sections. Answers are recorded automatically so you can close the page and pick up from where you stopped at any moment. We kindly ask you, however, to finish filling this survey in a week after you begin, at the most. If you have any questions about this questionnaire, you can reach us through email (contato@neurovinci.com.br) or Whatsapp ((48) 98813-2000).

Please read the document below and, if you agree to participate, check the box in the bottom to continue answering

Thank you for helping us improve the quality of life of so many people who suffer from Chronic Pain!

* Indica uma pergunta obrigatória

## General Data

1. Name of the patient *

_______________________

2. Name of the person answering the survey: *

_______________________________

3. Relationship with the patient *

*Marcar apenas uma oval.*

- ( ) Father
- ( ) Mother
- ( ) Brother/Sister
- ( ) Main caregiver
- ( ) Prescribing doctor
- ( ) Patient themselves
- ( ) Other: _______________________________

4. Are you the person who spends most time daily with the patient? *

*Marcar apenas uma oval.*

- ( ) Yes
- ( ) No

5. Your phone *

_______________________________

6. Your email address *

_______________________________

7. Which is the cause of your chronic pain? *

*Marcar apenas uma oval.*

- Fibromialgia
- Muscular pain
- Dystonia
- Neuropathic pain (peripheral polyneuropathy, trigeminal neuralgy, radiculopathy)
- Articular pain (shoulders, hips, knees, ankles)
- Back pain (cervical, thoracic, lumbar)

8. Which doctor started and managed the treatment using Cannabis extract? *

*Marcar apenas uma oval.*

- Dr. Leandro
- Dr. Patrícia

9. When did the Cannabis treatment with either Dr. Patrícia or Dr. Leandro start? *

*Exemplo: 7 de janeiro de 2019*

10. Was the treatment with Dr. Patrícia or Dr. Leandro interrupted? *

*Marcar apenas uma oval.*

- Yes
- No

11. If the treatment was interrupted, please inform the approximate date of interruption.

*Exemplo: 7 de janeiro de 2019*

12. If the treatment was interrupted, please inform the reason for interruption.

*Marcar apenas uma oval.*

- Previous symptoms worsened.
- There were impactful side effects.
- Treatment was too expensive to maintain.
- Other: ______________________

13. Inform the biological sex of the patient. *

*Marcar apenas uma oval.*

- Female
- Male

14. Approximate weight of the patient at onset of treatment: *

______________________

15. Current weight of the patient or weight of patient at the treatment interruption: *

______________________

16. Please select the Cannabis extract used during treatment. *

*Marcar apenas uma oval.*

- CBD-rich Cannabis oil rich AMA+ME 1500mg
- THC-rich Cannabis oil AMA+ME 900mg (10%)
- THC- rich Cannabis oil AMA+ME 600mg (5%)
- CBD-rich Cannabis oil MALELI 3000mg (20%)
- CBD-rich Cannabis oil MALELI 1500mg (10%)
- THC- rich Cannabis oil MALELI 600mg (5%)
- THC- rich Cannabis oil MALELI 900mg (10%)
- Other: ______________________

17. Dose at the beginning of treatment *

______________________

18. Current dose (or last dose used) *

______________________

19. Support therapies and other activities during treatment *

*Marque todas que se aplicam.*

- [ ] Local heat or cooling
- [ ] Acupuncture
- [ ] Physiotherapy
- [ ] Stretching and massages
- [ ] Pilates
- [ ] Yoga
- [ ] Meditation
- [ ] Chiropractice
- [ ] None
- [ ] Other: ______________________

20. Had the patient used any kind of Cannabis-derived product before treatment with Dr. Patrícia or Dr. Leandro? *

*Marcar apenas uma oval.*

- Yes
- No

21. If the previous answer was "yes", please specify what was used, how it was used and for how long.

_______________________________________________
_______________________________________________
_______________________________________________
_______________________________________________
_______________________________________________

*Pular para a seção 3 (Evaluation of the Cannabis extract treatment results in Chronic pain patients )*

## Evaluation of the Cannabis extract treatment results in Chronic pain patients

GUIDELINES FOR ANSWERING SURVEY SESSIONS 1-14:

The next questions will measure the Cannabis treatment results in 10 separate groups of Chronic Pain-related symptoms. There are also 4 other groups of treatment impacts on the life and health of the patient and their family. All the 14 groups are as follows:

1) Pain episodes;
2) Sleep issues;
3) Persistent feeling of fatigue;
4) Motor impediments ;
5) Memory and cognitive problems;
6) Decreased libido or other sexual disfunctions;
7) Sadness, melancholy, and prostration;
8) Distress and irritability;
9) Anxiety crisis and/or panic attacks;
10) Inability to maintain a professional occupation;
11) Negative impacts over social and family relations;
12) Overall quality of life of the patient;
13) Overall quality of life of the family;
14) Adverse effects due to FCE treatment;

Most questions about symptoms present six options for answers. Check the one the best describes your situation concerning each symptom

Feel free to reach out if you have any doubts or questions!

Research team NeuroVinci/University of Brasilia(UnB)

## 1) Pain episodes

Objective: to evaluate the frequency and intensity of pain episodes since the patient presented symptoms of Chronic Pain.

22. What is your overall perception of treatment effect over those symptoms? *

*Marcar apenas uma oval.*

- The patient NEVER PRESENTED those symptoms since Chronic Pain started.
- Symptoms WORSENED SIGNIFICANTLY.
- Symptoms WORSENED MODERATELY.
- Symptoms DID NOT CHANGE.
- Symptoms IMPROVED MODERATELY.
- Symptoms IMPROVED SIGNIFICANTLY.

## 2) Sleep issues

Objective: to characterize insomnia episodes, difficulty sleeping, agitation during sleep and unwanted awakenings during the night since the patient presented symptoms of Chronic Pain.

23. What is your overall perception of treatment effect over those symptoms? *

*Marcar apenas uma oval.*

- The patient NEVER PRESENTED those symptoms since Chronic Pain started.
- Symptoms WORSENED SIGNIFICANTLY.
- Symptoms WORSENED MODERATELY.
- Symptoms DID NOT CHANGE.
- Symptoms IMPROVED MODERATELY.
- Symptoms IMPROVED SIGNIFICANTLY.

## 3) Persistent feeling of fatigue

Objective: to evaluate the frequency and intensity of periods described by the persistent feeling of fatigue or physical and mental exhaustion since the patient presented symptoms of Chronic Pain.

24. What is your overall perception of treatment effect over those symptoms? *

*Marcar apenas uma oval.*

- The patient NEVER PRESENTED those symptoms had symptoms of Chronic Pain.
- Symptoms WORSENED SIGNIFICANTLY.
- Symptoms WORSENED MODERATELY.
- Symptoms DID NOT CHANGE.
- Symptoms IMPROVED MODERATELY.
- Symptoms IMPROVED SIGNIFICANTLY.

## 4) Motor impediments

Objective: to evaluate the frequency and intensity of issues of motor control in every-day activities, such as getting up, walking, squatting, picking up objects, among others, since the patient presented symptoms of Chronic Pain.

25. What is your overall perception of treatment effects over those symptoms? *

*Marcar apenas uma oval.*

- The patient NEVER PRESENTED those symptoms since Chronic Pain started.
- Symptoms WORSENED SIGNIFICANTLY.
- Symptoms WORSENED MODERATELY.
- Symptoms DID NOT CHANGE.
- Symptoms IMPROVED MODERATELY.
- Symptoms IMPROVED SIGNIFICANTLY.

## 5) Inability to maintain a professional occupation

Objective: to characterize periods when Chronic Pain obstructed the patient's capacity to fulfill their professional occupation appropriately.

26. What is your overall perception of treatment effects over those symptoms? *

*Marcar apenas uma oval.*

- Patient is retired or had to retire due to pain.
- Patient does not have professional occupation or had to take a leave from work due to pain.
- Patient has NOT PRESENTED professional issues since Chronic Pain started.
- Symptoms WORSENED SIGNIFICANTLY.
- Symptoms WORSENED MODERATELY.
- Symptoms DID NOT CHANGE.
- Symptoms IMPROVED MODERATELY.
- Symptoms IMPROVED SIGNIFICANTLY.

## 6) Memory and cognitive problems

Objective: to evaluate memory lapses or issues and difficulties to understand or learn new things since the patient presented symptoms of Chronic Pain.

27. What is your overall perception of treatment effects over those symptoms? *

*Marcar apenas uma oval.*

- The patient NEVER PRESENTED those symptoms since Chronic Pain started.
- Symptoms WORSENED SIGNIFICANTLY.
- Symptoms WORSENED MODERATELY.
- Symptoms DID NOT CHANGE.
- Symptoms IMPROVED MODERATELY.
- Symptoms IMPROVED SIGNIFICANTLY.

## 7) Negative impacts over social and family relations

Objective: to characterize the overall degree of difficulty for maintaning adequate affective, social and familiar relations due to Chronic Pain.

28. What is your overall perception of treatment effects over those symptoms? *

*Marcar apenas uma oval.*

- The patient NEVER PRESENTED those symptoms since Chronic Pain started.
- Symptoms WORSENED SIGNIFICANTLY.
- Symptoms WORSENED MODERATELY.
- Symptoms DID NOT CHANGE.
- Symptoms IMPROVED MODERATELY.
- Symptoms IMPROVED SIGNIFICANTLY.

## 8) Decreased libido or other sexual disfunctions

Objective: to characterize the lack of interest in sex, lack of libido, difficulty feeling sexual pleasure, erectile disfunction and/or other forms of sexual disfunction since the patient presented symptoms of Chronic Pain.

29. What is your overall perception of treatment effects over those symptoms? *

*Marcar apenas uma oval.*

- The patient NEVER PRESENTED those symptoms since Chronic Pain started.
- Symptoms WORSENED SIGNIFICANTLY.
- Symptoms WORSENED MODERATELY.
- Symptoms DID NOT CHANGE.
- Symptoms IMPROVED MODERATELY.
- Symptoms IMPROVED SIGNIFICANTLY.

## 9) Sadness, melancholy and prostration

Objective: to characterize episodes of sadness, melancholy and prostration since the patient presented symptoms of Chronic Pain.

30. What is your overall perception of treatment effects over those symptoms? *

*Marcar apenas uma oval.*

- The patient NEVER PRESENTED those symptoms since Chronic Pain started.
- Symptoms WORSENED SIGNIFICANTLY.
- Symptoms WORSENED MODERATELY.
- Symptoms DID NOT CHANGE.
- Symptoms IMPROVED MODERATELY.
- Symptoms IMPROVED SIGNIFICANTLY.

## 10) Distress and irritability

Objective: to characterize episodes of distress and irritability since the patient presented symptoms of Chronic Pain.

31. What is your overall perception of treatment effects over those symptoms? *

*Marcar apenas uma oval.*

- The patient NEVER PRESENTED those symptoms since Chronic Pain started.
- Symptoms WORSENED SIGNIFICANTLY.
- Symptoms WORSENED MODERATELY.
- Symptoms DID NOT CHANGE.
- Symptoms IMPROVED MODERATELY.
- Symptoms IMPROVED SIGNIFICANTLY.

## 11) Anxiety crises and/or panic attacks

Objective: to characterize anxiety and/or panic attack episodes since the patient presented symptoms of Chronic Pain.

32. What is your overall perception of treatment effects over those symptoms? *

*Marcar apenas uma oval.*

- The patient NEVER PRESENTED those symptoms since Chronic Pain started.
- Symptoms WORSENED SIGNIFICANTLY.
- Symptoms WORSENED MODERATELY.
- Symptoms DID NOT CHANGE.
- Symptoms IMPROVED MODERATELY.
- Symptoms IMPROVED SIGNIFICANTLY.

## 12) Quality of life of the patient

Objective: to characterize the overall perception of well-being and quality of life of the patient before and after they started treatment for Chronic Pain.

33. Please describe in detail how was the quality of life of the family before and after treatment. *

_______________________________________________
_______________________________________________
_______________________________________________
_______________________________________________
_______________________________________________

34. What is your overall perception of treatment effects over those symptoms? *

*Marcar apenas uma oval.*

- The patient NEVER PRESENTED those symptoms since Chronic Pain started.
- Symptoms WORSENED SIGNIFICANTLY.
- Symptoms WORSENED MODERATELY.
- Symptoms DID NOT CHANGE.
- Symptoms IMPROVED MODERATELY.
- Symptoms IMPROVED SIGNIFICANTLY.

## 14) Quality of life of the family

Objective: to characterize the overall perception of well-being and quality of life of the family before and after they started treatment for Chronic Pain.

35. Please describe in detail how was the quality of life of the family before and after treatment. *

_______________________________________________

_______________________________________________

_______________________________________________

_______________________________________________

_______________________________________________

36. What is your overall perception of treatment effects over those symptoms? *

*Marcar apenas uma oval.*

- ◯ The patient NEVER PRESENTED those symptoms since Chronic Pain started.
- ◯ Symptoms WORSENED SIGNIFICANTLY.
- ◯ Symptoms WORSENED MODERATELY.
- ◯ Symptoms DID NOT CHANGE.
- ◯ Symptoms IMPROVED MODERATELY.
- ◯ Symptoms IMPROVED SIGNIFICANTLY.

## 13) Side effects

Objective: to characterize untoward effects that may be attributed to the Cannabis extract treatment.

Objetivo: avaliar a ocorrência, frequência e intensidade de efeitos indesejados que possam ser atribuídos ao tratamento com extrato de cannabis.

37. Check the boxes below corresponding to the adverse effects that occurred since treatment started. For each effect, do not forget to report the intensity (mild, moderate or severe) and when it occurred (only in the beggining or still ongoing). *

*Marque todas que se aplicam.*

| | Did not occur | Mild | Moderate | Severe | Only in the start of treatment | Still ongoing |
|---|---|---|---|---|---|---|
| **Sleepiness** | ☐ | ☐ | ☐ | ☐ | ☐ | ☐ |
| **Insomnia** | ☐ | ☐ | ☐ | ☐ | ☐ | ☐ |
| **Headaches** | ☐ | ☐ | ☐ | ☐ | ☐ | ☐ |
| **Dizziness** | ☐ | ☐ | ☐ | ☐ | ☐ | ☐ |
| **Dizziness when standing up** | ☐ | ☐ | ☐ | ☐ | ☐ | ☐ |
| **Dry mouth** | ☐ | ☐ | ☐ | ☐ | ☐ | ☐ |
| **Coughs** | ☐ | ☐ | ☐ | ☐ | ☐ | ☐ |
| **Euphoria** | ☐ | ☐ | ☐ | ☐ | ☐ | ☐ |
| **Agitation** | ☐ | ☐ | ☐ | ☐ | ☐ | ☐ |
| **Anxiety** | ☐ | ☐ | ☐ | ☐ | ☐ | ☐ |
| **Unresponsiveness, inebriation (states associated to the "Cannabis high")).** | ☐ | ☐ | ☐ | ☐ | ☐ | ☐ |
| **Mental confusion** | ☐ | ☐ | ☐ | ☐ | ☐ | ☐ |
| **Depression** | ☐ | ☐ | ☐ | ☐ | ☐ | ☐ |
| **Nausea** | ☐ | ☐ | ☐ | ☐ | ☐ | ☐ |
| **Blurred vision** | ☐ | ☐ | ☐ | ☐ | ☐ | ☐ |
| **Ataxia / motor discoordination** | ☐ | ☐ | ☐ | ☐ | ☐ | ☐ |
| **Tachycardia** | ☐ | ☐ | ☐ | ☐ | ☐ | ☐ |

| | | | | | | |
|---|---|---|---|---|---|---|
| Diarrhea | ☐ | ☐ | ☐ | ☐ | ☐ | ☐ |
| Constipation | ☐ | ☐ | ☐ | ☐ | ☐ | ☐ |
| Red eyes | ☐ | ☐ | ☐ | ☐ | ☐ | ☐ |

38. If you checked "Other" above, please explain which effects you had in detail.

Este conteúdo não foi criado nem aprovado pelo Google.

Google Formulários
