# Supplementary material for: Full-spectrum cannabis extracts for women with chronic pain syndromes: a real-life retrospective report of multi-symptomatic benefits after treatment with individually tailored dosage schemes
Source: Front Pharmacol. 2025 Nov 20;16:1538518. doi: 10.3389/fphar.2025.1538518 (PMC12675365; doi:10.3389/fphar.2025.1538518)
Supplement: Supplementary file 2 [file DataSheet4.pdf]

Certificates of cannabinoid analyses - AMAME association

Cromatograma e resultados

Detalhes da Análise

Nome	AMA+ME Lote: AMA002	Data	18/06/2021

ABRACE

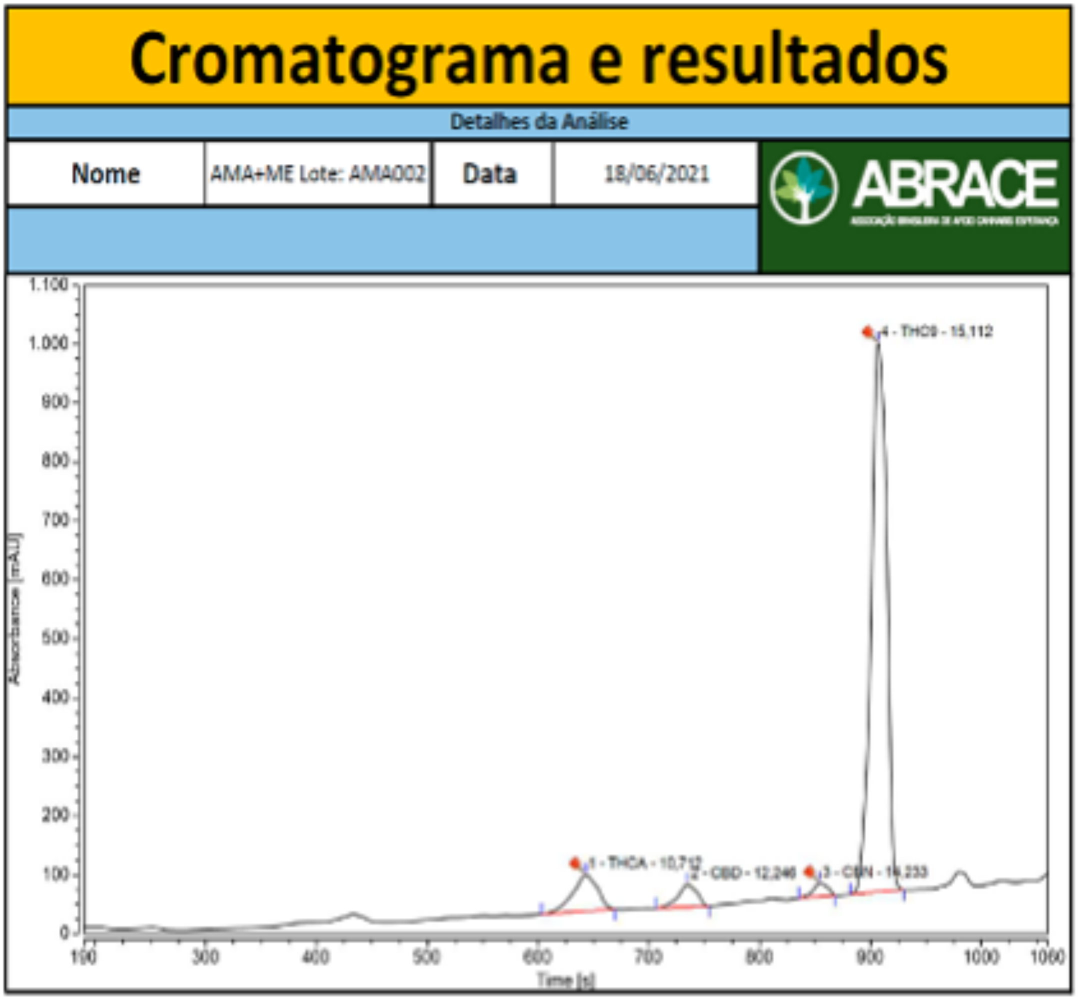

Resultados

Substância	Concentração (mg/g)	% *	Proporção THC/CBD
THC-A	30,18	3,0%	22,5:1
THC	399,60	40,0%	
CBD-A	0,00	0,0%	
CBD	17,75	1,8%	
CBN	3,64	0,4%	
THC total	429,8	43,0%	
CBD total	17,8	1,8%	
Canabinóides total	447,5	45,1%	

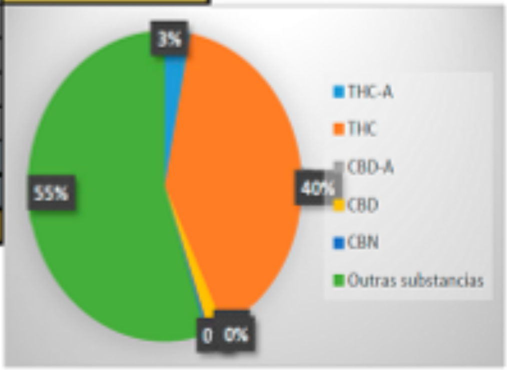

Farmacêutico Responsável Técnico

THE HIGHER COMMITMENT ANALYTICAL LAB

The Higher Commitment Analytical Lab
440 Lower Grass Valley Road, Suite A
Nevada City, CA 95959

(530) 264-7789
www.thehighercommitment.com
Lic# C8-0000117-LIC

QA Testing

RSO

Sample ID: THCA22021606-01
Strain: RSO
Matrix: Concentrates & Extracts
Type: RSO
Sample Size: 1 units; Batch:

Received: 02/16/2022
Completed: 02/21/2022
Batch#:

Client
[redacted]

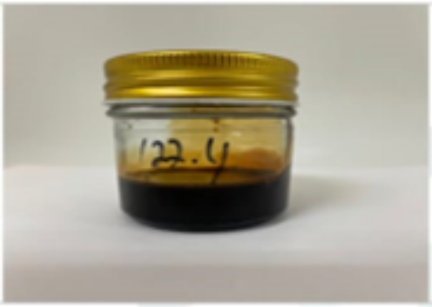

Summary

Test	Date Tested	Method	Result
Batch			Pass
Cannabinoids	02/17/2022		Complete
Residual Solvents	02/18/2022	OG-03; GCMS	Pass
Density			*g per mL

Cannabinoids

Complete

3.724%	78.497%	85.050%
Total THC	Total CBD	Total Cannabinoids
**NT**	**Not Tested**	**Not Tested**
Moisture	Water Activity	Foreign Matter

Analyte	LOD	LOQ	Result	Result
	mg/g	mg/g	mg/g	%
THCa	0.25	0.50	1.10	0.110
Δ9-THC	0.25	0.50	36.27	3.627
Δ8-THC	0.25	0.50	ND	ND
THCV	0.25	0.50	ND	ND
CBDa	0.25	0.50	304.72	30.472
CBD	0.25	0.50	517.73	51.773
CBN	0.25	0.50	0.79	0.079
CBGa	0.25	0.50	3.60	0.360
CBG	0.25	0.50	0.69	0.069
CBC	0.25	0.50	23.65	2.365
**Total CBD**			**784.970 mg/g**	**78.497%**
**Total THC**			**37.237 mg/g**	**3.724%**
**Total**			**850.497 mg/g**	**85.050%**

Total THC = THCa * 0.877 + Δ9-THC; Total CBD = CBDa * 0.877 + CBD
LOQ = Limit of Quantitation; The reported result is based on a sample weight with the applicable moisture content for that sample; Unless otherwise stated all quality control samples performed within specifications established by the Laboratory. Test method: OG-01 - Cannabinoids by HPLC.

Lori Katrencik
Lab Director
02/21/2022

Kyle Nesbitt
Lab Manager
02/21/2022

Confident Cannabis
All Rights Reserved
support@confidentcannabis.com
(866) 506-5866
www.confidentcannabis.com

ND=Not Detected, NR=Not Reported, LOQ=Limit of Quatitation. Test method for moisture: GLT-02 - Determination of Percent Moisture. Test method for water activity: GLT-03 - Determination of Water Activity. This product has been tested by The Higher Commitment Analytical Lab using valid testing methodologies and a quality system as required by state law. All LQC samples were performed and met the prescribed acceptance criteria in 16 CCR section 5730, pursuant to 16 CCR section 5726(e)(13). Values reported relate only to the product tested. The Higher Commitment Analytical Lab makes no claims as to the efficacy, safety or other risks associated with any detected or non-detected levels of any compounds reported herein. This Certificate shall not be reproduced except in full, without the written approval of The Higher Commitment Analytical Lab.
