# Supplementary material for: Full-spectrum cannabis extracts for women with chronic pain syndromes: a real-life retrospective report of multi-symptomatic benefits after treatment with individually tailored dosage schemes
Source: Front Pharmacol. 2025 Nov 20;16:1538518. doi: 10.3389/fphar.2025.1538518 (PMC12675365; doi:10.3389/fphar.2025.1538518)
Supplement: Supplementary file 3 [file DataSheet3.pdf]

SUPPLEMENTARY MATERIAL 3: Open-ended Responses on Quality of Life.

The two multiple-choice questions addressing the impact of FCE treatment on quality of life were complemented by corresponding open-ended inquiries on the same subject:
a) "Describe in detail, in your own words, what the patient's overall quality of life was like before the treatment and what it came to be like after the treatment."
b) " Describe in detail, in your own words, what the family's overall quality of life was like before the treatment and what it came to be like after the treatment."
The open-ended responses, initially provided in Brazilian Portuguese, underwent careful translation into English and are presented in full below.

1. (1f)
a. Before, she used to have no disposition to do simple household tasks, always very down and apathetic. The patient gained more energy to leave the house, like going to the market, talking to people as she always did.
b. Before she was apathetic, with many pains, down, discouraged. As soon as she started the treatment, she returned to her previous self, talking to everyone, and leaving the house. Today, we have to monitor her exits because she doesn't stop anymore, she is doing very well.

2. (2f*)
a. There was moderate improvement in the quality of life, not much due to the rare erythromelalgia syndrome.
b. There was moderate improvement in the family's quality of life.

3. (3f)
a. Before starting treatment, the pain was more intense. After starting treatment, the pain decreased.
b. It was 8 years of great suffering with several treatments such as medication, applications, physiotherapy, and other procedures, and my family suffering along with me, because none of these procedures worked. Then came sadness, despair, anxiety, frustration for what was happening to me. That was when I decided to seek this new treatment (cannabis oil) and it has been a real miracle in my life and my family's life.

4. (4f)
a. Considerable improvement with treatment.
b. It used to be a life with sleepless nights, forgetfulness, and seizures.

5. (5f)
a. Regarding the pain crises, before they were frequent, intense pain to the point where I couldn't do daily activities. During the crisis, the pain was incapacitating, so the irritability, for not being able to do everyday things, was very intense! Not to mention the fear of feeling sick while I was with my children, driving! After I started treatment, the pain crises decreased considerably, usually the pain comes during the menstrual period, and now the intensity of the pain is lower. I feel calmer today, more relaxed, and the memory issue, I notice that it has improved a lot! I am very grateful to be treated and accompanied by Dr. P.M.! Today I feel much better and more relaxed.
b. Both me and my family used to be tense during the pain crises because I was very afraid of having a syncope or not being able to stand up, like in other crises, afraid of them turning my head upside down and doing a series of tense and painful procedures, that left me worse than I was before. Today, I have more awareness of when the pain comes, take the cannabis medication to cut the pain and with the decrease in crises, I feel calmer, consequently, the whole family is calmer and lighter.

6. (6f)

a. Before taking the cannabis medication, I used to spend two days at home and one day in the hospital due to a lot of pain in my head, neck, spine, arm, etc. I underwent brain surgery due to "Chiari malformation" and it took me a long time to recover from the trauma. I had a lot of muscular pain, bursitis, and tendinitis in my left arm, tendinitis in my right arm, hernia in my lower back, knee wear and tear, tendinitis in my hands, and several other diseases. When I started taking cannabis, all these pains were reduced from 10 to 4. I only had to go to the hospital occasionally, with intervals of 2 to 3 months. I even spent 6 months without going to the hospital, it was a completely different life. But I lost my job due to so many sick leave certificates, just in the second month of taking cannabis. I lost my health insurance, and the doctors at the public medical center refused to follow Dr. P.M.'s prescription. They still help me with other medications, but I am ashamed and unable to buy cannabis oil. Today, the headaches have returned, but less intense. New pains have emerged and added to the existing ones, I live on "musculare" (cyclobenzaprine), "amitriptyline", "sertraline", and other pain medications. But I would like to volunteer for tests and start taking cannabis oil with cannabidiol again, because today I have life, but no quality of life, without the cannabis medicine, I live 24 hours in pain.
b. Before the cannabis treatment, it was only pain and hospital, I didn't do anything at home, I was just a burden. I lived well for two and a half years, which was the period I took the medication (cannabis extract). But today, I always live with pain.

7. (7f)
a. I have been suffering from Fibromyalgia since I was 12 years old, which was triggered by the start of my menstrual cycle. But I was only diagnosed at age 20. Before that, I was treated as having various other diseases, including rheumatic diseases, until I was diagnosed with Fibromyalgia. Since I was 12, I didn't know what it was like to sleep and wake up without strong pain. The pain was part of my daily life. I had worse crises that left me practically bedridden at least once a month. With the arrival of my daughters, things got worse. I reached the point where I couldn't handle daily tasks, had to miss work, and couldn't play or go out with my family anymore. Over time, I started having anxiety attacks, and a few years ago, I fell into depression. There came a point where I no longer saw light at the end of the tunnel. When I started treatment with cannabis, I had no side effects, and Dr. P.M. informed me that it would take almost a month to feel the effects. I started on a Thursday, and on Saturday, I started cleaning the house in a frenzy, but I didn't even realize what it was. I was able to perform all activities without feeling any pain, and only at the end of the day did I realize that it was the result of the cannabis oil. Since then, I no longer know what it's like to feel pain. I haven't had any more crises. Not even headaches, which used to be constant. I stopped taking blood pressure medication and any other pain medication. Muscle relaxants are no longer part of my life. I don't even suffer from menstrual cramps anymore. Anxiety and depression disappeared. Today, I live a full life. And most importantly, without any pain.
b. Answer included in the previous description.

8. (8f)
a. Before the treatment, I would avoid participating in any event due to the pain. Since I avoided taking pain medication, I used hot packs and rest, especially in the winter. I avoided traveling and staying away from home due to the constant pain. Stretches, Pilates exercises, and the Busquet technique always relieved the pain as well. Currently, with the use of cannabis oil, I still feel discomfort in some joints (especially in the hips where I have a total prosthesis), but it's incomparable to the persistent pain before. I feel a lot of difference in the quality of life nowadays.

9. (9f*)
a. Muscle pain, insomnia, fatigue, cramps, pain in all joints, swelling in the face, jaw pain, anxiety, panic, restlessness, frequent headaches, blurred vision, pain in the soles of the feet, sensation of shocks in the neck.

10.(10f)

a. Before starting treatment with cannabis oil, I had unsatisfactory sleep, took a lot of medication, and this interfered with my bowel function. Currently, the quality of sleep has improved, there is a lower intake of medication, the bowel functions regularly and without pain.

b. The family's quality of life was affected by the symptoms I had because fibromyalgia affected my performance and mood. Currently, there has been an improvement because the treatment with cannabis oil allowed me to resume my daily activities and improved my mood.

11.(11f)

a. The quality of life improved significantly after the minimally invasive procedure with medication in the thoracic area, and with Cannabis, relaxation also contributed to pain relief. b. There were no changes in family quality of life.

12. (12f)

a) I used to feel terrible about the pain, but after starting treatment with CBD [cannabis oil], I felt 80% better. Currently, I feel good, but still have pain crises, about 60% improvement.

b) I couldn't walk daily due to the pain, but now I can do it a little bit more.

13. (13f)

a) I've been an oncology patient for 12 years, with breast cancer and bone metastasis. I had joint pain, cramps in various parts of the body, numbness in my left hand, lack of strength in my left arm and hand, insomnia... Since starting treatment, I've felt a great improvement in these symptoms. I can do domestic and personal activities that I couldn't do before.

b) There was a great improvement.

14. (14f)

a) Before, I was lethargic, without energy, but now I have more disposition.

b) The family is now less worried about the past symptoms.

15. (15f)

a) Before treatment, on days when the pain was severe, I avoided social activities, especially if they involved sitting or standing for long periods, because the pain and muscle stiffness would increase. My libido was reduced, leading to a negative impact on my marriage. On days when I had pain crises, my ability to come to conclusions was impaired, as well as my memory, attention, etc., hindering my work activities. I woke up several times at night and felt tired in the morning, even when taking medications like pregabalin, sertraline, Duloxetine#... the crises were more frequent. After starting treatment with cannabis oil, within a few weeks, there was a significant improvement in the quality of sleep, affective relationship, pain reduction, memory and concentration improvement. I was no longer using the conventional medications mentioned above. During the treatment period, I became more involved in social and leisure activities. The pain did not disappear, but it reduced to a lower intensity threshold that improved my quality of life. I could see how effective the treatment was when I went a month without the cannabis medication: within a few days, the pain crises returned, along with problems with memory, irritability, lack of concentration, joint stiffness, sadness, loss of libido, difficulty sleeping, waking up several times at night and leg agitation.

b) Before, I avoided hugs and sexual relations. I couldn't walk much and would sit for long periods, so I avoided outings. After using cannabis oil, my affective life improved, as did my socialization.

16. (16f)

a) Before, I avoided hugs and sexual relations. I couldn't walk much and would sit for long periods, so I avoided outings. After using cannabis oil, my affective life improved, as did my socialization.

a) Before the treatment with Cannabis oil, I had no quality of life, couldn't perform household tasks without suffering a lot, couldn't exercise regularly and with quality, couldn't take long showers standing up, felt very strong pains, in my body and head, 24 hours a day. After the Cannabis treatment, I can easily do all the household tasks, have more energy and sleep much better, without waking up at night with pain, greatly reduced the quantity of painkillers I take, exercise daily, including going to the gym, and have regained pleasures that I thought were lost, such as feeling the touch of a soft sheet on my skin, my sense of touch is much sharper now. My life has improved greatly and I am happy again.

b) According to my family members, now, with the Cannabis treatment, I am much happier and sociable.

17. (17f)

a) As I worked sitting down, I felt a lot of lower back, hip, and coccyx pain. I started treatment with cannabis and the pain went away, but I no longer sit for a long time as I am on medical leave due to shoulder surgery. When I return to work, I will be able to evaluate better.

18. (18f*)

a. For over 20 years, I suffered from chronic pain, fibromyalgia. I took all kinds of medication, underwent numerous treatments, used all sorts of devices, and went through a long list of doctors in various specialties. Nothing worked like cannabis oil. It was a turning point in my life. I am very grateful to everyone involved.

b. Everyone was involved in my complaints. Everyone was worried and looking for some kind of result, something new that could help, but nothing worked before cannabis.

19. (19f*)

a. Before treatment, I had no quality of life because constant chronic pain limited my abilities and the pleasure of living. Everything revolved around how to survive the pain. As I started treatment with cannabis, the improvement was gradual, returning my autonomy, allowing me to resume constant physical activities and the joy of living, and I did not have severe side effects as with conventional treatments.

b. Before treatment, the quality of life for my family was quite poor because my health condition required more assistance, generated concerns, and the coexistence was very stressful. After starting treatment, with my gain in quality of life, everyone around me also ends up benefiting with better and healthier coexistence.

20. (20f*)

a. I sought treatment with cannabis oil for presenting osteoarthritis with chronic joint pain. There was great improvement with treatment, but I also started treatment with a psychiatrist for bipolar disorder and she asked me to stop using THC and CBD.

b. My family relationship was and still is good, we did not notice any significant changes.

21. (21f*)

a. Before the treatment, I would have consecutive days of pain and couldn't establish a rhythm in my activities. Now I'm much better and my life is a little more productive.

b. For those who live with someone who has chronic pain, it's a heavy burden because the person is never able to go out, have fun, or do important daily activities, and this affects those closest to them. After the treatment, this has significantly changed and consequently improved the quality of relationships.

22. (22f)

a. I used to sleep very little, or only with medication. Now I sleep better and without sleeping pills. I feel calmer, less anxious, more tolerant, and have more energy to do physical activity. I feel better.

b. I was always stressed, impatient, because people couldn't understand what I used to feel. I was always unwilling and in pain. My dream was to wake up one day without pain. I hope that I can at least alleviate these symptoms. I can't imagine my life without cannabis anymore. My family says that I have improved a lot in my relationship with them, even though I live alone. My partner left me and never understood. He said that I wasn't doing the previous treatments correctly. But of all the things I've done so far, it was with cannabis that I achieved the best results. This medicine should be provided by the government because many people don't have access to it.

23. (23f)
a. Stuck.
b. Well-being.

24. (24m*, this is the only male patient, his answer was not included in this analysis, therefore, it is omitted here.)

25. (25f)
a. Before: a lot of pain, motor limitation, discouragement, anguish. After: well-being, return of motor skills.
b. The family was upset before and now they are very happy.

26. (26f)
a. I used to have migraines 3 to 4 times a week, after the treatment I had only 1 or no crises per month due to the menstrual period.
b. I used to have migraines 3 to 4 times a week, after the treatment I had only 1 or no crises per month due to the menstrual period.

27. (27f)
a. When I went to see Dr. P.M., I felt very debilitated. Daily pain was limiting and I had lost a large part of my body's movement. I walked with a limp and at times was unable to turn in bed, shower, or dress myself. Household chores, walking, and getting up in the morning were all tasks I couldn't accomplish. The physical part debilitated me so much that other medications were no longer effective. Sleeping on my body, head, and face with pain, and anxiety turned nights into one of the most difficult times of the day. Deep sleep never happened for years. When I did manage to sleep, I had many nightmares, always related to some pain I was feeling. The anxiety part remains a little, but it's noticeable, seen through my eyes and by my close family members, that when I have a problem in the moment, I get nervous but it's quickly resolved and I don't dwell on it afterward. Before the cannabis medication, I would suffer for a week over very small problems, and it was very disturbing for many days in a row. Social interaction has significantly improved since I have been able to engage in activities that were not possible before. The fact that I feel socially more capable of carrying out tasks and no longer complaining about pain and lack of enthusiasm, as I often used to do, has increased my quality of life and desire for social interaction, which I no longer had before the treatment. Forgetfulness, frequent memory loss, lack of concentration, and mental confusion were all common before. However, today, I have returned to studying and feel much more capable of remembering things. I want to say that through the cannabis treatment, I have experienced significant improvements, and I feel that the treatment at the clinic is very humanized, with a focus on improving people's lives. I thank Dr. P.M. for training in this knowledge, which has brought me so many improvements in my quality of life as a patient.

b. Being married and having three children, I ended up overwhelming other family members. Tasks that I used to do had to be done by my husband, who worked outside the home a lot, and by my children. My relatives, including my parents, were always worried, but couldn't help much since they lived far away. They helped me so much with daily life in the house and to assist me in my difficulties with the disease at its most

difficult stage. Nowadays, I can do household tasks and assist each of them with their needs, school tasks, among others, and also enjoy leisure time with my family with a better quality of life. Another factor that helped me have more energy was that after starting to use the cannabis medication, I began to lose weight, which helped me have more physical energy to interact with others in even simple activities.

28. (28f)
a. With the treatment, I became less irritable, had muscular relaxation, and had more desire to do my activities, even with difficulties.

29. (29f)
a) I have suffered from chronic migraines since I was about 24 years old. Main triggers: stress, sleep deprivation, alcohol consumption, and premenstrual syndrome. I am currently 31 years old. It was about 7 years with crises that gradually increased in terms of frequency and intensity. Before resorting to Cannabis treatment (in December 2021) I was having an average of 20 days of pain per month (according to my migraine diary), ranging from moderate to severe pain. The intensity was usually higher in the 7-10 days of the menstrual period. No conventional medication relieved the pain completely. Sumatriptan only made the pain less intense. Over the 6 months of Cannabis treatment, the frequency and intensity of the crises decreased, also gradually, until it disappeared. In the first month of using Cannabis oil, the improvement was slightly significant. In the second and third months, I started having fewer days of pain (usually 10 days, concentrated before, during, and after the menstrual period). And in the last three months of using the oil, the intensity of the pain also decreased along with the frequency. June 2022 was my first month in 7 years without any migraine episode, not even mild. We are in October 2022. Since then, some isolated episodes of mild pain sometimes arise, usually during the menstrual period, which improve overnight or with conventional pain relievers. My quality of life, therefore, has significantly improved with the 6 months of Cannabis oil use (associated with the sport of surfing that I started practicing in the same period, and which I still practice today).
b) I live alone and did not meet my relatives (who live in another state) during the treatment period.

30. (30f*)
a) It's been 3 and a half years of headache that's not a migraine, as I used to believe. It has been diagnosed by another neurologist as hypnic headache. It's a headache that occurs every night around 3 or 4 am, and goes away after about 2 hours. Using all the standard migraine medications during the first 2 years did not improve my condition. Cannabis mainly improved my sleep, making it easier to go back to sleep after the pain stopped, and moderately helped with day-to-day activities, reducing the worry about how the next night would be. It also eliminated the need for the medication Amitriptyline, which was helping with this objective. b) There was no substantial change, the consequences were more individual.
