# Supplementary material for: Full-spectrum cannabis extracts for women with chronic pain syndromes: a real-life retrospective report of multi-symptomatic benefits after treatment with individually tailored dosage schemes
Source: Front Pharmacol. 2025 Nov 20;16:1538518. doi: 10.3389/fphar.2025.1538518 (PMC12675365; doi:10.3389/fphar.2025.1538518)
Supplement: Supplementary file 4 [file DataSheet1.pdf]

								Initial Dosage (after tritation)							Final Dosage (after posterior adjustments)						Cannabis oils used for treatment
Pain classification	Main CPS ICD	Case	Initial Dominnant Cannabinoid	Final Dominant Cannabinoid	General Outcome Score (GOS)	Persistent Moderate Side Effect	ICDs	Criteria for choosing initial doses	CBD (mg/kg/day) (x10-1)	THC (mg/kg/day) (x10-1)	CBD (mg/day)	THC (mg/day)	CBD:THC	Wgt (kg)	Criteria for choosing final doses	CBD (mg/kg/day) (x10-1)	THC (mg/kg/day) (x10-1)	CBD (mg/day)	THC (mg/day)	CBD:THC	
Inflamatory / mixed	Joint pain	4f	CBD	CBD	1,7	N	M25.5, G40	Patient presenting diffuse joint pain, history of epilepsy. No new episodes but presenting pre-episode symptoms even under levetiracetam use. CBD-rich extract was chosen for its pain and epilepsy alleviating capabilities. Low to intermediary dose was chosen given this is a low risk case.	7.26	1.81	45.00	11.25	04:01	59	Patient showed great pain improvement and complete absence of pre-episode symptoms. There was a epileptic event during dose reduction of levetiracetam, and so dose was adjusted to CBD-rich extract (75mg/day). Patient has not had other episodes and pain under control ever since.	12.71	3.18	75.00	18.75	04:01	CBD-rich Cannabis oil MALELI 3000mg (20%)
		13f	THC	THC	1,2	insomnia, headaches, dizziness, dry mouth, anxiety, constipation.	M.25.5, C50	Patient presenting metastatic breast cancer, diffuse pain (likely to be peripheral neuropathy post chemotherapy. Poor sleep quality and presenting nausea due to chemotherapy. Treatment consisted of intermediary doses of THC-rich extract given its pain alleviating and sleep improvement properties, besides alleviating nausea.	0.02	0.91	0.20	7.50	01:38	82	Despite some side effects when extract treatment started, patient has shown large improvement of the issues mentioned.	0.02	0.91	0.20	7.50	01:38	THC-rich Cannabis oil MALELI 900mg (10%)
		8f	CBD	CBD	1	N	M25.5	Patient with Chronic Pain in both hips, underwent bilateral arthroplasty10 years ago. Says she has poor sleep quality (smokes Cannabis for pain and insomnia and report some improvement in both issues). They also present systemic arterial hypertension, Diabetes mellitus, obesity.	5.86	0.28	37.50	1.80	21:01	64,00	Patient showed great improvement in all aspects previously mentioned. Treatment stretagy was maintained.	5.86	0.28	37.50	1.80	21:01	CBD-rich Cannabis oil AMA+ME 1500mg
	Coxartrose	20f	CBD	CBD	1,1	Dry mouth, excessive thirst.	M16, M25.5	Patient with Chronic Pain in both hips, underwent bilateral arthroplasty10 years ago. Says she has poor sleep quality (smokes Cannabis for pain and insomnia and report some improvement in both issues). They also present systemic arterial hypertension, Diabetes mellitus, obesity. Treatment chosen consists in low to intermediate CBD-rich extract given its antiinflammatory, ansiolitic and pain alleviating properties. Also, it can improve symptoms of plurimetabolic syndrome (glicemic improvement, reduction of appetite, weight loss). A low-dose, THC rich extract is added for sleep issues.	6.18	1.95	45.14	14.25	03:01	73,00	Patient showed great improvement in all aspects previously mentioned. Treatment stretagy was maintained.	6.18	1.95	45.14	14.25	03:01	CBD-rich Cannabis oil MALELI 1500mg (10%) / THC-rich Cannabis oil MALELI 600mg (5%)
	Migraine	6f	THC	THC	1,4	N	G43, Q07.0, M54.2	Patient presenting diffuse pain, particularly more intense in the lower back, all post operation to treat Arnold Chiari Syndrome. Also presented insomnia and emotional disturbances. Treatment chosen consisted in low doses of THC-rich extract given its pain alleviating, anti-inflammatory and ansiolitic properties.	0.02	0.75	0.12	4.50	01:38	63	Patient showed great improvement in all aspects previously mentioned. Treatment stretagy was maintained.	0.02	0.71	0.12	4.50	01:38	THC-rich Cannabis oil MALELI 900mg (10%)
		26f	CBD	CBD	1,4	N	G43	Patient presenting with migraine headaches and anxious mood states. Treatment consisted of intermediary doses of CBD-rich extract given its pain alleviating, anti-inflammatory and ansiolitic properties.	6.92	1.73	45.00	11.25	04:01	65	Patient showed great improvement in pain management. Treatment stretagy was mostly maintained with slight dose adjustment to account for the anxious states.	9.23	2.31	60.00	15.00	04:01	CBD-rich Cannabis oil MALELI 3000mg (20%)
		29f	CBD	CBD	1,3	N	G43	Patient presenting with migraine headaches and anxious mood states. Treatment consisted of intermediary doses of CBD-rich extract given its pain alleviating, anti-inflammatory and ansiolitic properties.	9.00	2.25	45.00	11.25	04:01	50	Patient showed great improvement in pain management. Treatment stretagy was maintained.	9.00	2.25	45.00	11.25	04:01	CBD-rich Cannabis oil MALELI 3000mg (20%)
		5f	CBD	CBD	0,9	N	G43	Patient presenting tension-related headaches with anxiety aspects. Treatment chosen consists in CBD- rich extract, given its ts antiinflammatory, ansiolitic and pain alleviating properties. Low to intermediary dose was chosen to prevent potential side effects (patient seems to be particularly sensitive to medication side effects in general).	6.82	0.33	37.50	1.80	21:01	57	Patient showed great improvement in pain and anxiety issues. Treatment strategy was maintained.	6.58	0.32	37.50	1.80	21:01	CBD-rich Cannabis oil MALELI 3000mg (20%)
		21f	THC	THC	0,7	N	G43	Patient diagnosed with Hodgkin's Lymphoma presenting many complaints related to the disease and chemotherapy treatment (loss of appetite, anxiety, insomnia, migraines and diffuse pain). Smokes Cannabis regularly (1-2 cigarrets/day on average). Treatment chosen consists in intermediary doses of THC-rich extract (patient most likely has high tolerance to THC).	0.09	3.37	0.43	16.50	01:38	56	Patient showed great improvement in clinical aspects after starting treatment with THC-rich extract. However they were not able to reduce the smoking of Cannabis and there was worsening of anxiety and depression. New treatment chosen consists of adding intermediary dose of CBD-rich extract to deal with these aspects.	0,27	0,11	15,16	6,31	2:1	CBD-rich Cannabis oil MALELI 3000mg (20%)
		30f	THC	CBD	0,4	N	G43	Patient diagnosed with chronic migraines and bad sleeping habits. Patient is already using CBD-rich extract without improvement in the symptoms mentioned. New treatment consists in adding a low to intermediary dose of THC-rich extract, only at night.	0.02	0.68	0.12	4.50	01:38	67	Patient showed great improvement in pain symptoms. There was improvement in sleep habits. Treatment strategy was maintained.	5.99	2.39	40.16	16.00	03:01	CBD-rich Cannabis oil MALELI 3000mg (20%) / THC-rich Cannabis oil MALELI 900mg (10%)
	Back Pain	11f	CBD	CBD	0,9	N	M.54	Patient presenting facetary pain in dorsal and cervical regions. Treatment chosen consists in low to intermediary doses of CBD-rich extract, given its antiinflammatory and pain alleviating properties.	6.43	1.61	45.00	11.25	04:01	70,00	Patient showed great improvement in pain. Treatment strategy was maintained.	6.43	1.61	45.00	11.25	04:01	CBD-rich Cannabis oil MALELI 3000mg (20%)
	Sacrococcigeal pain	17f	CBD	CBD	0,8	Dry mouth, excessive thirst.	M53.3	Patient presenting pain in the coccix region, plus anxiety and irritability issues. Treatment chosen consists in low to intermediate CBD-rich extract given its antiinflammatory, ansiolitic and pain alleviating properties.	5.42	1.36	45.00	11.25	04:01	81,00	Patient showed great improvement in pain. Treatment strategy was maintained.	5.56	1.39	45.00	11.25	04:01	CBD-rich Cannabis oil MALELI 3000mg (20%)
	Myalgia	9f	THC	CBD	0,4	Drowsiness, dry mouth, excessive thirst, weight gain.	M79.1, M54.2, , F41.1, N80	Patient with tension-related neck pain, bad sleep habits, bruxism and considerable anxiety. Treatment chosen consists of THC-rich extract for its pain alleviating, muscle relaxing and sleep inducing properties. Low to intermediary dose is preferred to avoid worsening anxiety with higher doses.	0.02	0.91	0.16	6,00	01:38	60,00	Patient showed great improvement in pain, anxiety and insomnia, However, work issues caused their anxiety to worsen considerably, despite good pain management. New treatment consists in adding an intermediary dose of CBD-rich extract, given its ansiolitic properties and dampening of potential THC side effects.	5.05	1.24	30.27	7.44	04:01	CBD-rich Cannabis oil AMA+ME 1500mg / CBD-rich Cannabis oil MALELI 1500mg (10%)
Nociplastic / Inflamatory / mixed	Fibromyalgia	7f	THC	THC	1,8	N	M.79.7	Patient diagnosed with fibromyalgia and insomnia. We opted for an intermediate- to low-dose THC-rich cannabis extractact because of its potential to help with the patient's two main complaints.	0.02	0.72	0.20	7.50	01:38	107	The patient presented satisfactory improvement of pain and insomnia. Opted to maintain prescriptive strategy.	0.02	0.70	0.20	7.50	01:38	THC-rich Cannabis oil AMA+ME 900mg (10%)
		15f	CBD	CBD	1,6	N	M.79.7	Patient with fibromyalgia, diffuse joint pain and obesity grade II. Opted for CBD-richcannabis extract for its analgesic, anti-inflammatory potential and also for the potential to reduce appetite and aid in weight loss	4.85	1.21	50.00	12.50	04:01	98	The patient's clinical condition improved very satisfactorily. We chose to maintain a prescriptive strategy and increase the dose to try to help more in the issue of appetite reduction and weight loss.	7.14	1.79	70.00	17.50	04:01	CBD-rich Cannabis oil MALELI 3000mg (20%)
		16f	CBD	CBD	1,6	Drowsiness	M.79.7	Patient with fibromyalgia and anxiety. We opted for CBD-rich cannabis extract. Intermediate dose, due to the potential for improvement in both pain and anxiety	6.92	1.73	45.00	11.25	04:01	65	The patient presented satisfactory improvement of pain and partial anxiety of anxiety, with the dose adjusted to 60mg CBD/day.	9.25	3.23	60.16	21.00	03:01	CBD-rich Cannabis oil MALELI 3000mg (20%) / THC-rich Cannabis oil MALELI 900mg (10%)
		18f	THC	CBD	1,6	N	M.79.7	Patient diagnosed with fibromyalgia, anxiety-depressive mood pattern and insomnia. We opted for an intermediate to low dose DCA rich in THC because of its potential to help with the patient's main complaints.	0.04	1.50	0.27	10.50	01:38	67	After a year of good pain control with the THC-rich OC at an intermediate dose, the patient began to present more intense episodes of pain and worsening of the mood pattern. Opted to add to the treatment, an intermediate to low dose CBD-rich cannabis extract to aid in pain management and improve mood.	4.54	1.52	37.91	10.80	03:01	CBD-rich Cannabis oil AMA+ME 1500mg / THC-rich Cannabis oil AMA+ME 600mg (5%)
		27f	CBD	THC	1,4	N	M79.7	Patient with fibromyalgia and poor sleep pattern. We opted for an intermediate- to low-dose THC-rich cannabis extract because of its potential to help with the patient's two main complaints.	0.03	0.65	0.16	6.00	04:01	80,00	The clinical evolution was very satisfactory. Maintained dosage similar to the first prescription.	0.02	0.75	0.16	6.00	01:38	THC-rich Cannabis oil AMA+ME 900mg (10%)
		1f	CBD	CBD	1,3	N	M.79.7	Patient diagnosed with fibromyalgia. but also with intense diffuse joint pain. Tendency to apathy and prostration during the day. Anxious mood pattern, showing increased appetite and weight gain since the pandemic. I opt for full spectrum cannabis extract rich in CBD, due to the pain character of inflammatory pattern, anxiety, and need for weight reduction.	7.06	1.76	60.00	15.00	04:01	87	The clinical evolution was very satisfactory. Maintained dosage similar to the first prescription.	8.05	2.01	70.00	17.50	04:01	CBD-rich Cannabis oil MALELI 3000mg (20%)
		10f	THC	THC	1	N	M.79.7	Patient diagnosed with fibromyalgia and insomnia. We opted for an intermediate- to low-dose THC-rich cannabis extract because of its potential to help with the patient's two main complaints.	0.04	1.50	0.20	7.50	01:38	45	The patient presented a very satisfactory improvement in pain and insomnia. Opted to maintain prescriptive strategy.	0.08	1.67	0.34	7.50	01:22	THC-rich Cannabis oil MALELI 900mg (10%)
		22f	CBD	CBD	0,9	N	M.79.7	Patient with fibromyalgia and anxiety. Opted for CBD- rich cannabis extract, due to the potential for improvement in both pain and anxiety. Proportionally lower dose, because the patient is very afraid of having any type of side effect from the treatment.	2.42	0.60	15.00	0.72	04:01	62	The patient did not show significant improvement in pain with lower doses of CBD. The dosage was gradually adjusted up to approximately 40mg CBD/day, when the patient reported partial improvement of the condition.	5.43	0.26	37.50	1,8	21:01	CBD-rich Cannabis oil MALELI 3000mg (20%)
		19f	THC	CBD	0,9	N	M.79.7	Patient diagnosed with fibromyalgia, anxiety-depressive mood pattern and insomnia. We opted for an intermediate to low dose cannabis extract rich in THC because of its potential to help with the patient's main complaints.	0.08	1.67	0.32	7.00	01:22	45	The patient presented partial improvement in pain and poor sleep pattern, but did not show improvement in mood. It was decided to add an intermediate to low dose CBD-rich OC to the therapy to optimize pain control and improve mood. And so, also avoid increasing the dose of THC-rich cannabis extract (and its anxiogenic potential at higher doses).	10.14	3.57	45.63	16.07	03:01	CBD-rich Cannabis oil MALELI 3000mg (20%)
	Fibromyalgia pluis joint pain	23f	THC	THC	2.0	N	M79.7, M.25.5	Patient presenting fibromyalgia, joint pain and insomnia. Treatment chosen was low to intermediate THC-rich extract given its potential to alleviate the main issues mentioned.	0.02	0.86	0.16	6.00	01:38	65	Patient showed great improvement in all symptoms. Doses were adjusted along the treatment to favor therapeutics. Treatment strategy was maintained.	0.05	1.85	0.31	12.00	01:38	THC-rich Cannabis oil AMA+ME 900mg (10%)
		2f	THC	CBD	0.7	N	M79.7, M25.5	Patient presenting neuropathic pain after spinal arthrodesis. Posteriorly diagnosed with fibromyalgia as well. Patient then developed erythromelalgia and joint pain in shoulders, knees and hips. Treatment chose was pain alleviating and anti-inflammatory THC- rich extract. Intermediate doses given the pain profile. Higher doses of THC are avoided since there is risk of worsening erythromelalgia (a vasodilator).	0.03	1.20	0.23	9.00	01:38	69	Patient showed great improvement of fibromyalgia and some improvement of the neuropathic pain. However, erythromelalgia got worse. New treatment consists of a new extract rich in CBD in intermediate doses.	13.04	3.26	90.00	22.50	04:01	CBD-rich Cannabis oil MALELI 3000mg (20%)
	Fibromyalgia plus Ciatic pain	14f	CBD	CBD	1.0	N	M54.4, M79.7	Patient presented fibromyalgia and diffuse joint pain. There is relevant anxiety as well. Treatment chosen was low to intermediate dose of CBD-rich extract, given its pain alleviating, anti-inflammatory and ansiolitic properties.	4.93	0.24	37.50	1.80	21:01	69	Patient showed great improvement in pain management. Treatment stretagy was maintained.	5.43	0.26	37.50	1,8	21:01	CBD-rich Cannabis oil AMA+ME 1500mg
Neuropathic	Nerve Roots and Plexuses Disorder	28f	THC	THC	1,4	N	G54	Patient presenting neuropathic and myofascial pain. Treatment chosen consists of intermediary dose of THC-rich extract given its pain alleviating, anti-inflammatory and muscle-relaxing properties.	0.04	1.70	0.23	9.00	01:38	53,00	Patient did not improve a lot with the 9mg THC/day dose. Dose was then adjusted to 18 mg THC/day.	0.09	3.40	0.47	18.00	01:38	THC-rich Cannabis oil MALELI 900mg (10%)
	Algoneurodystrophy	3f	THC	THC	1	N	M89.0	Patient presents regional complex pain after right ankle surgery in 2012. There are insomnia issues and incapacitating pain. There are few entries in the literature for the use of cannabinoids in regional complex pain treatment, but they, plus clinical experience, help support the use of THC as a treatment option. Generally, low to intermediate doses bring surprisingly satisfactory results.	0.01	0.57	0.13	5.00	01:38	88,00	At approximately 5mg THC/day the patient improved greatly in sleep, anxiety and irritability. However, there was little change in pain symptoms. Dose was then changed to 9mg THC/day and the patient showed considerable improvement in pain. They could also stop taking fluoxetine, pregabaline and amitriptline without issues.	0.03	1.02	0.23	9.00	01:38	THC-rich Cannabis oil MALELI 600mg (5%)
	Lumbago with Sciatica	25f	CBD	CBD	0,6	N	M54.4	Patient presenting lower back pain and longstanding lumbosciatica, plus anxiety symptoms. Treatment chosen consists of intermediate doses of CBD-rich extract pain alleviating, anti-inflammatory and ansiolitic properties.	6.67	1.67	60.00	15.00	04:01	90,00	Patient presented satisfactory improvements in pain and anxiety. Treatment strategy was maintained.	7.78	1.99	60.00	15.00	04:01	CBD-rich Cannabis oil AMA+ME 1500mg / THC-rich Cannabis oil AMA+ME 900mg (10%) / CBD-rich Cannabis oil MALELI 3000mg (20%) / THC-rich Cannabis oil MALELI 900mg (10%)
		12f	CBD	CBD	0,5	N	M54.5, M54.4	Patient presenting lower back pain and longstanding lumbosciatica, plus anxiety symptoms. Treatment chosen consists of low-to-intermediate doses of CBD- rich extract pain alleviating, anti-inflammatory and ansiolitic properties.	4.69	0.23	37.50	1.80	21:01	80,00	The patient showed satisfactory improvement of the pain, but still had intense anxious symptoms. It was decided to adjust the dose of CBD-rich extract to approximately 50mg CBD/day, resulting in an improvement in anxiety.	6.56	0.32	52.50	2.52	21:01	CBD-rich Cannabis oil AMA+ME 1500mg
