# Supplementary material for: Full-spectrum cannabis extracts for women with chronic pain syndromes: a real-life retrospective report of multi-symptomatic benefits after treatment with individually tailored dosage schemes
Source: Front Pharmacol. 2025 Nov 20;16:1538518. doi: 10.3389/fphar.2025.1538518 (PMC12675365; doi:10.3389/fphar.2025.1538518)
Supplement: Supplementary file 5 [file DataSheet5.pdf]

ORIENTAÇÕES AOS PRESCRITORES

Sumário

1. Apresentação 2
2. Serviço de Produção de Óleo Full Spectrum Maria Flor 3
3. Óleos de Cannabis full spectrum rico em THC 4
.... 4
4. Óleos de Cannabis full spectrum rico em CBD 5
5. Óleos de Cannabis full spectrum CBD:THC (1:1) 6
6. Orientações ao Prescritor 7
7. Orientações ao Paciente 8

1. *Apresentação*

Caro prescritor,

A Associação Canábica Maria Flor, se preocupa com o tratamento de seus associados, buscamos uma padronização de dosagens a fim de facilitar a compreensão tanto dos pacientes como dos profissionais de saúde, contribuindo assim para a continuidade emanutenção do tratamento.

A cada lote de extrato produzido, é feita uma análise cromatográfica para dosar a quantidade de fitocanabinóide por ***mL de extrato***, o que permite que as dosagens dos tratamentos dos associados sejam mantidas mesmo durante atroca de um lote de extrato para o outro.

A Maria Flor produz hoje cinco produtos a base de Cannabis que são destinados **exclusivamente para uso médico de seus Associados**. São eles:

Óleos de Cannabis *full spectrum* rico em **THC**

Óleos de Cannabis *full spectrum* rico em **CBD**

Óleos de Cannabis *full spectrum* **CBD:THC (1:1)**

Pomada de Cannabis

Sabonete de Cannabis

Nas paginas seguintes vocês irão encontrar mais detalhes sobre os extratos produzidos, suas diluições e quais orientações são importantes para os novos Associados.

CNPJ 37.427.678/0001-65 | Caixa Postal: 1017 | CEP: 17.506-040 – Marília SP
administrativo@mariaflor.org.br | www.mariaflor.org.br | (14) 99154 9400

2. Serviço de Produção de Óleo Full Spectrum Maria Flor

***custo envolvido desde o plantio, extração e produção de óleo full spectrum a base de Cannabis para Associados apenas.**

- Óleo de Cannabis *full spectrum* rico em THC 300mg - 30ml................................ R$ 150
- Óleo de Cannabis *full spectrum* rico em THC 600mg - 30ml................................ R$ 250
- Óleo de Cannabis *full spectrum* rico em THC 900mg - 30ml................................ R$ 350
- Óleo de Cannabis *full spectrum* CBD:THC (1:1) 600mg 30ml..............................R$ 300
- Óleo de Cannabis *full spectrum* rico em CBD 1500mg – 30mL........................... R$ 350
- Sabonete de glicerina com extrato de Cannabis – 100g...R$ 35
- Pomada de Cannabis 3% - 30 g .... R$ 75

CNPJ 37.427.678/0001-65 | Caixa Postal: 1017 | CEP: 17.506-040 – Marília SP
administrativo@mariaflor.org.br | www.mariaflor.org.br | (14) 99154 9400

3. *Óleos de Cannabis full spectrum rico em THC*

Produto	*Concentração/mL*	*Concentração/gota*
**THC 300mg**	10mg/mL	0,5mg/gota
**THC 600mg**	20mg/mL	1mg/gota
**THC 900mg**	30mg/mL	1,5mg/gota

a. *Análise cromatográfica*

Cromatograma e resultados

Detalhes da Análise			
Nome	Maria Flor/Maleli Lote: THC030	Data	17/11/2021

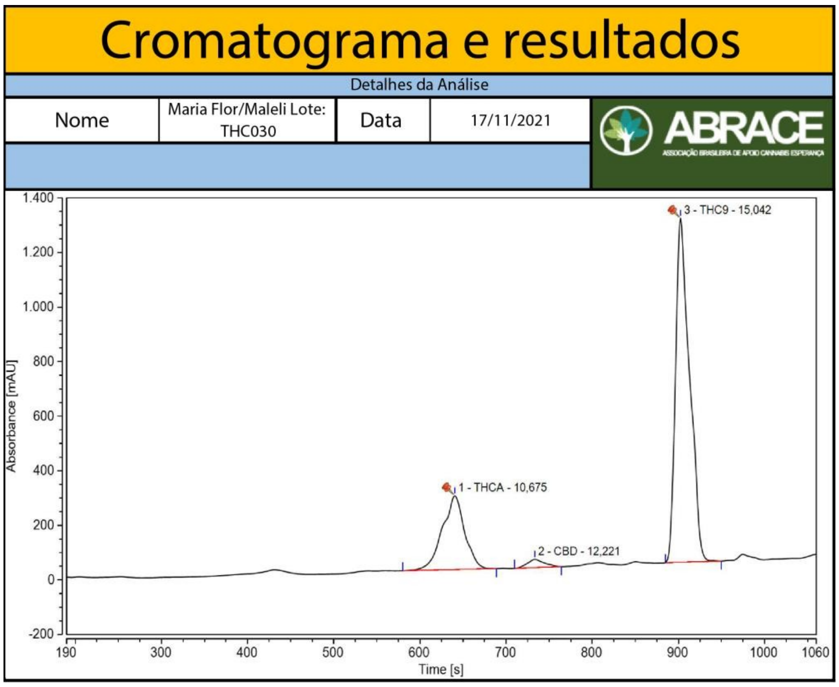

Resultados			
Substância	Concentração (mg/g)	% *	Proporção THC/CBD
THC-A	131,63	13,2%	38,6:1
THC	626,29	62,6%	
CBD-A	0,00	0,0%	
CBD	16,22	1,6%	
CBN	0,00	0,0%	
THC total	757,9	75,8%	
CBD total	16,2	1,6%	
Canabinóides total	774,1	77,4%	

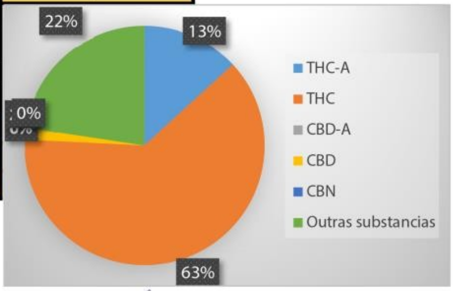

Kaio Aragão Sales

Farmacêutico - Controle de Qualidade - CRF-PB 03770

CNPJ 37.427.678/0001-65 | Caixa Postal: 1017 | CEP: 17.506-040 – Marília SP
administrativo@mariaflor.org.br | www.mariaflor.org.br | (14) 99154 9400

4. *Óleos de Cannabis full spectrum rico em CBD*

Produto	*Concentração/mL*	*Concentração/gota*
**CBD 1.500mg**	50mg/ml	2,5mg/gota

2.2 Análise Cromatográfica

Cromatograma e resultados

Detalhes da Análise

Nome	Maleli Lote: CBD001	Data	12/01/2022

ABRACE
ASSOCIAÇÃO BRASILEIRA DE APOIO CANNABIS ESPERANÇA

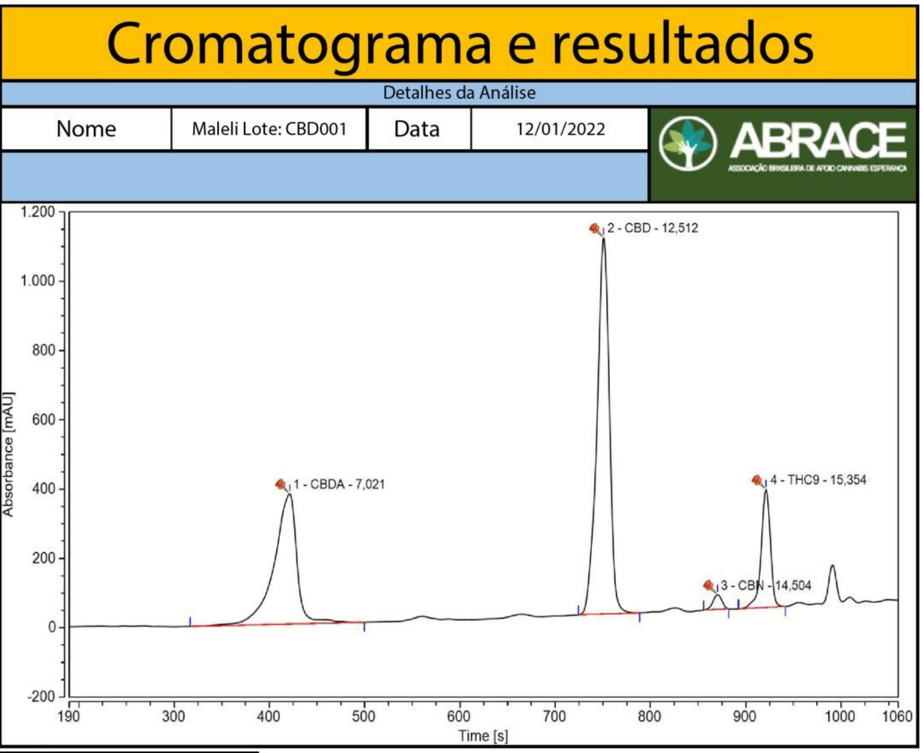

Resultados

Substância	Concentração (mg/ç)	% *
THC-A	0,00	0,0%
THC	100,75	10,1%
CBD-A	144,17	14,4%
CBD	388,22	38,8%
CBN	4,82	0,5%
THC total	100,7	10,1%
CBD total	532,4	53,2%
Canabinóides total	633,1	63,8%

Proporção CBD/THC
3,9:1

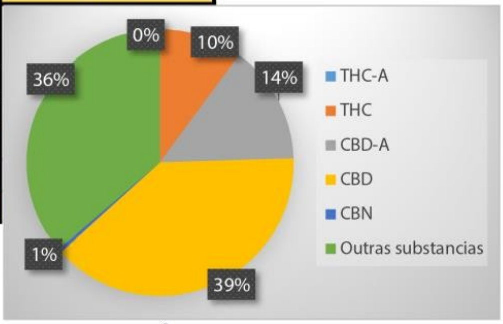

Kaio Aragão Sales
Farmacêutico - Controle de Qualidade - CRF-PB 03770

CNPJ 37.427.678/0001-65 | Caixa Postal: 1017 | CEP: 17.506-040 – Marília SP
administrativo@mariaflor.org.br | www.mariaflor.org.br | (14) 99154 9400

5. *Óleos de Cannabis full spectrum CBD:THC (1:1)*

Produto	*Concentração/mL*	*Concentração/gota*
**CBD : THC 600mg**	8,6mg/mL CBD: 11,4mg/mL THC	CBD 0,43mg/gota<br>THC 0,57mg/gota

- ***O frasco com 30mL de óleo CBD:THC contém 258mg de CBD e 342mg de THC.***

a. *Análise Cromatográfica*

Cromatograma e resultados

Detalhes da Análise			
Nome	Maria Flor/Maleli Lote: MFL001	Data	17/11/2021

ABRACE
ASSOCIAÇÃO BRASILEIRA DE APOIO CANNABIS ESPERANÇA

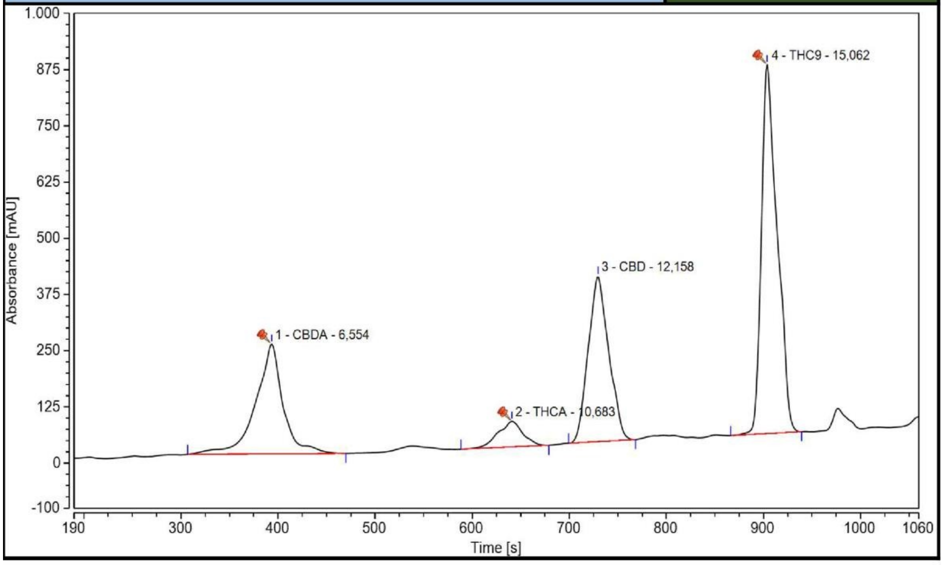

Resultados			
Substância	Concentração (mg/g)	% *	Proporção THC/CBD
THC-A	31,34	3,1%	1,9:1
THC	407,09	40,7%	
CBD-A	109,73	11,0%	
CBD	213,71	21,4%	
CBN	0,00	0,0%	
THC total	438,4	43,8%	
CBD total	323,4	32,3%	
Canabinóides total	761,9	76,2%	

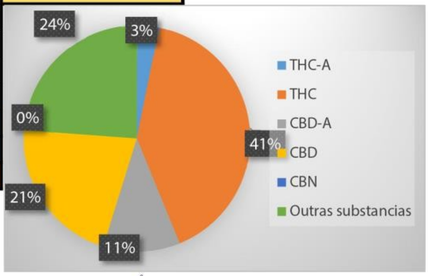

Kaio Aragão Sales
Farmacêutico - Controle de Qualidade - CRF-PB 03770

CNPJ 37.427.678/0001-65 | Caixa Postal: 1017 | CEP: 17.506-040 – Marília SP
administrativo@mariaflor.org.br | www.mariaflor.org.br | (14) 99154 9400

6. *Orientações ao Prescritor*

MODELO DE RECEITA

Receituário

Controle especial

Uso Oral (Sublingual)

- Óleo de Cannabis *full spectrum* rico em **CBD 1500mg** ------------01 frasco

- Óleo de Cannabis *full spectrum* rico em **THC 300mg**--------- -----01 frasco
  THC 600mg
  THC 900mg

- Óleo de Cannabis *full spectrum* **CBD:THC (1:1) 600mg** ------------01 frasco

Uso tópico

- Pomada de Cannabis
- Sabonete com extrato de Cannab

Data e Assinatura do médico

MODELO DE LAUDO

Laudo médico

Paciente _________________, portador do CID-10 ___________, faço indicação do uso de Cannabis / terapia com fitocanabinóides / extrato de Cannabis.

Data ou vencimento do laudo,

Assinatura do médico

mariaflor
Associação Canábica

7. *Orientações ao Paciente*

Nossos produtos são exclusivamente de uso médico dos nossos Associados.

Para efetuar o cadastro junto a associação, alguns DOCUMENTOS SÃO OBRIGATÓRIOS.

- RG, CPF ou CNH
- Comprovante de residência
- Receita médica/veterinária/odontológica contendo a prescrição do óleo Maria Flor
- Laudo médico para o tratamento com Cannabis
- Contribuição da Taxa de Adesão Anual no valor de R$ 240 (a vista ou parcelado em até 10x)
- Termo de Ajuizamento e Corresponsabilidade **assinado.**

Todos os atendimentos do Núcleo de Acolhimento são feitos via Whatsapp, por ordem de chegada.

**Central do Núcleo de Acolhimento (Cris)**	**14 99154 9400**
Amanda	14 99891 5263
Rodler	14 99674 5687
Marina	14 99735 3445
Cláudia	14 99897 2191

O **CADASTRO** é feito on-line, pelo site www.mariaflor.org.br (Área do Paciente > Cadastro).

É importante que o paciente PROCURE UM ACOLHEDOR para seguir com o Pedido após a realização do Cadastro.

O Pedido só poderá ser realizado após a conclusão do Cadastro do Associado e pagamento da Taxa Anual.

Dra Caroline Marroni Cremonez
Farmacêutica-Toxicologista (FCFRP-USP)
Presidente e Diretora Cientifica da Associação Canábica Maria Flor

CNPJ 37.427.678/0001-65 | Caixa Postal: 1017 | CEP: 17.506-040 – Marília SP
administrativo@mariaflor.org.br | www.mariaflor.org.br | (14) 99154 9400

Flor é símbolo de vida.
De beleza, de afeto, de cuidado.
Mais do que símbolo, flor
É vida.

São as flores que semeiam a terra.
E garantem a continuidade da vida.
Delas, também extraímos muito para nós.
Sabores, aromas, cores.
Cura.

As flores e as plantas nascem livres.
E devem crescer e dar frutos assim: livres.
Como você.
Você e quem você ama têm o direito de florescer.
De viverem com o máximo de qualidade.
De serem felizes da forma que escolherem.
Mas isso não acontece de uma hora pra outra.

Por que qualidade de vida não se compra.
Qualidade de vida se cultiva.

Nós cultivamos flores para alimentar o ciclo da vida.
E oferecer o melhor dela para você.
Vem cultivar. Vem ser flor.

Maria Flor
Vida cultivada.

CNPJ 37.427.678/0001-65 | Caixa Postal: 1017 | CEP: 17.506-040 – Marília SP
administrativo@mariaflor.org.br | www.mariaflor.org.br | (14) 99154 9400
