# Supplementary figures and images for: Full-spectrum cannabis extracts for women with chronic pain syndromes: a real-life retrospective report of multi-symptomatic benefits after treatment with individually tailored dosage schemes
Source: Front Pharmacol. 2025 Nov 20;16:1538518. doi: 10.3389/fphar.2025.1538518 (PMC12675365; doi:10.3389/fphar.2025.1538518)

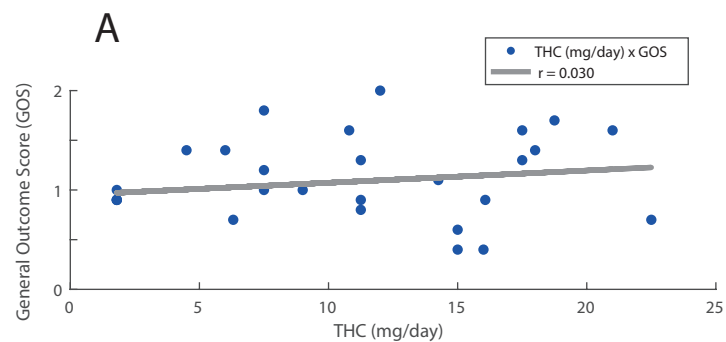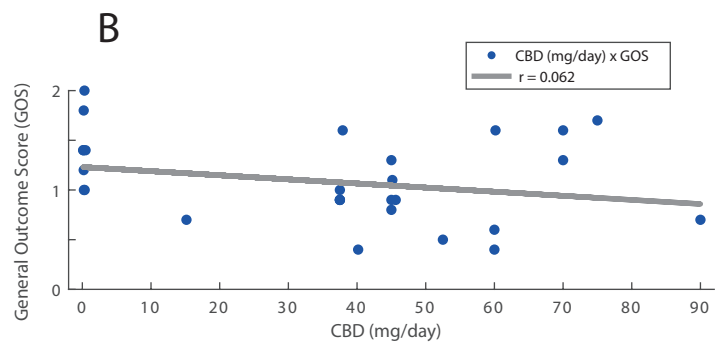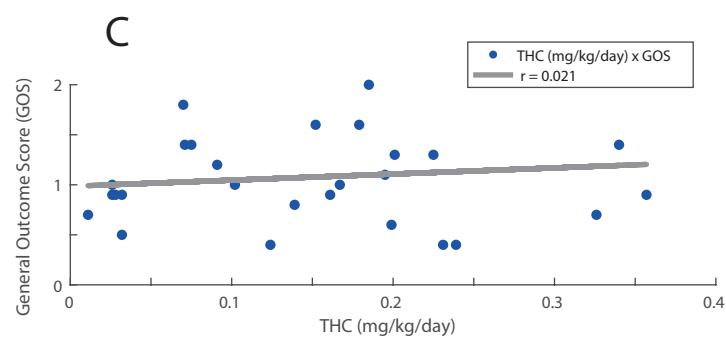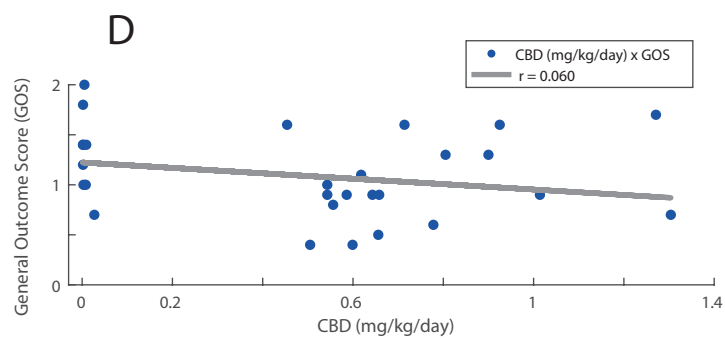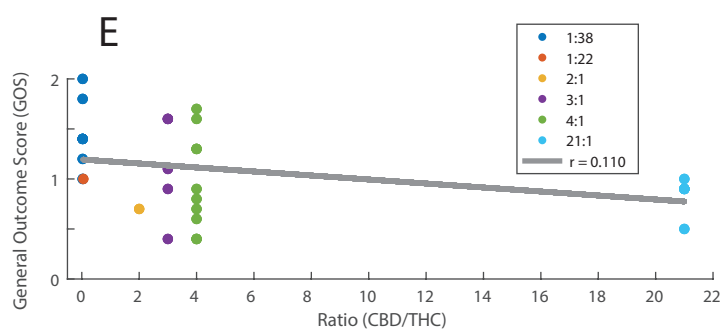

Supplement: Supplementary file 6 [file Image1.pdf]
